# Changes in the US Burden of Chronic Kidney Disease From 2002 to 2016: An Analysis of the Global Burden of Disease Study

**DOI:** 10.1001/jamanetworkopen.2018.4412

**Published:** 2018-11-30

**Authors:** Benjamin Bowe, Yan Xie, Tingting Li, Ali H. Mokdad, Hong Xian, Yan Yan, Geetha Maddukuri, Ziyad Al-Aly

**Affiliations:** 1 Clinical Epidemiology Center, Research and Education Service, Veterans Affairs St Louis Health Care System, St Louis, Missouri; 2 Department of Epidemiology and Biostatistics, College for Public Health and Social Justice, St Louis University, St Louis, Missouri; 3 Department of Medicine, Washington University in St Louis School of Medicine, St Louis, Missouri; 4 Institute for Health Metrics and Evaluation, University of Washington, Seattle; 5 Division of Public Health Sciences, Department of Surgery, Washington University in St Louis School of Medicine, St Louis, Missouri; 6 Nephrology Section, Medicine Service, Veterans Affairs St Louis Health Care System, St Louis, Missouri; 7 Institute for Public Health, Washington University in St Louis, St Louis, Missouri

## Abstract

**Introduction:**

Over the past 15 years, changes in demographic, social, and epidemiologic trends occurred in the United States. These changes likely contributed to changes in chronic kidney disease (CKD) epidemiology.

**Objective:**

To describe the change in burden of CKD at the US state level from 2002 to 2016.

**Design, Setting, and Participants:**

This systematic analysis used data and methodologies from the 2016 Global Burden of Disease study in the United States. Data on CKD from 2002 to 2016 were examined at the state level.

**Main Outcomes and Measures:**

Disability-adjusted life years (DALYs) and death due to CKD.

**Results:**

In this analysis of data from individuals in the United States, from 2002 to 2016, CKD DALYs increased by 52.6%, from 1 269 049 DALYs (95% uncertainty interval [UI], 1 154 521-1 387 008) to 1 935 954 DALYs (95% UI, 1 747 356-2 124 795). Death due to CKD increased by 58.3%, from 52 127 deaths (95% UI, 51 082-53 076) to 82 539 deaths (95% UI, 80 298-84 652). All states exhibited increases in CKD burden, but the rate of change (2002-2016) and the burden in 2016 varied by state. States in the southern United States (including Mississippi and Louisiana) exhibited more than twice the burden seen in other states (eg, the age-standardized CKD DALY rate in Vermont was 321 [95% UI, 281-363] per 100 000 population, whereas the rate in Mississippi was 697 [95% UI, 620-779] per 100 000 population). In the United States, the increase in CKD DALYs was attributable to increased risk exposure (40.3%), aging (32.3%), and population growth (27.4%). Age-standardized CKD DALY rates increased by 18.6% where increases in metabolic, and to a lesser extent dietary, risk factors contributed 93.8% and 5.3% of this change, respectively. Chronic kidney disease due to diabetes was the primary contributor for the 26.8% increased probability of death due to CKD among the population aged 20 to 54 years; among the population aged 55 to 89 years, the probability of death due to CKD increased by 25.6% and was driven by CKD due to diabetes and decreased probability of death from causes other than CKD. Improvement in sociodemographic development was coupled with an increase in age-standardized CKD DALY rates that occurred at a faster pace than that of other noncommunicable diseases in the United States.

**Conclusions and Relevance:**

Our findings revealed that between 2002 and 2016, the burden of CKD in the United States appeared to be increasing and variable among states. These changes may be associated with increased risk exposure and demographic expansion leading to increased probability of death due to CKD, especially among young adults. The findings suggest that an effort to target the reduction of CKD through greater attention to metabolic and dietary risks, especially among younger adults, is necessary.

## Key Points

**Question:**

How did the burden of chronic kidney disease (CKD) change in the United States from 2002 to 2016?

**Findings:**

This analysis of the Global Burden of Disease 2016 study data revealed that, from 2002 to 2016, the US burden of CKD increased and at a faster pace than other noncommunicable diseases; the burden was highly variable among states (some exhibited more than twice the burden as others). The increase in burden was associated with increased metabolic and dietary risk exposure that manifested in increased probability of death due to diabetic CKD, especially among the population aged 20 to 55 years.

**Meaning:**

The findings suggest the need to target chronic kidney disease burden by addressing risk exposure among young adults.

## Teaser

This analysis of Global Burden of Disease 2016 study data and methodologies describes the change in burden of chronic kidney disease at the US state level from 2002 to 2016.

## Introduction

In the past 15 years, demographic, social, and epidemiologic changes have occurred in the United States.^1^ From 2002 to 2016, the US population grew from 287 million to 323 million and life expectancy increased from 76.8 to 78.8 years.^2,3^ In addition, measures of sociodemographic development and exposure to risk factors for chronic kidney disease (CKD) have substantially increased over the same period.^4,5,6^

Population growth, aging, changes in sociodemographic development, and risk factor epidemiologic changes have likely contributed to changes in the burden of CKD. However, a detailed quantitative analysis of the change in burden of CKD over the past 15 years at the state level is not available. In this study, the Global Burden of Disease (GBD) study data and methodologies were used to (1) describe the change in the burden of CKD in the United States from 2002 to 2016, (2) characterize factors associated with change in CKD burden, and (3) examine how sociodemographic progress has shaped the burden of CKD.

## Methods

The analysis was approved by the institutional review board of the Veterans Affairs St Louis Health Care System. There was no informed consent in this study in accordance with Veterans Health Administration guidance on publicly available data. A glossary of GBD terms used in this work are defined in eTable 1 in the Supplement. This report follows the Guidelines for Accurate and Transparent Health Estimates Reporting (GATHER) guidelines for reporting health estimates.^7^

### Data Sources

In this study, we used GBD 2016 data on the US population available from the GBD results tools.^8^ The GBD 2016 study provides detailed epidemiologic assessment of 333 diseases and injuries and 84 risk factors by age and sex on a global scale for 195 countries and territories and, for certain countries, subnational estimates (including the 50 states and 1 district of the United States).^4,8,9,10,11^ In this study, GBD 2016 data from 2002, when the US National Kidney Foundation introduced guidelines for CKD diagnosis, until 2016 were used.^12^ Detailed descriptions of overall GBD 2016 methodologies and the specific CKD methodology have been provided elsewhere.^4,9,10,11,13,14^

In the GBD 2016, CKD was defined as an estimated glomerular filtration rate (eGFR) less than 60 mL/min/1.73 m^2^. Data used for estimating mortality due to CKD were obtained from the US National Vital Statistics System’s Custom Mortality Data and United States Military Deaths and the United States Renal Data System’s Annual Data Reports.^10^ Garbage code redistribution applied to underlying cause of death (eTable 1 in the Supplement), which reattributes the causes of death that cannot or should not be an underlying cause of death, accounts for changes in coding by geography and across time.^10,15^ A Cause of Death Ensemble model (CODEm) was used to estimate death rates, in which out-of-sample prediction correction methodology was used to further refine the model and validate estimates. Estimates were then processed through Cause of Death Correct (CoDCorrect) to reconcile estimates with those of other causes of death.^15,16^

The proportion of CKD mortality attributable to diabetes, hypertension, glomerulonephritis, and other causes was informed by end-stage renal disease registries and modeled in DisMod-MR 2.1, an integrative metaregression method that evaluates all available information on a disease that passes quality standards,^1,14^ to obtain location, sex-, age-, and year-specific estimates. Nonfatal estimates are based on data from the US National Health and Nutrition Examination Survey, US Renal Data System’s Annual Data Reports, and other population-based research studies and were estimated by DisMod-MR 2.1.^1,14^ Risk factors were defined based on the comparative risk assessment framework, in which risks of CKD were hierarchically organized, and their contribution could be quantified at any level of the framework. Risk-outcome pairs are included only if they met World Cancer Research Fund International criteria for convincing or probable evidence.^1,4^ Further details on estimations of cause of death, nonfatal burden and risk factors are given in the eAppendix in the Supplement.

### Measures of Burden

Measures of CKD burden, including number and age-standardized rate for years of life lost (YLL), years living with disability (YLD), disability-adjusted life years (DALYs), and deaths were used in analyses. Years living with disability captures years lived with less-than-ideal health because of CKD and was estimated by a multiplication of prevalent cases of CKD and a disability weight.^1,11^ Years of life lost is a measure of the years lost owing to premature mortality due to CKD and was based on the remaining life expectancy compared with a reference standard life table at age of death.^11^ Disability-adjusted life years was calculated through the summation of YLD and YLL (eTable 1 in the Supplement). Age standardization of rates was based on a time-invariant world standard population developed for the GBD study.^10^ Rates by cause of CKD are reported as age-standardized rates. All rates are reported per 100 000 population. Change of the burden from 2002 to 2016 was reported as absolute change (the value in 2016 minus the value in 2002) and percent change (the difference between the value in 2016 and the value in 2002 divided by the value in 2002).

Data on the sociodemographic index (SDI) were also curated from GBD databases to examine the relationship between SDI and disease burden. The SDI is a standardized composite summary measure—comparable across geographies and over time—of mean income per capita, educational attainment, and total fertility rate at the state level^5^ (eTable 1 in the Supplement). The SDI ranges from 0 (low) to 1 (high), with a higher index indicating greater sociodemographic development.

### Decomposition Analyses

To investigate explanatory factors associated with changes in CKD DALYs and deaths in the United States from 2002 to 2016, decomposition analyses according to the methods of Das Gupta^17^ were developed, in which the percent change contributed by each factor was computed by decomposing (1) the changes in DALYs by population size, age structure, and risk exposure; (2) change in age-standardized DALY rate by CKD risk exposure as defined in the GBD comparative risk assessment framework,^4^ which includes metabolic (high fasting plasma glucose level, high systolic blood pressure, and high body mass index), dietary (diet high in sodium, diet high in sugar-sweetened beverages), and environmental (lead exposure) risk factors; (3) change in age-standardized DALY rate by CKD causes of diabetes, hypertension, glomerulonephritis, and other^17,18,19^; and (4) change in probability of death by 4 causes of CKD (diabetes, hypertension, glomerulonephritis, and other) and epidemiologic changes (non-CKD causes of death), using an abridged multiple decrement period life table for 2 summary age intervals (20-54 years and 55-89 years). These age groups were selected to reflect changes in the probability of death among young adults and older people. Further details are given in the eAppendix in the Supplement.

### Observed-to-Expected and Frontier Analyses

Expected age-standardized DALY and death rates based on SDI for each state in 2016 were estimated and compared with the observed DALY rate in 2016 on a ratio scale to assess deviation from expectations of burden based on a state’s or district’s level of sociodemographic development. To calculate the estimated value, generalized estimating equations using state data from 2002 to 2016 were built. A frontier analysis was undertaken to quantitatively identify the lowest potentially achievable age-standardized CKD DALY rates at the state level based on the SDI in 2016.^20,21^ Distance from the frontier suggests the potential for unrealized gain or improvement (reduction in CKD DALYs) that should be possible based on the state’s place on the development spectrum. Further details on the observed-to-expected and frontier analyses are included in the eAppendix in the Supplement.

### Statistical Analysis

Measures of burden are presented with 95% uncertainty intervals (UIs), which not only account for variance in parameter estimation but also incorporate uncertainty owing to data collection, model selection, and other sources of uncertainty during the estimation process. In the observed-to-expected analysis, Wald χ^2^ tests were used to assess potential interaction between SDI and time and the significance of splines of SDI and time when testing deviation from linearity. A 2-sided *P* < .05 was considered to be statistically significant. Statistical software SAS Enterprise Guide (SAS Institute), version 7.1, was used for all analyses. ArcMap (Environmental Systems Research Institute) was used to generate maps of the United States. Tableau (Tableau Software) was used for all other image generation.

## Results

In the United States, CKD DALYs were 1 269 049 (95% UI, 1 154 521-1 387 008) in 2002 and increased to 1 935 954 (95% UI, 1 747 356-2 124 795) in 2016; representing a 52.6% increase over the 15-year study period (eTable 2 in the Supplement). The DALY rates increased by 35.9%, from 441 per 100 000 population (95% UI, 401-482 per 100 000 population) to 600 per 100 000 population (95% UI, 541-658 per 100 000 population). Age-standardized DALY rates increased by 18.6%, from 371 per 100 000 population (95% UI, 336-406 per 100 000 population) in 2002 to 440 per 100 000 population (95% UI, 395-485 per 100 000 population) in 2016 (eTable 2 in the Supplement). Analysis of the change in age-standardized DALY rates by the 4 causes of CKD revealed an increase in the age-standardized DALY rate of CKD due to diabetes (21.8%), CKD due to hypertension (22.0%), CKD due to glomerulonephritis (10.4%), and CKD due to other causes (10.3%) (eTable 2 in the Supplement).

In the overall United States, deaths due to CKD increased from 52 127 (95% UI, 51 082–53 076) in 2002 to 82 539 (95% UI, 80 298-84 652) in 2016, representing a 58.3% increase in deaths due to CKD over the 15-year study period (eTable 3 in the Supplement). From 2002 to 2016, deaths due to CKD increased by 41.1%, from 18 per 100 000 population (95% UI, 18-18 per 100 000 population) to 26 per 100 000 population (95% UI, 25-26 per 100 000 population); the age-standardized death rate increased by 17.9%, from 14 per 100 000 population (95% UI, 14-14 per 100 000 population) to 16 per 100 000 population (95% UI, 16-17 per 100 000 population) (eTable 3 in the Supplement). Age-standardized death rates increased for CKD due to diabetes by 20.0%, hypertension by 19.8%, glomerulonephritis by 11.1%, and other causes by 11.0% (eTable 3 in the Supplement).

Additional estimates of YLD, YLL, DALYs, and death due to CKD in the United States and in each state for the overall population and in males and females are provided in eTables 4 to 7 in the Supplement.

### Burden of CKD by State

#### DALYs by State

**Figure 1.**
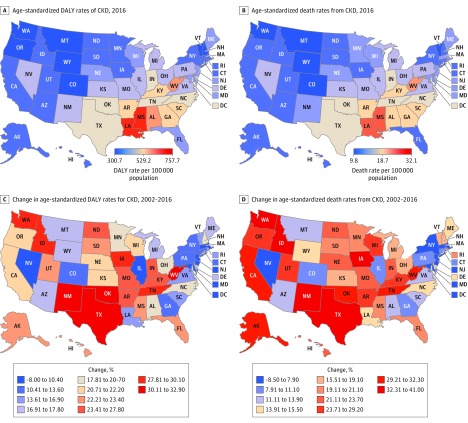
Maps of Age-Standardized Disability-Adjusted Life Years (DALYs) and Death Rates Due to Chronic Kidney Disease (CKD) in 2016, and Percentage Change From 2002 to 2016 Maps of percentage change are colored by deciles of their respective values.

In 2016, the states with the highest age-standardized DALY rates per 100 000 population were (in descending order) Mississippi (697; 95% UI, 620-779), Louisiana (681; 95% UI, 619-749), Alabama (604; 95% UI, 534-673), West Virginia (587; 95% UI, 529-647), Georgia (560; 95% UI, 497-625), Arkansas (553; 95% UI, 498-611), South Carolina (550; 95% UI, 488-611), Kentucky (550; 95% UI, 492-605), Indiana (515; 95% UI, 457-574), and North Carolina (515 ;95% UI, 463-570) (Figure 1A and eTable 2 in the Supplement). The states with the lowest age-standardized DALY rates per 100 000 population were (in ascending order) Vermont (321; 95% UI, 281-363), Washington (328; 95% UI, 285-369), Colorado (331; 95% UI, 287-373), Montana (333; 95% UI, 288-377), Oregon (342; 95% UI, 296-384), Wyoming (343; 95% UI, 297-389), New Hampshire (343; 95% UI, 299-389), Iowa (349; 95% UI, 304-396), Rhode Island (355; 95% UI, 307-403), and Connecticut (356; 95% UI, 312-404) (Figure 1A and eTable 2 in the Supplement). Of note, Mississippi (the state with the highest burden) had twice the age-standardized CKD DALY rate compared with Vermont (the state with the lowest burden) (Figure 1A and eTable 2 in the Supplement). The DALYs in 2016 in females and males are provided in eFigure 1 and eTable 6 in the Supplement.

From 2002 to 2016, all states exhibited an increase in CKD DALYs. However, there was a difference in the magnitude of increase in age-standardized CKD DALY rates ranging from 32.9% in Oklahoma to 6.3% in Nevada. The top 10 states that exhibited the largest increase in age-standardized CKD DALY rates included (in descending order) Oklahoma (32.9%), West Virginia (31.3%), Texas (30.9%), New Mexico (30.7%), Iowa (30.1%), Washington (28.5%), Idaho (28.2%), Tennessee (27.9%), Arkansas (27.8%), and Kentucky (26.3%) (Figure 1C and eTable 2 in the Supplement). The 10 states with the least increase in age-standardized CKD DALY rates were (in ascending order) Nevada (6.3%), New Jersey (6.8%), Massachusetts (8.8%), Maryland (9.3%), Illinois (10.4%), New York (10.8%), Connecticut (11.3%), Pennsylvania (12.0%), Georgia (12.7%), and Colorado (13.6%) (Figure 1C and eTable 2 in the Supplement). Changes in DALY rates in females and males are provided in eFigure 1 and eTable 6 in the Supplement.

#### Death by State

In 2016, age-standardized death rates varied among states. The 10 states with the highest age-standardized CKD death rates per 100 000 population included (in descending order) Louisiana (28; 95% UI, 26-30), Mississippi (27; 95% UI, 24-30), West Virginia (23; 95% UI, 22-25), Alabama (23; 95% UI, 20-25), Georgia (22; 95% UI, 20-25), Kentucky (22; 95% UI, 20-24), South Carolina (21; 95% UI, 19-24), Arkansas (21; 95% UI, 20-23), Indiana (21; 95% UI, 19-23), and North Carolina (20; 95% UI, 18-21) (Figure 1B and eTable 3 in the Supplement). The 10 states with the lowest age-standardized CKD death rates per 100 000 population included (in ascending order) Vermont (11; 95% UI, 10-12), Montana (11; 95% UI, 10-13), Washington (12; 95% UI, 11-13), Wyoming (12; 95% UI, 11-13), Colorado (12; 95% UI, 11-13), New York (12; 95% UI, 11-13), South Dakota (12; 95% UI, 11-14), Oregon (12; 95% UI, 12-13), New Hampshire (12; 95% UI, 11-14), and Iowa (13; 95% UI, 11-14) (Figure 1B and eTable 3 in the Supplement). Age-standardized death rates were 2.4-fold higher in Louisiana (28 per 100 000 population; 95% UI, 26-30 per 100 000 population) compared with Vermont (11 per 100 000 population; 95% UI, 10-12 per 100 000 population) (Figure 1B and eTable 3 in the Supplement). Deaths in 2016 among females and males are provided in eFigure 2 and eTable 7 in the Supplement.

From 2002 to 2016, the rate of change in age-standardized death rates varied among states and ranged from 41.0% in Iowa to −2.8% in Nevada. The top 10 states with the highest rate of change were (in descending order) Iowa (41.0%), Washington (38.1%), Idaho (34.6%), Texas (32.9%), New Mexico (32.4%), Oklahoma (32.3%), Tennessee (31.6%), California (31.5%), West Virginia (30.6%), and Alaska (30.1%) (Figure 1D and eTable 3 in the Supplement). The 10 states that exhibited the smallest rate of change in age-standardized death rates were (in ascending order) Nevada (−2.8%), New Jersey (2.9%), Massachusetts (5.4%), Maryland (7.4%), New York (7.9%), Colorado (9.3%), Connecticut (9.3%), Pennsylvania (10.1%), Georgia (10.6%), and Illinois (11.1%) (Figure 1D and eTable 3 in the Supplement). Changes in death rates in females and males are provided in eFigure 2 and eTable 7 in the Supplement.

### Decomposition Analyses

#### Change in DALYs by Risk Exposure, Age Structure, and Population Size

In the United States, from 2002 to 2016, there was a 52.6% increase in DALYs, of which 40.3% was attributable to increased risk exposure, 32.3% to aging, and 27.4% to population growth (eFigure 3 in the Supplement). Decomposition analyses of death showed consistent results and are provided in eFigure 3 in the Supplement.

#### Change in Age-Standardized DALY Rates by Risk Factor Exposure

**Figure 2.**
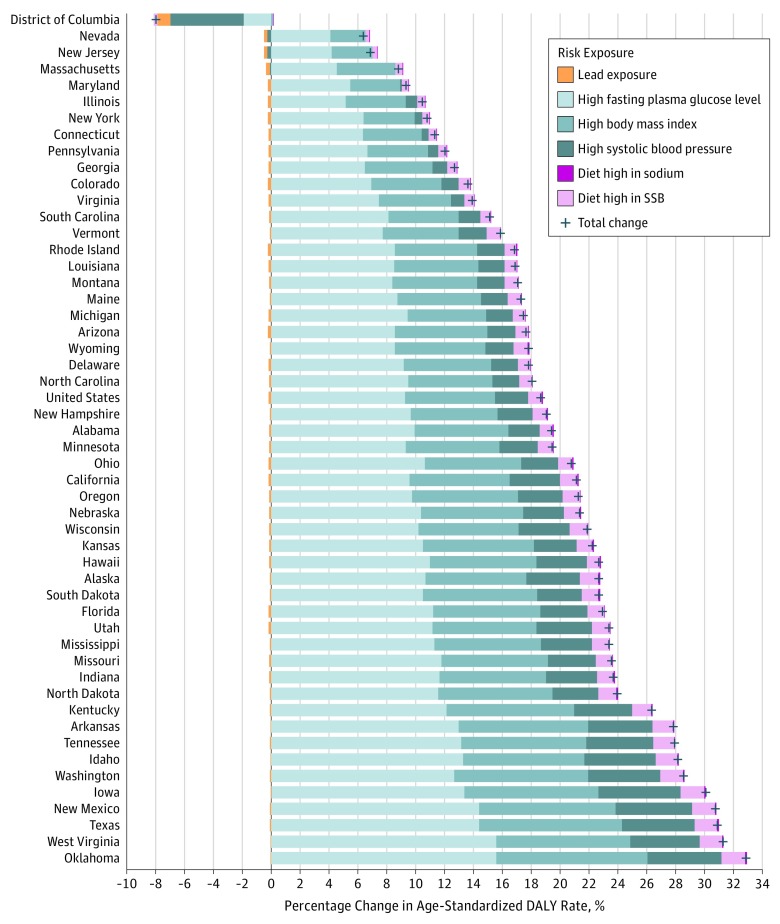
Decomposition of the Percentage Change in Age-Standardized Disability-Adjusted Life Year (DALY) Rates of Chronic Kidney Disease Due to Risk Factors in the United States, 2002-2016 Geographical areas are ordered by total percentage change. Risk exposures are colored similarly to others in the same risk exposure category (environmental, metabolic, and dietary risk factors). SSB indicates sugar-sweetened beverages.

Decomposition analyses of age-standardized DALY rates by GBD risk factors showed reductions of CKD rates caused by lead exposure from 2002 to 2016 of 19.7%, resulting in an overall reduction of age-standardized CKD DALY rates by 0.2%; this reduction contributed to 0.9% of the total change in age-standardized CKD DALY rates (Figure 2). Substantial increases were seen in CKD DALYs caused by metabolic risk factors, contributing to 93.8% of the total change in age-standardized CKD DALY rates. Increases in CKD DALYs for metabolic risk factors were seen in high fasting plasma glucose levels, with a 29.5% change from 2002 to 2016 contributing to a 9.3% increase in overall age-standardized CKD DALY rates; high body mass index, with a 30.9% change from 2002 to 2016 resulting in a 6.2% overall increase in age-standardized CKD DALY rates; and high systolic blood pressure, with a 10.1% change from 2002 to 2016 resulting in a 2.3% overall increase in age-standardized CKD DALY rates (Figure 2). There were also increases in CKD burden caused by dietary risks, which contributed to 5.3% of the total change in age-standardized CKD DALY rates. Increases in CKD burden for dietary risks were seen in a diet high in sodium, with a 21.7% change from 2002 to 2016 resulting in 0.1% increase in overall age-standardized DALY rates, and a diet high in sugar-sweetened beverages, with a 26.2% change from 2002 to 2016 contributing to a 0.9% increase in overall age-standardized DALY rates (Figure 2). Decomposition analyses of death showed consistent results and are provided in eFigure 4 in the Supplement.

#### Change in Age-Standardized DALY Rates by CKD Causes

Decomposition of age-standardized DALY rates in the United States by the 4 causes of CKD suggest that changes were primarily associated with increases in CKD due to diabetes, with a 21.8% change from 2002 to 2016 contributing to an overall 11.8% increase in age-standardized DALY rates, and CKD due to hypertension, with a 22.0% change from 2002 to 2016 contributing to an overall 4.0% increase in age-standardized DALY rates (eFigure 5 in the Supplement). The change in age-standardized DALY rate of CKD due to glomerulonephritis was 10.4%, leading to an overall 1.1% increase in age-standardized DALY rates, and change in age-standardized DALY rate of CKD due to other causes was 10.3%, leading to an overall 1.7% increase in age-standardized DALY rates (eFigure 5 in the Supplement). Decomposition analyses of death showed consistent results and are provided in eFigure 5 in the Supplement.

#### Changes in the Probability of Death Due to CKD From 2002 to 2016

**Figure 3.**
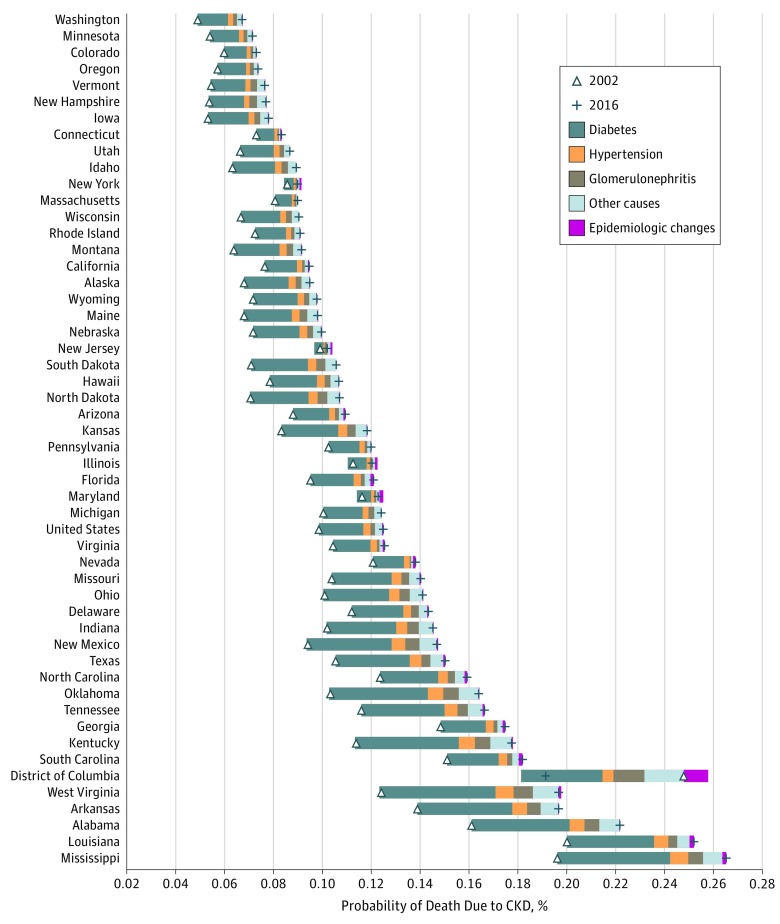
Change in Probability of Death Due to Chronic Kidney Disease (CKD) Among Individuals Aged 20 to 54 Years in the United States, 2002-2016 The triangle represents the probability of death due to CKD in 2002, and the plus sign marks 2016. The distance between these points is the change in probability of death due to CKD. The width of each colored bar represents the total change in probability of death due to CKD attributable to the corresponding factor. The size of the bar to the left of the triangle displays the amount of decrease in probability of death; the size to the right, the increase in probability. Rows are ordered by the probability of death due to CKD in 2016.

**Figure 4.**
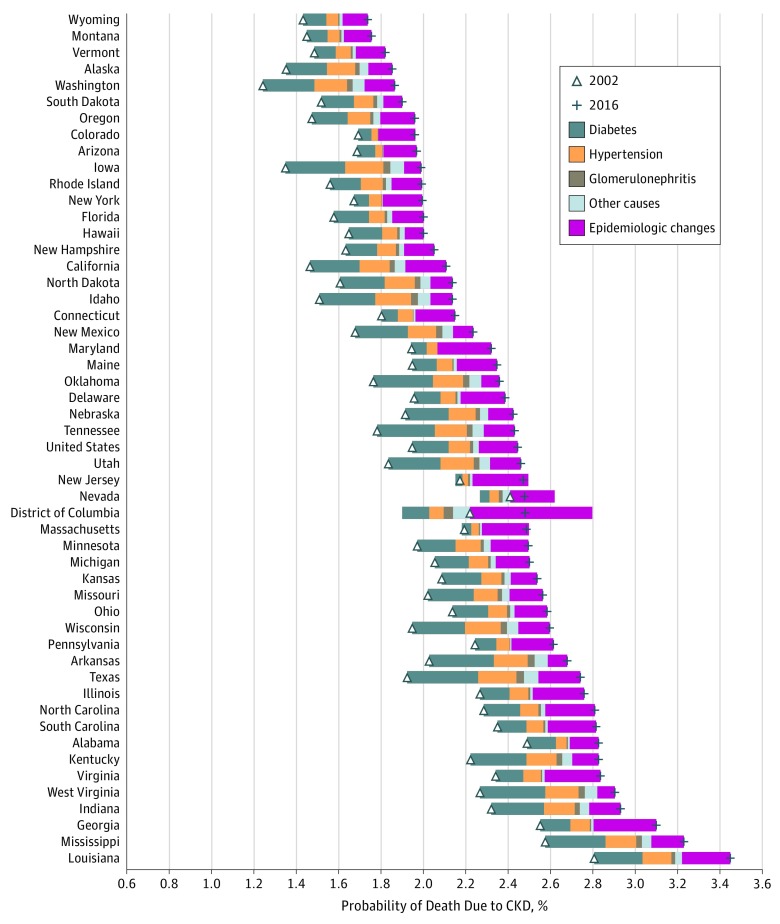
Change in Probability of Death Due to Chronic Kidney Disease (CKD) Among Individuals Aged 55 to 90 Years in the United States, 2002-2016 The triangle represents the probability of death due to CKD in 2002, and the plus sign marks 2016. The distance between these points is the change in probability of death due to CKD. The width of each colored bar represents the total change in probability of death due to CKD attributable to the corresponding factor. The size of the bar to the left of the triangle displays the amount of decrease in probability of death; the size to the right, the increase in probability. Rows are ordered by the probability of death due to CKD in 2016.

Changes were examined in the probability of death due to CKD from 2002 to 2016 in adults aged 20 to 54 years and older adults aged 55 to 89 years. From 2002 to 2016, among those aged 20 to 54 years, the probability of death due to CKD increased from 0.099% to 0.125% (26.8% increase); the increase in probability of death was largely associated with CKD due to diabetes (69.1%) (Figure 3). The magnitude of increase was variable across the United States and was evident in all geographical areas except the District of Columbia. Among those aged 55 to 89 years, the probability of death due to CKD increased from 1.95% to 2.45% (25.6% increase); the increase was associated with CKD due to diabetes (34.8%) and with epidemiologic changes reflecting a decrease in the underlying probability of death from competing causes other than CKD (37.2%) (Figure 4). The magnitude of increase in probability of death due to CKD was variable but evident among all geographical areas.

### CKD Burden and Sociodemographic Development

**Figure 5.**
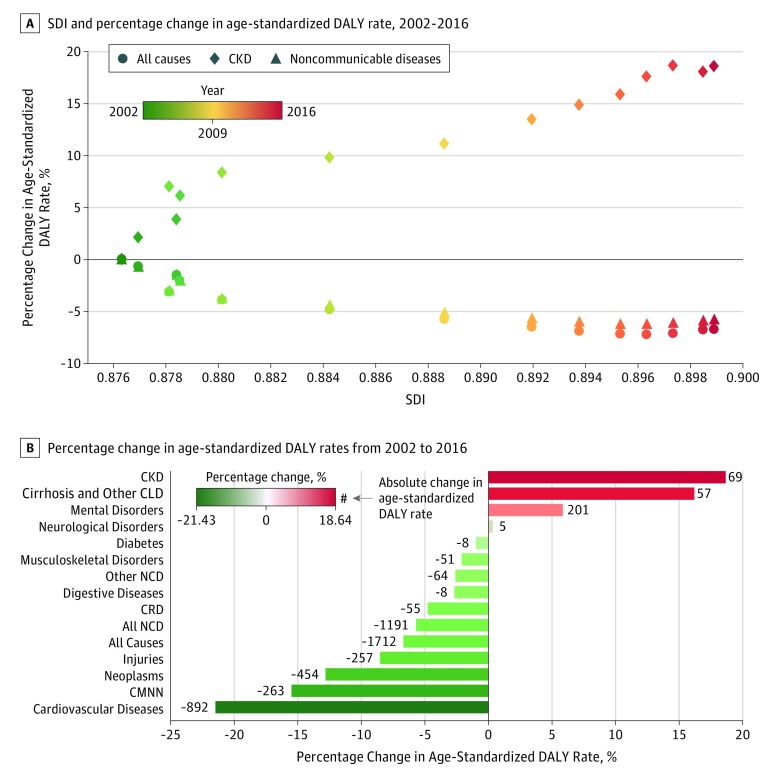
Comparative Assessment of Age-Standardized Disability-Adjusted Life Year (DALY) Rates Due to Chronic Kidney Disease (CKD) in the United States, 2002-2016 Sociodemographic index (SDI) is a summary measure of a geographical area’s income, education, and fertility rate. Values range from 0 to 1; a higher value indicates greater sociodemographic development. CLD indicates chronic liver diseases; CRD, chronic respiratory diseases; CMNN, communicable, maternal, neonatal, and nutritional diseases; and NCD, noncommunicable diseases.

From 2002 to 2016, the United States experienced an increase in sociodemographic development; during the same period, decreases in age-standardized DALY rates of all causes and noncommunicable disease causes were observed. Chronic kidney disease diverged from this trend in that as SDI increased, age-standardized DALY rate of CKD increased (Figure 5A). At the state level, as SDI increased, age-standardized CKD DALY rates increased in all states but not in the District of Columbia, which exhibited an increase in SDI and had the highest SDI in the United States (and globally) but showed a reduction in age-standardized CKD DALY rate (eFigure 6 in the Supplement).

Results of observed-to-expected ratios of age-standardized DALY rates in 2016 based on SDI for each geographical area are shown in eFigure 7 in the Supplement. There was heterogeneity in that several geographical areas exhibited a lower than expected burden, but several areas, including District of Columbia, Louisiana, Mississippi, Alabama, Georgia, West Virginia, Virginia, South Carolina, Maryland, Arkansas, Massachusetts, Delaware, and Illinois, exhibited a higher observed burden than expected on the basis of SDI. A frontier analysis of age-standardized DALY rates in 2016 delineated high-performing geographical areas based on their SDI and suggests that there were several areas, including all with observed-to-expected ratios more than 1 and several with observed-to-expected ratios less than 1, that were distant from the frontier, highlighting unrealized potential for reduction in CKD DALYs (eFigure 8 in the Supplement).

### CKD Burden in Context

To place the change in the burden of CKD in context, a comparative evaluation was performed of change in CKD burden vis-à-vis change in burden of other diseases. Although burden (measured as absolute and percent change in age-standardized DALY rates from 2002 to 2016) of all causes; communicable, maternal, neonatal, and nutritional diseases; and all noncommunicable diseases have decreased, the burden of CKD has increased (Figure 5B). Among level 2 GBD causes, although burden has decreased (from highest to lowest) for cardiovascular diseases, neoplasms, chronic respiratory diseases, digestive diseases, other noncommunicable diseases, musculoskeletal disorders, and diabetes, the burden has substantially increased (from highest to lowest) for CKD, and the increase outpaced the changes in burden of all level 2 causes, including cirrhosis and other chronic liver diseases, mental disorders, and neurological disorders (Figure 5B).

## Discussion

In this study, the change in burden of CKD from 2002 to 2016 was described at the state level. Our findings suggest a substantial increase in loss of health and in death due to CKD. In 2016, there were nearly 2 million healthy life-years lost owing to CKD (52.6% increase from 2002), and nearly 83 000 deaths due to CKD (58.3% increase from 2002). Our results suggest heterogeneity among US states; some states exhibited more than 2-fold higher rates of DALYs and deaths. The increase in burden of CKD was associated with an increase in exposure to metabolic and dietary risk factors, population growth, and aging, which manifested in a disproportionate increase in the burden of CKD due to diabetes and hypertension. From 2002 to 2016, the probability of death due to CKD increased among individuals in the 20- to 54-year age group and among those in the older than 55-year age group. This increase was primarily associated with CKD due to diabetes in the 20- to 54-year age group and a combination of factors in the older than 55-year age group, including CKD due to diabetes and decreasing probability of death from competing causes other than CKD. Over the past 15 years, the United States experienced improvement in sociodemographic development and a directionally concordant increase in CKD burden, and several states were observed to have higher age-standardized DALY rates than were expected based on SDI. Our frontier analyses suggest unrealized potential for improvement at all levels of the sociodemographic development spectrum. Considering the change in CKD burden in a broader context, the rate of increase in the US burden of CKD outpaced the changes in the US burden of other diseases.

The increase in the burden of CKD in the United States was greater compared with global epidemiologic trends of CKD^22^ and other diseases in the United States. Although the overall health in the United States improved from 2002 to 2016, as age-standardized DALY rates from all causes decreased from 25 687 per 100 000 population (95% UI, 22 557-28 631 per 100 000 population) to 23 975 per 100 000 population (95% UI, 21 014-27 323 per 100 000 population), rates of CKD increased from 371 per 100 000 population (95% UI, 336-406 per 100 000 population; accounting for 1.4% of all DALYs) to 440 per 100 000 population (95% UI, 395-485 per 100 000 population; accounting for 1.8% of all DALYs).^8,11^ From 2002 to 2016, the age-standardized DALY rate of noncommunicable disease decreased from 20 968 per 100 000 population (95% UI, 18 100-23 597 per 100 000 population) to 19 776 per 100 000 population (95% UI, 17 065-22 775 per 100 000 population). The increase in the burden of CKD DALYs now contributes to a larger share of overall noncommunicable disease burden, which increased from 1.8% in 2002 to 2.2% in 2016.^8,11^ Furthermore, the rate of increase in the age-standardized DALY rate of CKD outpaced that of all level 2 GBD causes. Divergence was observed because as the SDI increased, CKD burden increased, whereas burden of all-cause and noncommunicable disease DALYs decreased. Finally, the findings suggest that the increase in probability of death due to CKD among older adults was a manifestation of a decreased probability of death from causes other than CKD. Together, the findings suggest a new phase of epidemiologic transition in the United States that reflects a reduction in health loss and mortality from several noncommunicable diseases for which progress in the decline in burden has been made and a parallel increase in burden of other noncommunicable disease, including CKD. For CKD, the lack of progress and the sensitivity to increased exposure to metabolic and dietary risk factors, which may be linked to SDI, may also be leading to an accelerated pace of health loss.^4,23,24^

The decomposition analyses suggest that although aging and population growth may have contributed to the increasing burden of CKD, increased exposure to dietary and metabolic risk factors may also have been associated with the increase in the burden of CKD, which manifested primarily as CKD due to diabetes. The variability in CKD burden attributable to risk exposure among states suggests the need to examine potential policy levers or other precision-targeted population-wide mechanisms to influence or modulate the extent of exposure to these risk factors at the state level. The observed-to-expected ratios and frontier analyses revealed a potential for reduction in the burden of CKD at all levels of socioeconomic development.

From 1990 to 2016 in the United States, the probability of death decreased among young adults for most noncommunicable diseases.^1^ In this study, the probability of death due to CKD was increased among young adults, and this increase was in large part attributed to CKD due to diabetes. Studies have shown that risk of mortality due to CKD is greater among younger adults (and is attenuated with increasing age^25,26^). Earlier in life, CKD portends serious consequences, which manifest in a higher probability of death among the segment of the population that contributes considerably to economic prosperity, representing significant loss of human capital. Greater attention must be devoted at the local, state, and federal levels to mitigate the development of CKD, and earlier attention to its consequences among young adults is warranted.

The association between socioeconomic development and health outcomes is well established.^1,6,27,28,29^ Recent work from the GBD group showed that, as SDI in the United States increased, burden of cardiovascular disease decreased.^1,29^ However, this study showed that, as the SDI in the United States increased, burden of CKD also increased. This increase was also antithetical to change in all-cause and noncommunicable disease burden, which changed in a directionally discordant manner with SDI (as SDI increased, burden from all causes and noncommunicable diseases decreased). This finding may reflect the degree to which progress has been made in addressing the burden of cardiovascular disease (which shares several risk factors with CKD) and the relative stagnation in progress in addressing the burden of CKD. Furthermore, as suggested in the decomposition analyses, increased sociodemographic progress may have been associated with wider exposure to several dietary risk factors and more pronounced expression of metabolic risk factors, which may have also partially contributed to this trend of concordant change in SDI and disease burden.^4^ The increase in the burden of CKD should be reflected in the health priorities and resource allocation at the state and federal levels.

The District of Columbia was an outlier in many analyses; its SDI substantially increased from 2002 to 2016, when it was the highest in the world. There was also a reduction in DALYs in the District of Columbia owing to decreased risk exposure that corresponded with decreased probability of death among young adults. A reduction in other diseases, including cardiovascular disease, also occurred in the area.^1,29^ Significant demographic changes, including migration of sicker and economically disadvantaged individuals out of the area and migration of healthier and wealthier individuals into the area owing in part to the rapid rise in housing cost, may have been associated with the changes in disease burden.^1,29^

### Strengths and Limitations

 Key strengths include the use of GBD data, which facilitates a comparative assessment of all diseases and risk factors under the same computational framework. The development of decomposition analyses provided insight into demographic changes, risk factors, and causes of disease as contributors to change in the burden of CKD over the past 15 years. Probability of death analyses not only considered the changes in causes of CKD but also incorporated changes in the probability of death from causes other than CKD. The GBD also provides a quantitative measure of uncertainty (UI) to show how much is known and how much is uncertain about a specific measure of burden. Quantitating the burden of disease at the state level may be important because of the vital role of states in many aspects of health and social policy (eg, the Medicaid program, regulation of private insurers) and tax policies (eg, for sugar-sweetened beverages) and considering that individual states also have different demographic, social, and economic circumstances.^1,29^ Finally, a contextual evaluation was provided of the change in burden of CKD vis-à-vis changes in the epidemiologic features of other diseases in the United States.

This study has several limitations. We provided an assessment of the epidemiologic changes in CKD burden over 15 years at the state level; this macro-level assessment does not capture trends for smaller locales (eg, counties, zip codes) and within-state differences between urban and rural areas.^30^ This study used GBD data and methodologies to generate the estimates in this report, and although the data and methods are considered to be robust and reliable, they are limited by the quality of the available data.^14^ However, GBD uses an integrative metaregression approach that comprehensively incorporates all dimensions of health data, accounts for temporal and spatial differences and differences in data sources and biases, corrects for inconsistencies, and analyzes levels and trends for causes and risk factors within the same computational framework, which facilitates comparison across geographies, over time, and across disease categories.^10,14,31^ Attribution of CKD burden to a single cause does not account for CKD caused by dual conditions (eg, diabetes and hypertension). Modeling strategies of the GBD do not currently consider albuminuria and do not account for CKD of unknown cause (an emerging and important entity), and methods of cause attribution may result in overestimation of rapidly progressive CKD (eg, glomerulonephritis).^4,9,10,11^ Furthermore, because of the paucity of subnational data on the nonfatal burden of CKD, covariates for prevalence of diabetes and mean systolic blood pressure for each state were included in GBD models to help inform estimates in states where subnational data were not available.^1^ The change in probability of death was investigated for individuals 20 years or older. All other analyses, however, were not restricted to the adult population. Decomposition analyses included consideration of major demographic factors, the 6 risk factors for CKD (as defined by the GBD comparative risk assessment framework),^4^ and the 4 causes of CKD. Other variables, including race/ethnicity, are important^30,32,33,34^; however, they are not considered in the GBD framework currently. Subnational state-level data on health care access and quality are not yet available. However, although several limitations were outlined, GBD estimates continue to be updated. It is anticipated that the GBD framework and methodologies will evolve to address these limitations.

## Conclusions

Our study revealed that, from 2002 to 2016, the burden of CKD in the United States increased and was variable among states. This increase may be associated with increased risk exposure and demographic expansion leading to increased probability of death from CKD, especially among young adults. The findings suggest that an effort to target the reduction of CKD through greater attention to metabolic and dietary risks, especially among younger adults, is necessary.

## Notes

Supplement. **eAppendix.** Supplemental Methods **eReferences.** **eTable 1.** Terms and Definitions **eTable 2.** Number and Age-Standardized Rate for Overall DALY Due to CKD and by Cause, With Percentage Change From 2002 to 2016 for the United States and by State and District **eTable 3.** Number and Age-Standardized Rate for Overall Deaths Due to CKD and by Cause, With Percentage Change From 2002 to 2016 for the United States and by State and District **eTable 4.** Years Living With Disability (YLD) Due to CKD in the United States and by State and District in the Overall Population, and in Females and Males **eTable 5.** Years of Life Lost (YLL) Due to CKD in the United States and by State and District in the Overall Population, and in Females and Males **eTable 6.** Disability-Adjusted Life Years (DALYs) Due to CKD in the United States and by State and District in Females and Males **eTable 7.** Death Due to CKD in the United States and by State and District in Females and Males **eFigure 1.** Maps of the Age-Standardized Disability-Adjusted Life-Years (DALYs) Due to Chronic Kidney Disease in the United States in Females and Males **eFigure 2.** Maps of the Age-Standardized Death Due to Chronic Kidney Disease in the United States in Females and Males **eFigure 3.** Decomposition of the Percentage Change in Chronic Kidney Disease Burden in the United States From 2002 to 2016 Due to Changes in Age Structure, Size of the Population, and Risk Exposure **eFigure 4.** Decomposition of the Percentage Change in Age-Standardized Death Rates Due to Chronic Kidney Disease in the United States From 2002 to 2016 by Risk Factor **eFigure 5.** Decomposition of the Percentage Change in Age-Standardized CKD Burden Rate in the United States from 2002 to 2016 by Cause **eFigure 6.** Age-Standardized Disability-Adjusted Life-Year (DALY) Rates Due to Chronic Kidney Disease in 2002 vs 2016 by Sociodemographic Index (SDI) **eFigure 7.** Observed to Expected Ratio of Age-Standardized Disability-Adjusted Life-Year (DALY) Rate Due to CKD in 2016 Based on a State or District’s Sociodemographic Index (SDI) **eFigure 8.** Frontier Analysis for Age-Standardized Disability-Adjusted Life-Year (DALY) Rates Due to CKD by Sociodemographic Index (SDI) in 2016
