# Supplementary material for: Changes in the US Burden of Chronic Kidney Disease From 2002 to 2016: An Analysis of the Global Burden of Disease Study
Source: JAMA Netw Open. 2018 Nov 30;1(7):e184412. doi: 10.1001/jamanetworkopen.2018.4412 (PMC6324659; doi:10.1001/jamanetworkopen.2018.4412)
Supplement: Supplement. — eAppendix. Supplemental Methods eReferences. eTable 1. Terms and Definitions eTable 2. Number and Age-Standardized Rate for Overall DALY Due to CKD and by Cause, With Percentage Change From 2002 to 2016 for the United States and by State and District eTable 3. Number and Age-Standardized Rate for Overall Deaths Due to CKD and by Cause, With Percentage Change From 2002 to 2016 for the United States and by State and District eTable 4. Years Living With Disability (YLD) Due to CKD in the United States and by State and District in the Overall Population, and in Females and Males eTable 5. Years of Life Lost (YLL) Due to CKD in the United States and by State and District in the Overall Population, and in Females and Males eTable 6. Disability-Adjusted Life Years (DALYs) Due to CKD in the United States and by State and District in Females and Males eTable 7. Death Due to CKD in the United States and by State and District in Females and Males eFigure 1. Maps of the Age-Standardized Disability-Adjusted Life-Years (DALYs) Due to Chronic Kidney Disease in the United States in Females and Males eFigure 2. Maps of the Age-Standardized Death Due to Chronic Kidney Disease in the United States in Females and Males eFigure 3. Decomposition of the Percentage Change in Chronic Kidney Disease Burden in the United States From 2002 to 2016 Due to Changes in Age Structure, Size of the Population, and Risk Exposure eFigure 4. Decomposition of the Percentage Change in Age-Standardized Death Rates Due to Chronic Kidney Disease in the United States From 2002 to 2016 by Risk Factor eFigure 5. Decomposition of the Percentage Change in Age-Standardized CKD Burden Rate in the United States from 2002 to 2016 by Cause eFigure 6. Age-Standardized Disability-Adjusted Life-Year (DALY) Rates Due to Chronic Kidney Disease in 2002 vs 2016 by Sociodemographic Index (SDI) eFigure 7. Observed to Expected Ratio of Age-Standardized Disability-Adjusted Life-Year (DALY) Rate Due to CKD in 2016 Based on a State [file jamanetwopen-1-e184412-s001.pdf]

Supplementary Online Content

Bowe B, Xie Y, Li T, et al. Changes in the US burden of chronic kidney disease from 2002 to 2016: an analysis of the Global Burden of Disease study. *JAMA Netw Open*. 2018;1(7):e184412. doi:10.1001/jamanetworkopen.2018.4412

eAppendix. Supplemental Methods

eReferences.

eTable 1. Terms and Definitions

eTable 2. Number and Age-Standardized Rate for Overall DALY Due to CKD and by Cause, With Percentage Change From 2002 to 2016 for the United States and by State and District

eTable 3. Number and Age-Standardized Rate for Overall Deaths Due to CKD and by Cause, With Percentage Change From 2002 to 2016 for the United States and by State and District

eTable 4. Years Living With Disability (YLD) Due to CKD in the United States and by State and District in the Overall Population, and in Females and Males

eTable 5. Years of Life Lost (YLL) Due to CKD in the United States and by State and District in the Overall Population, and in Females and Males

eTable 6. Disability-Adjusted Life Years (DALYs) Due to CKD in the United States and by State and District in Females and Males

eTable 7. Death Due to CKD in the United States and by State and District in Females and Males

eFigure 1. Maps of the Age-Standardized Disability-Adjusted Life-Years (DALYs) Due to Chronic Kidney Disease in the United States in Females and Males

eFigure 2. Maps of the Age-Standardized Death Due to Chronic Kidney Disease in the United States in Females and Males

eFigure 3. Decomposition of the Percentage Change in Chronic Kidney Disease Burden in the United States From 2002 to 2016 Due to Changes in Age Structure, Size of the Population, and Risk Exposure

eFigure 4. Decomposition of the Percentage Change in Age-Standardized Death Rates Due to Chronic Kidney Disease in the United States From 2002 to 2016 by Risk Factor

eFigure 5. Decomposition of the Percentage Change in Age-Standardized CKD Burden Rate in the United States from 2002 to 2016 by Cause

eFigure 6. Age-Standardized Disability-Adjusted Life-Year (DALY) Rates Due to Chronic Kidney Disease in 2002 vs 2016 by Sociodemographic Index (SDI)

eFigure 7. Observed to Expected Ratio of Age-Standardized Disability-Adjusted Life-Year (DALY) Rate Due to CKD in 2016 Based on a State or District's Sociodemographic Index (SDI)

eFigure 8. Frontier Analysis for Age-Standardized Disability-Adjusted Life-Year (DALY) Rates Due to CKD by Sociodemographic Index (SDI) in 2016

This supplementary material has been provided by the authors to give readers additional information about their work.

eAppendix. Supplemental Methods

GBD estimates of CKD burden:

GBD estimates of burden are updated each iteration based on updated data sources and methodologies. United States data sources used in fatal and nonfatal estimation of CKD are available from the GBD Input Sources Tool[1]. CKD was defined as eGFR <60 ml/min/1.73m[2]. Sources used to estimate CKD burden were identified through a PubMed search selecting titles and abstracts containing “chronic kidney disease” and “prevalen*” with a date since Jan 1, 2015 that did not have a subject heading that denoted an only animal studies. Identified sources were combined with those identified from searchers in prior years, such that the total literature body covered from 1980 to 2016. Sources were supplemented with population-based surveys that measured renal function. A list of sources is included below for fatal and non-fatal estimates.

Search string (run on PubMed):
((((("chronic kidney disease"[Title/Abstract]) AND prevalen*[Title/Abstract]) AND ("2015/1/1"[Date - Publication] : "3000"[Date - Publication])) NOT ((animals[MeSH] NOT humans[MeSH]))))

For cause of death estimates, data from the United States Renal Data System Annual Data Report and United States National Vital Statistics System Custom Mortality Data and United States Military Deaths were used. The underlying cause of death data were mapped based on GBD cause of death ICD mapping methods; garbage codes, which are causes of death which should not be an underlying cause of death, were redistributed to other codes based on methods including regression models and fractional reassignment of a death assigned multiple causes. Cause of death from CKD was attributed using the following ICD-10 and ICD-9 codes:

© Bowe B et al. *JAMA Network Open*

ICD revision	Codes
ICD-10	D63.1, E10.2, E11.2, E12.2, E13.2, E14.2, I12-I13.9, N02-N08.8, N15.0, N18-N18.9, Q61-Q62.8
ICD-9	250.4, 403-404.9, 581-583.9, 585-585.9, 589-589.9, 753-753.3

Data were then used in cause of death ensemble method (CODEm) models to estimate death rate due to CKD. The CODEm model is an ensemble model which takes into account multiple covariate combinations and model types to enhance the accuracy of prediction. Cause of Death Correct (CoDCorrect) is then applied, along with estimates of other causes of death, to reconcile differences between the causes of death and overall mortality. Sub-causes of death due to CKD were inform by end-stage renal disease registries. Data from the Australia & New Zealand Dialysis and Transplant Registry, that contains age and subcause-specific data, were used in DisMod-MR 2.1 models (an integrative metaregression approach) that adjusted for diabetes prevalence and mean systolic blood pressure to obtain estimates of proportions for each subtype by location, year, age, and sex. The estimated proportions are adjusted so that they sum to 1, and then applied to the parent CKD CODEm model to estimate the death rate for each sub cause of death.

For nonfatal estimates in GBD 2016 a total of 23 distinct data sources including United States National Health and Nutrition Examination Survey, United States Renal Data System Annual Data Report and many other national or subnational studies were identified (a complete list provided below). DisMod-MR 2.1, a metaregression method that evaluates all available information including hospital inpatient and outpatient data, claims data, and other population study datasets on diseases that passes a minimum quality standard were used to estimate the prevalence and incidence by cause and sequalae[2,3]. Disability weights were than applied to calculate the YLDs[4].

© Bowe B et al. *JAMA Network Open*

Risk factors for CKD were defined based on the comparative risk assessment conceptual framework, where risks of CKD were hierarchically organized, and quantification of their contribution could be conducted at any level of the framework. The risk-outcome pairs were included based on evidence rules and had to have met World Cancer Research Fund (WCRF) criteria for convincing or probable evidence. The overall modeling strategy, and risk-outcome pairs are then reviewed by an independent advisory council (IAC). The exposure levels of risk factors were estimated, standardized and used in DisMod-MR 2.1 meta regression to estimate the burden of CKD due to each risk factors.

All GBD data are publicly available from the Global Health Data Exchange (http://ghdx.healthdata.org/ihme_data),

Data sources for cause of death:

United States Renal Data System Coordinating Center. USRDS 2010 Annual Data Report: Atlas of Chronic Kidney Disease and End-Stage Renal Disease in the United States. Bethesda, United States: National Institute of Diabetes and Digestive and Kidney Diseases (NIDDK), National Institutes of Health, 2010
United States Renal Data System Coordinating Center. USRDS 2005 Annual Data Report: Atlas of End-Stage Renal Disease in the United States. Bethesda, United States: National Institute of Diabetes and Digestive and Kidney Diseases (NIDDK), National Institutes of Health, 2005
United States Renal Data System Coordinating Center. USRDS 2013 Annual Data Report: Atlas of Chronic Kidney Disease and End-Stage Renal Disease in the United States. Bethesda, United States: National Institute of Diabetes and Digestive and Kidney Diseases (NIDDK), National Institutes of Health, 2013
United States Renal Data System Coordinating Center. USRDS 2000 Annual Data Report: Atlas of End-Stage Renal Disease in the United States. Bethesda, United States: National Institute of Diabetes and Digestive and Kidney Diseases (NIDDK), National Institutes of Health, 2000
U.S. Renal Data System. USRDS 1996 Annual Data Report. Bethesda (MD), United States: National Institute of Diabetes and Digestive and Kidney Diseases (NIDDK), National Institutes of Health, 1996
U.S. Renal Data System. USRDS 1994 Annual Data Report. Bethesda (MD), United States: National Institute of Diabetes and Digestive and Kidney Diseases (NIDDK), National Institutes of Health, 1994
National Center for Health Statistics, Centers for Disease Control and Prevention. United States NVSS Mortality Data 1994 - NBER. Hyattsville, United States: National Center for Health Statistics, Centers for Disease Control and Prevention

National Center for Health Statistics, Centers for Disease Control and Prevention. United States NVSS Mortality Data 1995 - NBER. Hyattsville, United States: National Center for Health Statistics, Centers for Disease Control and Prevention
National Center for Health Statistics, Centers for Disease Control and Prevention. United States NVSS Mortality Data 1996 - NBER. Hyattsville, United States: National Center for Health Statistics, Centers for Disease Control and Prevention
National Center for Health Statistics, Centers for Disease Control and Prevention. United States NVSS Mortality Data 1997 - NBER. Hyattsville, United States: National Center for Health Statistics, Centers for Disease Control and Prevention
National Center for Health Statistics, Centers for Disease Control and Prevention. United States NVSS Mortality Data 1998 - NBER. Hyattsville, United States: National Center for Health Statistics, Centers for Disease Control and Prevention
National Center for Health Statistics, Centers for Disease Control and Prevention. United States NVSS Mortality Data 1999 - NBER. Hyattsville, United States: National Center for Health Statistics, Centers for Disease Control and Prevention
National Center for Health Statistics, Centers for Disease Control and Prevention. United States NVSS Mortality Data 2000 - NBER. Hyattsville, United States: National Center for Health Statistics, Centers for Disease Control and Prevention
National Center for Health Statistics, Centers for Disease Control and Prevention. United States NVSS Mortality Data 2001 - NBER. Hyattsville, United States: National Center for Health Statistics, Centers for Disease Control and Prevention
National Center for Health Statistics, Centers for Disease Control and Prevention. United States NVSS Mortality Data 2002 - NBER. Hyattsville, United States: National Center for Health Statistics, Centers for Disease Control and Prevention
National Center for Health Statistics, Centers for Disease Control and Prevention. United States NVSS Mortality Data 2003 - NBER. Hyattsville, United States: National Center for Health Statistics, Centers for Disease Control and Prevention
National Center for Health Statistics, Centers for Disease Control and Prevention. United States NVSS Mortality Data 2004 - NBER. Hyattsville, United States: National Center for Health Statistics, Centers for Disease Control and Prevention
National Center for Health Statistics, Centers for Disease Control and Prevention. United States NVSS Custom Mortality Data 2005. Hyattsville, United States: National Center for Health Statistics, Centers for Disease Control and Prevention
National Center for Health Statistics, Centers for Disease Control and Prevention. United States NVSS Custom Mortality Data 2006. Hyattsville, United States: National Center for Health Statistics, Centers for Disease Control and Prevention
National Center for Health Statistics, Centers for Disease Control and Prevention. United States NVSS Custom Mortality Data 2007. Hyattsville, United States: National Center for Health Statistics, Centers for Disease Control and Prevention
National Center for Health Statistics, Centers for Disease Control and Prevention. United States NVSS Custom Mortality Data 2008. Hyattsville, United States: National Center for Health Statistics, Centers for Disease Control and Prevention
National Center for Health Statistics, Centers for Disease Control and Prevention. United States NVSS Custom Mortality Data 2009. Hyattsville, United States: National Center for Health Statistics, Centers for Disease Control and Prevention
National Center for Health Statistics, Centers for Disease Control and Prevention. United States NVSS Custom Mortality Data 2010. Hyattsville, United States: National Center for Health Statistics, Centers for Disease Control and Prevention

National Center for Health Statistics, Centers for Disease Control and Prevention. United States NVSS Custom Mortality Data 2011. Hyattsville, United States: National Center for Health Statistics, Centers for Disease Control and Prevention
National Center for Health Statistics, Centers for Disease Control and Prevention. United States NVSS Custom Mortality Data 2012. Hyattsville, United States: National Center for Health Statistics, Centers for Disease Control and Prevention
National Center for Health Statistics (NCHS), Centers for Disease Control and Prevention (CDC), U.S. Department of Defense. United States NVSS Mortality Data 1980 - NBER and United States Military Deaths 1980-2014
National Center for Health Statistics (NCHS), Centers for Disease Control and Prevention (CDC), U.S. Department of Defense. United States NVSS Mortality Data 1981 - NBER and United States Military Deaths 1980-2014
National Center for Health Statistics (NCHS), Centers for Disease Control and Prevention (CDC), U.S. Department of Defense. United States NVSS Mortality Data 1982 - NBER and United States Military Deaths 1980-2014
National Center for Health Statistics (NCHS), Centers for Disease Control and Prevention (CDC), U.S. Department of Defense. United States NVSS Mortality Data 1983 - NBER and United States Military Deaths 1980-2014
National Center for Health Statistics (NCHS), Centers for Disease Control and Prevention (CDC), U.S. Department of Defense. United States NVSS Mortality Data 1984 - NBER and United States Military Deaths 1980-2014
National Center for Health Statistics (NCHS), Centers for Disease Control and Prevention (CDC), U.S. Department of Defense. United States NVSS Mortality Data 1985 - NBER and United States Military Deaths 1980-2014
National Center for Health Statistics (NCHS), Centers for Disease Control and Prevention (CDC), U.S. Department of Defense. United States NVSS Mortality Data 1986 - NBER and United States Military Deaths 1980-2014
National Center for Health Statistics (NCHS), Centers for Disease Control and Prevention (CDC), U.S. Department of Defense. United States NVSS Mortality Data 1987 - NBER and United States Military Deaths 1980-2014
National Center for Health Statistics (NCHS), Centers for Disease Control and Prevention (CDC), U.S. Department of Defense. United States NVSS Mortality Data 1988 - NBER and United States Military Deaths 1980-2014
National Center for Health Statistics (NCHS), Centers for Disease Control and Prevention (CDC), U.S. Department of Defense. United States NVSS Mortality Data 1989 - NBER and United States Military Deaths 1980-2014
National Center for Health Statistics (NCHS), Centers for Disease Control and Prevention (CDC), U.S. Department of Defense. United States NVSS Mortality Data 1990 - NBER and United States Military Deaths 1980-2014
National Center for Health Statistics (NCHS), Centers for Disease Control and Prevention (CDC), U.S. Department of Defense. United States NVSS Mortality Data 1991 - NBER and United States Military Deaths 1980-2014
National Center for Health Statistics (NCHS), Centers for Disease Control and Prevention (CDC), U.S. Department of Defense. United States NVSS Mortality Data 1992 - NBER and United States Military Deaths 1980-2014
National Center for Health Statistics (NCHS), Centers for Disease Control and Prevention (CDC), U.S. Department of Defense. United States NVSS Mortality Data 1993 - NBER and United States Military Deaths 1980-2014

© Bowe B et al. *JAMA Network Open*

National Center for Health Statistics (NCHS), Centers for Disease Control and Prevention (CDC), U.S. Department of Defense. United States NVSS Mortality Data 1994 - NBER and United States Military Deaths 1980-2014
National Center for Health Statistics (NCHS), Centers for Disease Control and Prevention (CDC), U.S. Department of Defense. United States NVSS Mortality Data 1995 - NBER and United States Military Deaths 1980-2014
National Center for Health Statistics (NCHS), Centers for Disease Control and Prevention (CDC), U.S. Department of Defense. United States NVSS Mortality Data 1996 - NBER and United States Military Deaths 1980-2014
National Center for Health Statistics (NCHS), Centers for Disease Control and Prevention (CDC), U.S. Department of Defense. United States NVSS Mortality Data 1997 - NBER and United States Military Deaths 1980-2014
National Center for Health Statistics (NCHS), Centers for Disease Control and Prevention (CDC), U.S. Department of Defense. United States NVSS Mortality Data 1998 - NBER and United States Military Deaths 1980-2014
National Center for Health Statistics (NCHS), Centers for Disease Control and Prevention (CDC), U.S. Department of Defense. United States NVSS Mortality Data 1999 - NBER and United States Military Deaths 1980-2014
National Center for Health Statistics (NCHS), Centers for Disease Control and Prevention (CDC), U.S. Department of Defense. United States NVSS Mortality Data 2000 - NBER and United States Military Deaths 1980-2014
National Center for Health Statistics (NCHS), Centers for Disease Control and Prevention (CDC), U.S. Department of Defense. United States NVSS Mortality Data 2001 - NBER and United States Military Deaths 1980-2014
National Center for Health Statistics (NCHS), Centers for Disease Control and Prevention (CDC), U.S. Department of Defense. United States NVSS Mortality Data 2002 - NBER and United States Military Deaths 1980-2014
National Center for Health Statistics (NCHS), Centers for Disease Control and Prevention (CDC), U.S. Department of Defense. United States NVSS Mortality Data 2003 - NBER and United States Military Deaths 1980-2014
National Center for Health Statistics (NCHS), Centers for Disease Control and Prevention (CDC), U.S. Department of Defense. United States NVSS Mortality Data 2004 - NBER and United States Military Deaths 1980-2014
National Center for Health Statistics (NCHS), Centers for Disease Control and Prevention (CDC), U.S. Department of Defense. United States NVSS Custom Mortality Data 2005 and United States Military Deaths 1980-2014
National Center for Health Statistics (NCHS), Centers for Disease Control and Prevention (CDC), U.S. Department of Defense. United States NVSS Custom Mortality Data 2006 and United States Military Deaths 1980-2014
National Center for Health Statistics (NCHS), Centers for Disease Control and Prevention (CDC), U.S. Department of Defense. United States NVSS Custom Mortality Data 2007 and United States Military Deaths 1980-2014
National Center for Health Statistics (NCHS), Centers for Disease Control and Prevention (CDC), U.S. Department of Defense. United States NVSS Custom Mortality Data 2008 and United States Military Deaths 1980-2014
National Center for Health Statistics (NCHS), Centers for Disease Control and Prevention (CDC), U.S. Department of Defense. United States NVSS Custom Mortality Data 2009 and United States Military Deaths 1980-2014

National Center for Health Statistics (NCHS), Centers for Disease Control and Prevention (CDC), U.S. Department of Defense. United States NVSS Custom Mortality Data 2010 and United States Military Deaths 1980-2014
National Center for Health Statistics (NCHS), Centers for Disease Control and Prevention (CDC), U.S. Department of Defense. United States NVSS Custom Mortality Data 2011 and United States Military Deaths 1980-2014
National Center for Health Statistics (NCHS), Centers for Disease Control and Prevention (CDC), U.S. Department of Defense. United States NVSS Custom Mortality Data 2012 and United States Military Deaths 1980-2014
National Center for Health Statistics (NCHS), Centers for Disease Control and Prevention (CDC), U.S. Department of Defense. United States NVSS Custom Mortality Data 2013 and United States Military Deaths 1980-2014
National Center for Health Statistics (NCHS), Centers for Disease Control and Prevention (CDC), U.S. Department of Defense. United States NVSS Custom Mortality Data 2014 and United States Military Deaths 1980-2014

Data sources for non-fatal estimates

National Center for Health Statistics (NCHS), Centers for Disease Control and Prevention (CDC). United States National Health and Nutrition Examination Survey 2007-2008. Hyattsville, United States: National Center for Health Statistics (NCHS), Centers for Disease Control and Prevention (CDC), 2009
National Center for Health Statistics (NCHS), Centers for Disease Control and Prevention (CDC). United States National Health and Nutrition Examination Survey 2005-2006. Hyattsville, United States: National Center for Health Statistics (NCHS), Centers for Disease Control and Prevention (CDC), 2007
National Center for Health Statistics (NCHS), Centers for Disease Control and Prevention (CDC). United States National Health and Nutrition Examination Survey 2003-2004. Hyattsville, United States: National Center for Health Statistics (NCHS), Centers for Disease Control and Prevention (CDC)
National Center for Health Statistics (NCHS), Centers for Disease Control and Prevention (CDC). United States National Health and Nutrition Examination Survey 2009-2010. Hyattsville, United States: National Center for Health Statistics (NCHS), Centers for Disease Control and Prevention (CDC), 2011
National Center for Health Statistics (NCHS), Centers for Disease Control and Prevention (CDC). United States National Health and Nutrition Examination Survey 1988-1994. Hyattsville, United States: National Center for Health Statistics (NCHS), Centers for Disease Control and Prevention (CDC)
National Center for Health Statistics (NCHS), Centers for Disease Control and Prevention (CDC). United States National Health and Nutrition Examination Survey 2001-2002. Hyattsville, United States: National Center for Health Statistics (NCHS), Centers for Disease Control and Prevention (CDC)
National Center for Health Statistics (NCHS), Centers for Disease Control and Prevention (CDC). United States National Health and Nutrition Examination Survey 1999-2000. Hyattsville, United States: National Center for Health Statistics (NCHS), Centers for Disease Control and Prevention (CDC)
National Center for Health Statistics (NCHS) Centers for Disease Control and Prevention (CDC). United States National Health and Nutrition Examination Survey 2011-2012. Hyattsville, United States: National Center for Health Statistics (NCHS) Centers for Disease Control and Prevention (CDC), 2013

United States Renal Data System Coordinating Center. USRDS 2010 Annual Data Report: Atlas of Chronic Kidney Disease and End-Stage Renal Disease in the United States. Bethesda, United States: National Institute of Diabetes and Digestive and Kidney Diseases (NIDDK), National Institutes of Health, 2010
United States Renal Data System Coordinating Center. USRDS 2005 Annual Data Report: Atlas of End-Stage Renal Disease in the United States. Bethesda, United States: National Institute of Diabetes and Digestive and Kidney Diseases (NIDDK), National Institutes of Health, 2005
United States Renal Data System Coordinating Center. USRDS 2013 Annual Data Report: Atlas of Chronic Kidney Disease and End-Stage Renal Disease in the United States. Bethesda, United States: National Institute of Diabetes and Digestive and Kidney Diseases (NIDDK), National Institutes of Health, 2013
United States Renal Data System Coordinating Center. USRDS 2000 Annual Data Report: Atlas of End-Stage Renal Disease in the United States. Bethesda, United States: National Institute of Diabetes and Digestive and Kidney Diseases (NIDDK), National Institutes of Health, 2000
U.S. Renal Data System. USRDS 1996 Annual Data Report. Bethesda (MD), United States: National Institute of Diabetes and Digestive and Kidney Diseases (NIDDK), National Institutes of Health, 1996
U.S. Renal Data System. USRDS 1994 Annual Data Report. Bethesda (MD), United States: National Institute of Diabetes and Digestive and Kidney Diseases (NIDDK), National Institutes of Health, 1994
National Center for Health Statistics (NCHS), Centers for Disease Control and Prevention (CDC). United States National Health and Nutrition Examination Survey 2013-2014. Hyattsville, United States: National Center for Health Statistics (NCHS), Centers for Disease Control and Prevention (CDC)
United States Renal Data System. 2015 USRDS Annual Data Report: Epidemiology of Kidney Disease in the United States. National Institutes of Health, National Institute of Diabetes and Digestive and Kidney Diseases, Bethesda, MD, 2015
Arora P, Elkin PL, Eberle J, Bono JJ, Argauer L, Murray BM, Ram R, Venuto RC. An observational study of the quality of care for chronic kidney disease: a Buffalo and Albany, New York metropolitan area study. BMC Nephrol. 2015; 16: 199
Coresh J, Astor BC, Matsushita K, Grams M, Sang Y, Chronic Kidney Disease Prognosis Consortium (CKD-PC). United States Atherosclerosis Risk in Communities (ARIC) Study Chronic Kidney Disease Prevalence Data 1996-1998 [Unpublished]
Fox C, Hwang SJ, Meigs J, Upadhyay A, Chronic Kidney Disease Prognosis Consortium (CKD-PC). United States Framingham Heart Study Chronic Kidney Disease Prevalence Data 1995-1998 [Unpublished]
Jassal SK, Bergstrom J, Ix JH, Barrett-Connor E, Chronic Kidney Disease Prognosis Consortium (CKD-PC). United States - Rancho Bernardo Study Chronic Kidney Diseases Prevalence Data 1972-1974 [Unpublished]
Shlipak M, Sarnak MJ, Katz R, Fried LF, Chronic Kidney Disease Prognosis Consortium (CKD-PC). United States Cardiovascular Health Study (CHS) Chronic Kidney Disease Prevalence Data 2004 [Unpublished]
Shlipak M, Sarnak M, Katz R, Peralta C, Chronic Kidney Disease Prognosis Consortium (CKD-PC). United States Multi-Ethnic Study of Atherosclerosis (MESA) Chronic Kidney Disease Prevalence Data 2000 [Unpublished]
Organ Procurement and Transplantation Network (OPTN) (United States), U.S. Department of Health and Human Services, United Network for Organ Sharing (United States). United States Organ Procurement and Transplantation Network Data Report Advanced Build -

Transplant. Richmond, United States: Organ Procurement and Transplantation Network (OPTN) (United States)

Decomposition analysis:

The decomposition methodology of Das Gupta[5-7] was used to better understand the drivers of change from 2002 to 2016 in DALY and Death numbers. We decomposed the change of DALY and Death numbers by population size, age structure and risk exposure. The DALY or death numbers were considered to follow the formula:

$$DV_{ay, py, ey} = \sum_{i=1}^{21}(a_{i, y} * p_{y} * e_{i, y})$$

Where DV ay, py, ey represents the dependent variable (DALY or Death numbers) based on the factors of population size, age structure and risk exposure in year y. $a_{i\ y}$ represents the proportion of population for the age category i (amongst the21 age categories) in year y, where ages under 1, above 95, and every 5 years in between were considered a category. p_{y} represents the total population in given year y; and $e_{i, y}$ represents DALY rate or Death rate given age category i in year y. Years y were 2002 or 2016. The 3 factor decomposition analyses as described by Das Gupta[5-7], with number as a function of factors were then applied.

The age standardized DALY rates and Death rates were decomposed based on CKD risk factors as defined in the GBD comparative risk assessment framework which includes metabolic (high fasting plasma glucose, high systolic blood pressure, high body mass index), dietary (diet high in sodium, and diet high in sugar sweetened beverages), and environmental risk factors (lead exposure). GBD data for risk factors is not available every year; here we chose data from 2000 (the year prior and most proximal to 2002 with risk data), and applied the distribution of age-standardized risk factor attributable CKD DALY and death rates to corresponding 2002 rates. The GBD considers these risk factors to contribute to 100% of the estimated CKD DALY and Death rates. However, population attributable fractions often overlap and the sum of all risk

© Bowe B et al. *JAMA Network Open*

factors can overestimate DALY and Death rates. In order to keep the decomposition consistent with overall DALY rates and Death rates, risk factors were scaled by a factor of 1/sum (risk factors) so that they summed to be 100% of the estimated values while. This assumes that their proportional contribution to the DALY rate and death rate before and after scaling is fixed. Age-standardized rates were computed based on Rate=$\sum_{q=1}^{6} Rq\, y$. Where q represents one of the six risk factor and y represent the year and Rqy represent the age standardized rate for specific risk factor in given year y. Hence the change of rate=$\sum_{q=1}^{6}(Rq\, 2016 - Rq\, 2002)$.

We additionally decomposed the age standardized DALY and Death rates due to CKD based on CKD caused by diabetes, hypertension, glomerulonephritis and other causes. Similar to the decomposition by risk, Rate=$\sum_{q=1}^{4} Cq\, y$ and change of rate=$\sum_{q=1}^{4}(Cq\, 2016 - Cq\, 2002)$ where Cqy represent the age standardized rate for specific cause q in given year y.

The results of decomposition analyses interpreted as the effect of the change in one factor on DALY or Death numbers (or rates where applicable) while the other factors are held constant. All decomposition results are reported as percentage change from the relevant total value in 2002.

Probability of Death

Utilizing GBD data on overall death rates and rates of death due to CKD by cause, an abridged multiple decrement period life table was constructed [8,9]. The Chiang formula was then used for estimation of within interval probability of death, where deaths were assumed to occur in a uniform distribution throughout each age group[10]. For a given cause, the probability of death due to a specific cause for a given age group is:

© Bowe B et al. *JAMA Network Open*

$$_nq_x = \frac{n_nm_x}{1 + n * (1 - {}_na_x) * {}_nm_x}$$

Where q is the probability of dying, m the death rate cause, x the age at the start of the interval, n the width of the interval, and a proportion the proportion of the interval lived by those who die in the interval (for uniform distribution a is 0.5). Cumulative probability of death is then calculated using standard life table methods while accounting for competing risks of death. The decomposition method of Das Gupta was then applied to decompose the overall change in probability of CKD death due to diabetes mellitus, hypertension, glomerulonephritis, other causes, and as a result of change in probability of death from other competing, non-CKD, causes, which we refer as epidemiologic changes [5-7]. The rate of epidemiologic change was calculated by subtracting CKD rates from overall rates.

Observed vs. Expected analysis

Generalized estimation equation (GEE) models with an autoregressive covariance structure using data from 2002 to 2016 were built to establish relation between SDI and a state's age-standardized DALY rate and death rates due to CKD, with predictors of SDI, year, and an interaction between SDI and year. The year term provides an overall trend for the outcome as time has changed, and the interaction allows for the relation between SDI and outcome to vary in different years. Restricted cubic splines to allow for non-linear trends did not improve model fit, as assessed by BIC, so were not included. A GEE modeling approach was taken to estimate the marginal expected value of age-standardized DALY or death rate due to CKD given a states SDI and the year, while accounting for the correlation that results from repeated measurements in the same state over time. The ratios between estimated value and observed value at 2016 from the GBD study were than computed for each state and distract to indicate the difference between observed and expected value based on SDI and year.

Frontier Analysis

A frontier analysis was conducted using SDI and age-standardized DALY rate due to CKD in 2016 for all states and distract. A data envelope analysis utilizing the free disposal hull method was developed, which was chosen over the standard stochastic frontier as it allows for a non-linear frontier[11-13]. One thousand bootstrapped samples of the data were used to account for uncertainty in DALY estimates; to exclude influence of outliers, super-efficient countries were excluded in each bootstrapped sample[11]. The mean frontier at each SDI value from the bootstrapped samples was computed and placed in a LOESS regression to develop a smoothed frontier[11]. To understand the relationship of age-standardized CKD DALY rates vis-à-vis the frontier, we calculated the effective difference (the absolute distance from the frontier). States or district that achieved DALY rates lower than the frontiers were assigned a zero distance.

The DALY frontier pinpoints the minimum age-standardized DALY rate that could be attained for every state or district given its SDI. Distance from the frontier is termed effective difference; a large effective difference from the frontier suggests there may be unrealized opportunities for gains or improvement (reduction in CKD DALYs) that should be possible based on the state or district's place on the development spectrum.

All GBD data and codes are publicly available from the Global health data exchange (http://ghdx.healthdata.org/gbd-2016-code). Analytic code for this study may be obtained by request.

© Bowe B et al. *JAMA Network Open*

eReferences:

1. Global Burden of Disease Study 2016 (GBD 2016) Data Input Sources Tool. Institute for Health Metrics and Evaluation; 2016. ghdx.healthdata.org/gbd-2016/data-input-sources. Accessed 6/13/2018.
2. Collaborators USBoD, Mokdad AH, Ballestros K, et al. The State of US Health, 1990-2016: Burden of Diseases, Injuries, and Risk Factors Among US States. *Jama.* Apr 10 2018;319(14):1444-1472.
3. Abraham D. Flaxman TV, Christopher J.L. Murray. *An Integrative Metaregression Framework for Descriptive Epidemiology.* First Edition ed: University of Washington Press; 2015.
4. Disease GBD, Injury I, Prevalence C. Global, regional, and national incidence, prevalence, and years lived with disability for 328 diseases and injuries for 195 countries, 1990-2016: a systematic analysis for the Global Burden of Disease Study 2016. *Lancet.* Sep 16 2017;390(10100):1211-1259.
5. P. DG. *Standardization and decomposition of rates: a user's manual, Pages 19-36.* 1993.
6. Das Gupta P. Standardization and decomposition of rates from cross-classified data. *Genus.* Jul-Dec 1994;50(3-4):171-196.
7. Chevan A, Sutherland M. Revisiting Das Gupta: refinement and extension of standardization and decomposition. *Demography.* Aug 2009;46(3):429-449.
8. Carey JR. The multiple decrement life table: a unifying framework for cause-of-death analysis in ecology. *Oecologia.* 1989;78(1):131-137.
9. Chiang CL, Organization WH. Life table and mortality analysis. 1979.
10. Chiang CL. The life table and its applications. 1984.
11. Access GBDH, Quality Collaborators. Electronic address cue, Access GBDH, Quality C. Healthcare Access and Quality Index based on mortality from causes amenable to personal health care in 195 countries and territories, 1990-2015: a novel analysis from the Global Burden of Disease Study 2015. *Lancet.* May 18 2017.
12. Bogetoft PaO, L. *Benchmarking with data envelopment analysis, stochastic frontier analysis, and R. .* 2011 edition. ed2013.
13. Xie Y, Bowe B, Xian H, Balasubramanian S, Al-Aly Z. Rate of Kidney Function Decline and Risk of Hospitalizations in Stage 3A CKD. *Clinical journal of the American Society of Nephrology : CJASN.* Nov 6 2015;10(11):1946-1955.

© Bowe B et al. *JAMA Network Open*

eTables

eTable 1: Terms and definitions
**Age standardized rate**: Rate per 100,000 population following standardization to the global age structure. The difference between age-standardized rates across US geographies and over time is independent of population size and age structure.<br><br>**Decomposition:** The analytic approach to identify the additive contribution of the effect of the differences in factors in two populations to the difference in their overall value. For example, decomposition of CKD DALYs by age structure, population growth, and risk exposure changes allows the quantification of contribution of each of these factors to the overall effect. Similarly, decomposition of CKD DALYs by the four causes of CKD allows the quantification of the contribution of each cause to the overall CKD DALYs.<br><br>**Disability-adjusted life years (DALYs):** A measure which quantitates the overall burden of disease in terms of years of healthy life lost due to the disease. It represents the sum of years lost due to premature death (YLL) and years living with disability due to the disease (YLD). The years of living with disability are weighted in proportion to the duration and severity of the underlying disease.<br><br>**Frontier analysis:** The analytic approach used to identify the lowest potentially achievable burden of CKD on the basis of development status, as measured by the Socio-demographic index (SDI). The frontier delineates the countries or territories with leading performance (at the frontier pushing the envelope) which have the lowest CKD burden for their SDI. Distance from the frontier is termed effective difference and represents the gap between the observed burden and the potentially achievable burden of disease in a country or territory given their SDI; this gap could be potentially reduced or eliminated based on the country or territory's socio-demographic resources. For example, if a country or territory falls well below the frontier value given its SDI, this observation suggests unrealized opportunity for reduction (or improvement) in CKD DALYs that should be possible based on the country or territory's place on the development spectrum.<br><br>**Garbage codes**: refer to causes of death recorded on death certificates which cannot or should not be considered the actual underlying causes of death. For example, risk factors such as hypertension, or obesity, non-fatal health conditions such as osteoporosis, or limb fractures, and causes that represent the final stage of a disease such as cardiac arrest when ischemic heart disease is the true underlying health condition for cause of death.<br>**Global Burden of Diseases, Injuries, and Risk Factors Study (GBD):** A global research study group headquartered at the Institute of Health Metrics and Evaluation at the University of Washington in Seattle; it is comprised of more than 3,000 international collaborators from more than 130 countries and aims to quantitate the burden of disease, disability, and death from 333 diseases and injuries, and 88 risk factors in 195 countries and territories, by age and sex, from 1990 to the present, allowing comparisons over time, across age groups, and among populations. The GBD study is funded by the Bill and Melinda Gates Foundation.<br><br>**Level of Cause:** The GBD provides data for causes and disability aggregated to different levels, with the lowest level being the most aggregate, and higher levels being more specific. Level 1 consists of 3 broad categories: a) communicable, maternal, neonatal, and nutritional diseases, b) non-communicable diseases, and c) injuries. Level 2 causes are subdivided from Level 1 causes into 21 categories.<br><br>**Socio-demographic Index (SDI):** A summary measure which quantitates a state, country, or territory's level of a socio-demographic development; it interpretable across geographies and over time. SDI is

expressed on a scale of 0 to 1 and is the composite average of the rankings of the incomes per capita, average educational attainment, and total fertility rates of all areas in the GBD study. Zero represents the lowest income per capita, lowest educational attainment, and highest total fertility rate observed across all GBD geographies from 1970 to 2016, and one represents the highest income per capita, highest educational attainment, and lowest total fertility rate.

eTable 2: Number and age-standardized rate for overall DALY due to CKD and by cause, with percentage change from 2002 to 2016 for the United States and by state and district.

Location	DALY number		DALY per 100,000 persons		Age-standardized DALY per 100,000 persons									
					All causes		Due to diabetes		Due to hypertension		Due to glomerulonephritis		Due to other causes	
	2016 (UI)	Percent change from 2002	2016 (UI)	Percent change from 2002	2016 (UI)	Percent change from 2002	2016 (UI)	Percent change from 2002 (PA*)	2016 (UI)	Percent change from 2002 (PA*)	2016 (UI)	Percent change from 2002 (PA*)	2016 (UI)	Percent change from 2002 (PA*)
United States	1935954 (1747356, 2124795)	52.6	600 (541, 658)	35.9	440 (395, 485)	18.6	245 (216, 274)	21.8 (63.1)	83 (69, 97)	22 (21.6)	45 (36, 54)	10.4 (6.1)	68 (56, 81)	10.3 (9.2)
Alabama	40483 (35921, 45183)	46.2	833 (739, 929)	34.8	604 (534, 673)	19.4	337 (295, 381)	23.5 (65.3)	109 (90, 129)	18.7 (17.4)	64 (51, 77)	12.7 (7.3)	95 (78, 114)	11.6 (10.1)
Alaska	2790 (2404, 3158)	75.3	380 (327, 430)	54.1	359 (311, 407)	22.7	196 (168, 224)	26 (61)	68 (56, 82)	27.6 (22.1)	38 (30, 46)	13.4 (6.8)	57 (46, 69)	13.4 (10.1)
Arizona	37342 (32595, 41893)	77.2	540 (472, 606)	38.4	389 (338, 437)	17.6	216 (187, 248)	21.4 (65.5)	71 (58, 84)	18.6 (19.1)	41 (32, 49)	9.6 (6.1)	61 (49, 73)	9.8 (9.2)
Arkansas	22726 (20552, 25070)	54.3	764 (691, 843)	40.3	553 (498, 611)	27.8	308 (273, 346)	32.4 (62.7)	101 (84, 119)	30.9 (19.7)	58 (47, 69)	17.1 (7)	86 (71, 103)	17.4 (10.6)
California	189027 (165952, 212569)	62.6	479 (421, 539)	44	376 (329, 424)	21.1	210 (182, 241)	24.3 (62.4)	71 (58, 84)	28.4 (23.9)	38 (31, 47)	9.7 (5.2)	58 (47, 70)	10.8 (8.6)

© Bowe B et al. *JAMA Network Open*

Location	DALY number		DALY per 100,000 persons		Age-standardized DALY per 100,000 persons									
					All causes		Due to diabetes		Due to hypertension		Due to glomerulonephritis		Due to other causes	
	2016 (UI)	Percent change from 2002	2016 (UI)	Percent change from 2002	2016 (UI)	Percent change from 2002	2016 (UI)	Percent change from 2002 (PA*)	2016 (UI)	Percent change from 2002 (PA*)	2016 (UI)	Percent change from 2002 (PA*)	2016 (UI)	Percent change from 2002 (PA*)
Colorado	22956 (20031, 25747)	68.4	417 (364, 468)	37.2	330 (287, 373)	13.6	181 (154, 208)	16.5 (64.8)	64 (53, 76)	15.1 (21.1)	34 (27, 42)	7.5 (6)	51 (42, 62)	6.6 (8)
Connecticut	18643 (16407, 21018)	29.4	521 (459, 588)	24.6	356 (312, 404)	11.3	196 (168, 226)	13.2 (63)	68 (56, 81)	15 (24.6)	37 (29, 45)	5.2 (5)	55 (45, 67)	5.1 (7.4)
Delaware	6291 (5599, 6998)	65.9	660 (588, 734)	40.3	454 (403, 508)	17.8	252 (218, 285)	21.1 (63.9)	83 (68, 99)	19.7 (20)	48 (39, 58)	11.5 (7.2)	71 (59, 86)	9.4 (8.9)
District of Columbia	3911 (3436, 4348)	6	571 (502, 635)	-10.2	510 (449, 568)	-8	291 (254, 329)	-4.8 (33.1)	90 (74, 108)	-0.5 (1)	51 (41, 61)	-19.9 (28.7)	78 (63, 94)	-17.5 (37.2)
Florida	130246 (115380, 146384)	66.9	637 (564, 716)	36.2	402 (354, 455)	22.9	225 (196, 256)	27.2 (64.1)	73 (60, 85)	25.4 (19.6)	42 (33, 51)	13.1 (6.5)	63 (51, 76)	13.3 (9.8)
Georgia	71082 (63183, 79124)	64.3	691 (615, 770)	36.1	560 (497, 625)	12.7	312 (273, 354)	15.2 (65.4)	105 (87, 127)	16.2 (23.3)	57 (46, 68)	5.5 (4.7)	86 (71, 104)	5.1 (6.6)
Hawaii	7951 (6985, 8943)	64	552 (485, 621)	41.9	384 (335, 433)	22.7	215 (185, 246)	26.3 (62.9)	70 (58, 83)	25.2 (20)	39 (31, 48)	13.9 (6.8)	60 (48, 72)	14 (10.3)

© Bowe B et al. *JAMA Network Open*

Location	DALY number		DALY per 100,000 persons		Age-standardized DALY per 100,000 persons									
					All causes		Due to diabetes		Due to hypertension		Due to glomerulonephritis		Due to other causes	
	2016 (UI)	Percent change from 2002	2016 (UI)	Percent change from 2002	2016 (UI)	Percent change from 2002	2016 (UI)	Percent change from 2002 (PA*)	2016 (UI)	Percent change from 2002 (PA*)	2016 (UI)	Percent change from 2002 (PA*)	2016 (UI)	Percent change from 2002 (PA*)
Idaho	8085 (7076, 9126)	87.4	482 (422, 544)	49.6	367 (320, 415)	28.2	201 (172, 232)	31.4 (59.4)	71 (58, 84)	32.5 (21.6)	38 (31, 47)	19.5 (7.7)	57 (46, 70)	18.9 (11.3)
Illinois	79218 (71390, 87002)	31.3	617 (556, 677)	28.2	456 (411, 504)	10.4	253 (223, 285)	12.8 (66.3)	88 (73, 103)	16 (28.1)	46 (37, 55)	2 (2)	70 (57, 84)	2.3 (3.6)
Indiana	46127 (41109, 51279)	50.9	696 (620, 773)	40.1	515 (457, 574)	23.7	283 (245, 323)	26.8 (60.7)	99 (82, 118)	26 (20.7)	53 (43, 63)	16.8 (7.7)	80 (66, 96)	15.6 (10.9)
Iowa	15897 (13923, 17920)	49.4	507 (444, 572)	39.7	349 (304, 396)	30.1	192 (165, 220)	33.3 (59.3)	68 (56, 81)	35.6 (22)	36 (29, 44)	20.3 (7.5)	54 (43, 66)	20.2 (11.2)
Kansas	17463 (15499, 19555)	42.5	601 (533, 672)	32.9	443 (391, 498)	22.2	245 (211, 279)	26.1 (62.8)	85 (70, 100)	23.3 (19.9)	45 (37, 55)	14.3 (7)	69 (56, 83)	13.9 (10.4)
Kentucky	33485 (29993, 36835)	56.5	757 (678, 832)	44.6	550 (491, 605)	26.3	305 (267, 343)	30.4 (62)	104 (87, 122)	27.2 (19.4)	56 (45, 67)	18 (7.5)	85 (70, 101)	17.7 (11.2)
Louisiana	41215 (37500, 45293)	39.7	881 (801, 968)	34.3	681 (619, 749)	16.9	381 (339, 424)	20 (64.4)	124 (105, 145)	20.6 (21.6)	70 (57, 84)	8.7 (5.7)	106 (88, 126)	8.4 (8.4)

© Bowe B et al. *JAMA Network Open*

Location	DALY number		DALY per 100,000 persons		Age-standardized DALY per 100,000 persons									
					All causes		Due to diabetes		Due to hypertension		Due to glomerulonephritis		Due to other causes	
	2016 (UI)	Percent change from 2002	2016 (UI)	Percent change from 2002	2016 (UI)	Percent change from 2002	2016 (UI)	Percent change from 2002 (PA*)	2016 (UI)	Percent change from 2002 (PA*)	2016 (UI)	Percent change from 2002 (PA*)	2016 (UI)	Percent change from 2002 (PA*)
Maine	8550 (7594, 9506)	44.9	644 (572, 716)	41	394 (349, 441)	17.3	215 (186, 244)	19.6 (60.5)	77 (63, 91)	18.8 (20.9)	41 (34, 50)	11.8 (7.5)	62 (50, 74)	11.8 (11.2)
Maryland	34418 (30696, 38351)	40.9	571 (509, 636)	27	427 (380, 477)	9.3	237 (207, 269)	11.6 (67.4)	79 (66, 94)	14 (26.7)	44 (36, 54)	1.7 (2)	66 (55, 80)	2.2 (3.9)
Massachusetts	37343 (33097, 41760)	29	547 (485, 612)	21	381 (335, 429)	8.8	210 (181, 239)	10.6 (65.1)	75 (62, 89)	11.9 (25.9)	38 (31, 46)	3.7 (4.4)	58 (47, 71)	2.5 (4.5)
Michigan	63694 (56882, 70592)	39.4	643 (574, 712)	40.6	448 (399, 499)	17.4	247 (217, 278)	20.3 (62.6)	85 (71, 101)	19.8 (21.1)	46 (37, 55)	10.4 (6.5)	70 (57, 83)	10.3 (9.8)
Minnesota	27902 (24496, 31136)	51.3	507 (445, 565)	37.6	363 (316, 410)	19.4	197 (170, 226)	22.6 (61.3)	73 (60, 87)	22.3 (22.5)	37 (29, 45)	11.4 (6.4)	56 (45, 68)	11.5 (9.8)
Mississippi	27469 (24432, 30656)	48.4	919 (817, 1025)	41.9	697 (620, 779)	23.4	392 (343, 442)	27.5 (63.9)	125 (104, 148)	25.9 (19.4)	73 (58, 87)	13.8 (6.7)	109 (90, 130)	13.9 (10)
Missouri	41045 (36920, 45196)	49.2	675 (607, 743)	39.1	477 (427, 527)	23.5	265 (232, 298)	27.7 (63)	90 (76, 106)	25.2 (19.9)	49 (39, 59)	14.5 (6.8)	74 (61, 89)	14.5 (10.3)

© Bowe B et al. *JAMA Network Open*

Location	DALY number		DALY per 100,000 persons		Age-standardized DALY per 100,000 persons									
					All causes		Due to diabetes		Due to hypertension		Due to glomerulonephritis		Due to other causes	
	2016 (UI)	Percent change from 2002	2016 (UI)	Percent change from 2002	2016 (UI)	Percent change from 2002	2016 (UI)	Percent change from 2002 (PA*)	2016 (UI)	Percent change from 2002 (PA*)	2016 (UI)	Percent change from 2002 (PA*)	2016 (UI)	Percent change from 2002 (PA*)
Montana	5085 (4415, 5760)	52.3	490 (426, 556)	33.7	333 (288, 377)	17	182 (154, 210)	20.7 (64.4)	63 (51, 75)	17.8 (19.5)	36 (28, 44)	9.9 (6.6)	53 (43, 64)	9.5 (9.5)
Nebraska	10479 (9331, 11643)	44	550 (490, 611)	30.7	405 (360, 452)	21.3	222 (192, 255)	24.5 (61.3)	78 (65, 93)	24.9 (22)	42 (33, 50)	13.3 (6.9)	63 (51, 75)	12.6 (9.9)
Nevada	17919 (15875, 19888)	70.7	611 (542, 678)	26.2	463 (410, 514)	6.3	258 (225, 292)	9.1 (77.7)	86 (72, 101)	6.6 (19.1)	47 (38, 57)	1.9 (3.1)	72 (59, 85)	0 (0.1)
New Hampshire	6884 (6030, 7756)	56.7	516 (452, 582)	48.4	343 (299, 389)	19.1	187 (160, 215)	20.9 (58.8)	67 (54, 79)	21 (21)	36 (29, 45)	15.9 (9.1)	54 (44, 66)	12.8 (11.1)
New Jersey	51137 (45268, 57293)	26	571 (505, 639)	20	405 (357, 454)	6.8	224 (196, 257)	8.7 (68.8)	78 (64, 93)	10.4 (28.2)	41 (33, 50)	1.6 (2.5)	62 (51, 75)	0.2 (0.6)
New Mexico	12684 (11097, 14230)	75.2	612 (535, 687)	57	445 (389, 500)	30.7	247 (213, 283)	34.9 (61)	81 (66, 96)	32.4 (18.9)	47 (38, 57)	22.2 (8.2)	70 (57, 84)	21.7 (11.9)
New York	100344 (88124, 113215)	29.3	506 (445, 571)	25.1	366 (321, 416)	10.8	203 (174, 234)	12.5 (63.3)	69 (56, 83)	15.3 (25.6)	38 (30, 46)	4.6 (4.6)	57 (46, 69)	4.2 (6.5)

© Bowe B et al. *JAMA Network Open*

Location	DALY number		DALY per 100,000 persons		Age-standardized DALY per 100,000 persons									
					All causes		Due to diabetes		Due to hypertension		Due to glomerulonephritis		Due to other causes	
	2016 (UI)	Percent change from 2002	2016 (UI)	Percent change from 2002	2016 (UI)	Percent change from 2002	2016 (UI)	Percent change from 2002 (PA*)	2016 (UI)	Percent change from 2002 (PA*)	2016 (UI)	Percent change from 2002 (PA*)	2016 (UI)	Percent change from 2002 (PA*)
North Carolina	71062 (64146, 78431)	67.2	703 (635, 776)	37.9	515 (463, 570)	18	288 (253, 323)	21 (63.6)	96 (80, 113)	20.1 (20.4)	52 (42, 63)	11.5 (6.9)	79 (65, 95)	9.9 (9.1)
North Dakota	3781 (3326, 4230)	45.9	491 (432, 549)	20.9	376 (328, 422)	23.9	206 (177, 237)	27.1 (60.5)	71 (58, 84)	25.9 (20)	40 (32, 49)	17.2 (8)	59 (48, 72)	16.4 (11.5)
Ohio	79979 (71828, 88129)	40.6	689 (619, 759)	38.2	482 (432, 532)	20.7	267 (236, 300)	24 (62.5)	91 (76, 107)	22.3 (20)	49 (40, 59)	13.8 (7.2)	74 (61, 90)	13 (10.3)
Oklahoma	25736 (23271, 28444)	60.7	658 (595, 727)	43.3	500 (451, 553)	32.9	278 (246, 313)	37.8 (61.7)	92 (77, 109)	34.1 (18.9)	52 (42, 62)	22.7 (7.8)	78 (65, 93)	22.7 (11.7)
Oregon	20206 (17617, 22570)	63	500 (436, 558)	41.5	342 (296, 384)	21.3	188 (161, 215)	24.1 (60.8)	67 (55, 78)	24.9 (22.2)	35 (28, 42)	14.1 (7.2)	53 (43, 64)	12.6 (9.8)
Pennsylvania	86406 (77442, 95932)	27.1	677 (607, 751)	22.5	445 (396, 496)	12	246 (215, 279)	14.4 (64.9)	85 (71, 100)	15 (23.4)	45 (37, 54)	5.5 (4.9)	69 (55, 83)	5 (6.8)
Rhode Island	5502 (4752, 6195)	28.9	521 (450, 587)	29.8	355 (307, 403)	16.8	196 (167, 225)	19.2 (61.4)	68 (55, 81)	20.9 (22.9)	37 (29, 45)	9.7 (6.4)	55 (45, 67)	9.6 (9.4)

© Bowe B et al. *JAMA Network Open*

Location	DALY number		DALY per 100,000 persons		Age-standardized DALY per 100,000 persons									
					All causes		Due to diabetes		Due to hypertension		Due to glomerulonephritis		Due to other causes	
	2016 (UI)	Percent change from 2002	2016 (UI)	Percent change from 2002	2016 (UI)	Percent change from 2002	2016 (UI)	Percent change from 2002 (PA*)	2016 (UI)	Percent change from 2002 (PA*)	2016 (UI)	Percent change from 2002 (PA*)	2016 (UI)	Percent change from 2002 (PA*)
South Carolina	38273 (34135, 42441)	65.7	776 (692, 860)	37.8	550 (488, 611)	15.1	307 (269, 349)	17.7 (63.9)	102 (85, 121)	19.2 (22.8)	56 (46, 67)	7.9 (5.7)	85 (70, 102)	7 (7.6)
South Dakota	4397 (3862, 4932)	50.2	508 (446, 570)	32	365 (319, 411)	22.7	200 (171, 229)	26.4 (61.9)	68 (56, 80)	24.4 (19.7)	39 (31, 48)	14.5 (7.4)	58 (48, 71)	14.6 (11)
Tennessee	45521 (40988, 50183)	67.8	687 (618, 757)	46.8	501 (449, 553)	27.9	279 (245, 313)	32.3 (62.4)	93 (77, 110)	31.8 (20.5)	52 (42, 62)	16.4 (6.7)	77 (64, 92)	17.4 (10.5)
Texas	162865 (145554, 180379)	92	585 (523, 648)	49.9	503 (448, 556)	30.9	282 (246, 316)	35 (61.5)	94 (79, 113)	34.9 (20.5)	50 (41, 61)	19.7 (7)	77 (63, 92)	20.5 (11)
Utah	12062 (10675, 13524)	83.4	398 (353, 447)	41.7	388 (342, 435)	23.4	212 (183, 240)	26.2 (60)	76 (63, 90)	27.3 (22.3)	39 (32, 48)	15.2 (7.1)	60 (49, 73)	15 (10.6)
Vermont	3082 (2704, 3476)	43	494 (433, 557)	40.6	321 (281, 363)	15.8	175 (151, 203)	18 (60.8)	62 (51, 74)	18.2 (21.6)	34 (28, 41)	11 (7.7)	50 (41, 61)	9.5 (9.9)
Virginia	52548 (47199, 58081)	53.9	624 (560, 690)	33.1	468 (419, 518)	13.9	260 (227, 294)	16.5 (64.3)	90 (75, 106)	17.3 (23.1)	47 (38, 56)	6.6 (5.1)	72 (59, 86)	6.3 (7.5)

© Bowe B et al. *JAMA Network Open*

Location	DALY number		DALY per 100,000 persons		Age-standardized DALY per 100,000 persons									
					All causes		Due to diabetes		Due to hypertension		Due to glomerulonephritis		Due to other causes	
	2016 (UI)	Percent change from 2002	2016 (UI)	Percent change from 2002	2016 (UI)	Percent change from 2002	2016 (UI)	Percent change from 2002 (PA*)	2016 (UI)	Percent change from 2002 (PA*)	2016 (UI)	Percent change from 2002 (PA*)	2016 (UI)	Percent change from 2002 (PA*)
Washington	31904 (27876, 35781)	79.4	442 (386, 496)	50.3	328 (285, 369)	28.5	180 (154, 207)	31.8 (59.7)	64 (52, 76)	34.1 (22.3)	34 (27, 41)	17.7 (6.9)	51 (41, 62)	19 (11.1)
West Virginia	16506 (14969, 18203)	48.3	901 (817, 994)	46.1	587 (529, 647)	31.3	326 (289, 366)	35.6 (61.2)	109 (91, 128)	32.3 (19)	61 (49, 72)	22.4 (8)	91 (76, 109)	22.1 (11.8)
Wisconsin	33598 (29912, 37307)	49.6	582 (518, 646)	40.8	401 (355, 447)	21.8	219 (191, 249)	24.8 (60.4)	79 (66, 93)	26 (22.8)	41 (33, 50)	13.2 (6.6)	62 (51, 75)	13.5 (10.2)
Wyoming	2634 (2282, 2982)	54.1	451 (391, 510)	31.7	343 (297, 389)	17.8	189 (161, 218)	21.3 (64.1)	64 (52, 76)	19.9 (20.5)	36 (29, 44)	9.8 (6.3)	54 (44, 66)	9.7 (9.2)

DALY: Disability Adjusted Life Year
UI is the uncertainty interval, it reflects the certainty of an estimate based on data availability, studies size and consistency across data sources
*PA: Percentage attributed to overall rate is the proportion of overall age-standardized rate change attributed by each of the following four causes: diabetes, hypertension, glomerulonephritis and other causes

© Bowe B et al. *JAMA Network Open*

eTable 3: Number and age-standardized rate for overall Deaths due to CKD and by cause, with percentage change from 2002 to 2016 for the United States and by state and district.

Location	Death number		Death per 100,000 persons		Age-standardized Death per 100,000 persons									
					All causes		Due to diabetes		Due to hypertension		Due to glomerulonephritis		Due to other causes	
	2016 (UI)	Percent change from 2002	2016 (UI)	Percent change from 2002	2016 (UI)	Percent change from 2002	2016 (UI)	Percent change from 2002 (PA*)	2016 (UI)	Percent change from 2002 (PA*)	2016 (UI)	Percent change from 2002 (PA*)	2016 (UI)	Percent change from 2002 (PA*)
United States	82539 (80298, 84652)	58.3	26 (25, 26)	41.1	16 (16, 17)	17.9	8 (8, 9)	20 (55.6)	5 (4, 5)	19.8 (30.6)	1 (1, 2)	11.1 (4.9)	2 (2, 3)	11 (8.9)
Alabama	1703 (1518, 1890)	40.1	35 (31, 39)	29.2	23 (20, 25)	11.2	12 (10, 14)	15.3 (68)	6 (5, 7)	8.1 (19.9)	2 (1, 2)	7.3 (5.2)	3 (3, 4)	5.3 (7)
Alaska	88 (78, 98)	117	12 (11, 13)	90.8	13 (11, 14)	30.1	6 (5, 7)	30.1 (49.3)	4 (3, 4)	37.2 (34.2)	1 (1, 1)	20.7 (5.5)	2 (1, 2)	22.3 (10.9)
Arizona	1447 (1320, 1577)	81.1	21 (19, 23)	41.5	13 (12, 14)	11.7	7 (6, 8)	15.2 (64.3)	4 (3, 4)	10.5 (24.9)	1 (1, 1)	6.3 (4.2)	2 (1, 2)	5.3 (6.5)
Arkansas	1006 (925, 1090)	59	34 (31, 37)	44.5	21 (20, 23)	29.2	11 (10, 12)	32.6 (55.7)	6 (5, 7)	29.4 (27.4)	2 (1, 2)	21.8 (6.1)	3 (2, 4)	21.4 (10.8)
California	7415 (6665, 8159)	89.2	19 (17, 21)	67.6	13 (12, 15)	31.5	7 (6, 8)	32.7 (52.3)	4 (3, 4)	36.9 (31.9)	1 (1, 1)	21.3 (5.3)	2 (1, 2)	22.7 (10.5)

© Bowe B et al. *JAMA Network Open*

Location	Death number		Death per 100,000 persons		Age-standardized Death per 100,000 persons									
					All causes		Due to diabetes		Due to hypertension		Due to glomerulonephritis		Due to other causes	
	2016 (UI)	Percent change from 2002	2016 (UI)	Percent change from 2002	2016 (UI)	Percent change from 2002	2016 (UI)	Percent change from 2002 (PA*)	2016 (UI)	Percent change from 2002 (PA*)	2016 (UI)	Percent change from 2002 (PA*)	2016 (UI)	Percent change from 2002 (PA*)
Colorado	891 (817, 974)	74.8	16 (15, 18)	42.5	12 (11, 13)	9.3	6 (5, 7)	11.2 (58.2)	4 (3, 4)	10.8 (34.1)	1 (1, 1)	3.2 (2.7)	2 (1, 2)	3.3 (5.1)
Connecticut	834 (755, 925)	34.7	23 (21, 26)	29.7	13 (12, 14)	9.3	6 (5, 7)	9 (48.1)	4 (3, 5)	14.6 (44.3)	1 (1, 1)	2.8 (2.4)	2 (1, 2)	3.4 (5.3)
Delaware	261 (240, 281)	74.1	27 (25, 30)	47.3	16 (15, 18)	15.4	8 (7, 9)	16.9 (55)	5 (4, 5)	17.8 (31.7)	1 (1, 2)	9.6 (5)	2 (2, 3)	8.7 (8.3)
District of Columbia	154 (137, 170)	5.1	22 (20, 25)	-11	19 (17, 21)	-8.5	10 (9, 11)	-6.6 (41)	5 (4, 6)	-4.6 (13)	1 (1, 2)	-18.2 (18.2)	3 (2, 3)	-15.9 (27.9)
Florida	5401 (4948, 5917)	70.9	26 (24, 29)	39.4	13 (12, 15)	20.5	7 (6, 8)	24.7 (60.1)	4 (3, 4)	19.1 (25.4)	1 (1, 1)	13.4 (5.2)	2 (2, 2)	13 (9.3)
Georgia	2926 (2644, 3253)	69.6	28 (26, 32)	40.5	22 (20, 25)	10.6	11 (10, 13)	12 (57.2)	6 (5, 7)	13.3 (34.1)	2 (1, 2)	4 (3)	3 (2, 4)	4.1 (5.7)
Hawaii	333 (307, 359)	85.2	23 (21, 25)	60.3	13 (12, 14)	21.9	7 (6, 7)	25.3 (57.6)	4 (3, 4)	21.5 (27.1)	1 (1, 1)	15.8 (5.6)	2 (1, 2)	14.7 (9.7)

© Bowe B et al. *JAMA Network Open*

Location	Death number		Death per 100,000 persons		Age-standardized Death per 100,000 persons									
					All causes		Due to diabetes		Due to hypertension		Due to glomerulonephritis		Due to other causes	
	2016 (UI)	Percent change from 2002	2016 (UI)	Percent change from 2002	2016 (UI)	Percent change from 2002	2016 (UI)	Percent change from 2002 (PA*)	2016 (UI)	Percent change from 2002 (PA*)	2016 (UI)	Percent change from 2002 (PA*)	2016 (UI)	Percent change from 2002 (PA*)
Idaho	340 (305, 377)	105.4	20 (18, 22)	64	14 (12, 15)	34.6	7 (6, 8)	36.8 (51.2)	4 (3, 5)	36.7 (31.3)	1 (1, 1)	27.2 (6)	2 (1, 2)	27.3 (11.4)
Illinois	3610 (3335, 3905)	37.2	28 (26, 30)	34	18 (17, 20)	11.1	9 (8, 10)	12.1 (54.5)	5 (4, 6)	15.1 (37.6)	1 (1, 2)	3.4 (2.4)	2 (2, 3)	4.2 (5.6)
Indiana	2150 (1938, 2386)	56.4	32 (29, 36)	45.1	21 (19, 23)	23.6	11 (9, 12)	25.5 (53.2)	6 (5, 7)	25.3 (30.6)	2 (1, 2)	17.7 (5.8)	3 (2, 4)	16.8 (10.3)
Iowa	724 (664, 794)	63.1	23 (21, 25)	52.5	13 (11, 14)	41	6 (5, 7)	43.2 (51.1)	4 (3, 4)	43.8 (31.2)	1 (1, 1)	32.1 (6.1)	2 (1, 2)	32.8 (11.6)
Kansas	812 (727, 903)	40.4	28 (25, 31)	30.9	17 (15, 19)	19.1	9 (7, 10)	22.7 (57.9)	5 (4, 6)	18 (27.2)	1 (1, 2)	13.4 (5.4)	2 (2, 3)	12.4 (9.4)
Kentucky	1482 (1370, 1609)	58.8	33 (31, 36)	46.8	22 (20, 24)	24.1	11 (10, 13)	27.4 (56.1)	6 (5, 7)	23.4 (27.5)	2 (1, 2)	18.4 (6)	3 (2, 4)	17.3 (10.4)
Louisiana	1798 (1669, 1940)	41.7	38 (36, 41)	36.3	28 (26, 30)	15.2	14 (13, 16)	17 (56.8)	7 (6, 9)	17.6 (30.6)	2 (2, 3)	8.4 (4.5)	4 (3, 5)	8.3 (8.1)

© Bowe B et al. *JAMA Network Open*

Location	Death number		Death per 100,000 persons		Age-standardized Death per 100,000 persons									
					All causes		Due to diabetes		Due to hypertension		Due to glomerulonephritis		Due to other causes	
	2016 (UI)	Percent change from 2002	2016 (UI)	Percent change from 2002	2016 (UI)	Percent change from 2002	2016 (UI)	Percent change from 2002 (PA*)	2016 (UI)	Percent change from 2002 (PA*)	2016 (UI)	Percent change from 2002 (PA*)	2016 (UI)	Percent change from 2002 (PA*)
Maine	400 (369, 432)	50.1	30 (28, 33)	46.1	15 (14, 17)	15.5	7 (7, 8)	16.5 (51.6)	5 (4, 5)	18.3 (34.6)	1 (1, 1)	9.6 (4.8)	2 (2, 3)	9.6 (9)
Maryland	1384 (1281, 1497)	48.9	23 (21, 25)	34.2	15 (14, 17)	7.4	8 (7, 9)	8.4 (56.9)	4 (4, 5)	11.3 (40.4)	1 (1, 1)	0.6 (0.7)	2 (2, 3)	1 (2)
Massachusetts	1768 (1615, 1916)	29.8	26 (24, 28)	21.8	15 (14, 16)	5.4	7 (6, 8)	5.7 (50.9)	4 (4, 5)	9.2 (47.9)	1 (1, 1)	-0.4 (0.6)	2 (2, 3)	-0.2 (0.6)
Michigan	2828 (2623, 3032)	46.6	29 (26, 31)	47.9	17 (16, 18)	15.8	9 (8, 10)	17.7 (55.4)	5 (4, 6)	17.3 (30.9)	1 (1, 2)	9.9 (4.9)	2 (2, 3)	9.5 (8.7)
Minnesota	1352 (1236, 1476)	58.2	25 (22, 27)	43.8	15 (13, 16)	20.5	7 (6, 8)	22.3 (51.8)	5 (4, 5)	22.4 (33.3)	1 (1, 1)	13.7 (5.1)	2 (2, 2)	13.9 (9.8)
Mississippi	1179 (1059, 1310)	48.3	39 (35, 44)	41.8	27 (24, 30)	20.7	14 (12, 16)	24.1 (59)	7 (6, 9)	20.3 (25.7)	2 (2, 3)	14.3 (5.7)	4 (3, 5)	13.5 (9.6)
Missouri	1851 (1711, 2001)	50.2	30 (28, 33)	40	18 (17, 20)	21.5	9 (8, 10)	24.7 (56.7)	5 (4, 6)	21.3 (28)	1 (1, 2)	14.9 (5.5)	3 (2, 3)	14.4 (9.8)

© Bowe B et al. *JAMA Network Open*

Location	Death number		Death per 100,000 persons		Age-standardized Death per 100,000 persons									
					All causes		Due to diabetes		Due to hypertension		Due to glomerulonephritis		Due to other causes	
	2016 (UI)	Percent change from 2002	2016 (UI)	Percent change from 2002	2016 (UI)	Percent change from 2002	2016 (UI)	Percent change from 2002 (PA*)	2016 (UI)	Percent change from 2002 (PA*)	2016 (UI)	Percent change from 2002 (PA*)	2016 (UI)	Percent change from 2002 (PA*)
Montana	209 (187, 233)	53	20 (18, 23)	34.4	11 (10, 13)	12.3	6 (5, 7)	15.3 (59.8)	3 (3, 4)	11.4 (27.3)	1 (1, 1)	7.9 (5)	2 (1, 2)	6.6 (7.9)
Nebraska	491 (450, 531)	47.5	26 (24, 28)	33.8	16 (14, 17)	22.6	8 (7, 9)	24 (51.9)	5 (4, 5)	25.4 (32.6)	1 (1, 1)	15.9 (5.4)	2 (2, 3)	15.7 (10.1)
Nevada	701 (639, 763)	71.2	24 (22, 26)	26.6	17 (16, 19)	-2.8	9 (8, 10)	-0.7 (11.7)	5 (4, 6)	-2.7 (26.4)	1 (1, 2)	-7 (19.6)	2 (2, 3)	-8.1 (42.2)
New Hampshire	295 (271, 320)	70.8	22 (20, 24)	61.7	12 (11, 14)	18.8	6 (5, 7)	20.3 (52)	4 (3, 4)	20.4 (32)	1 (1, 1)	14.3 (5.8)	2 (1, 2)	13.3 (10.2)
New Jersey	2274 (2079, 2475)	28.3	25 (23, 28)	22.2	15 (14, 17)	2.9	8 (7, 9)	3.6 (42.6)	4 (4, 5)	6.2 (41.5)	1 (1, 1)	-3.2 (5.9)	2 (2, 3)	-2.9 (9.9)
New Mexico	510 (454, 569)	92.5	25 (22, 27)	72.5	16 (14, 18)	32.4	8 (7, 9)	35.8 (55)	4 (4, 5)	32.1 (27.1)	1 (1, 1)	26.4 (6.6)	2 (2, 3)	24.9 (11.3)
New York	4006 (3589, 4458)	33.2	20 (18, 23)	28.8	12 (11, 14)	7.9	6 (5, 7)	8.1 (51.8)	4 (3, 4)	12.9 (44.2)	1 (1, 1)	1 (1)	2 (1, 2)	1.6 (3)

© Bowe B et al. *JAMA Network Open*

Location	Death number		Death per 100,000 persons		Age-standardized Death per 100,000 persons									
					All causes		Due to diabetes		Due to hypertension		Due to glomerulonephritis		Due to other causes	
	2016 (UI)	Percent change from 2002	2016 (UI)	Percent change from 2002	2016 (UI)	Percent change from 2002	2016 (UI)	Percent change from 2002 (PA*)	2016 (UI)	Percent change from 2002 (PA*)	2016 (UI)	Percent change from 2002 (PA*)	2016 (UI)	Percent change from 2002 (PA*)
North Carolina	3009 (2785, 3235)	71.4	30 (28, 32)	41.3	20 (18, 21)	14.6	10 (9, 11)	17.1 (58.6)	5 (5, 6)	15.3 (28.6)	1 (1, 2)	8.7 (4.7)	3 (2, 3)	8.1 (8)
North Dakota	173 (156, 189)	46.8	22 (20, 25)	21.7	13 (12, 15)	23.7	7 (6, 8)	26.2 (53.4)	4 (3, 5)	24 (29.6)	1 (1, 1)	18.8 (6.1)	2 (1, 2)	17.8 (10.9)
Ohio	3566 (3319, 3824)	43.5	31 (29, 33)	41	18 (17, 20)	17.6	9 (8, 11)	20 (56.5)	5 (4, 6)	18.1 (28.8)	1 (1, 2)	12.2 (5.4)	3 (2, 3)	11.1 (9.2)
Oklahoma	1083 (1000, 1166)	61.4	28 (26, 30)	43.9	19 (17, 20)	32.3	10 (8, 11)	37 (56.5)	5 (4, 6)	30 (25.9)	1 (1, 2)	26 (6.4)	3 (2, 3)	24.6 (11.2)
Oregon	865 (798, 932)	72.6	21 (20, 23)	49.8	12 (12, 13)	24	6 (5, 7)	25.6 (52)	4 (3, 4)	26.8 (32.6)	1 (1, 1)	16.3 (5.2)	2 (1, 2)	16.8 (10.1)
Pennsylvania	4170 (3879, 4494)	30.6	33 (30, 35)	25.9	17 (16, 19)	10.1	9 (8, 10)	11.2 (54.9)	5 (4, 6)	13.1 (36.5)	1 (1, 2)	3.9 (3)	2 (2, 3)	3.9 (5.6)
Rhode Island	243 (218, 268)	37.1	23 (21, 25)	38	13 (11, 14)	19.4	6 (5, 7)	19.6 (50.4)	4 (3, 4)	24.4 (35.1)	1 (1, 1)	12.5 (5)	2 (1, 2)	12.7 (9.5)

© Bowe B et al. *JAMA Network Open*

Location	Death number		Death per 100,000 persons		Age-standardized Death per 100,000 persons									
					All causes		Due to diabetes		Due to hypertension		Due to glomerulonephritis		Due to other causes	
	2016 (UI)	Percent change from 2002	2016 (UI)	Percent change from 2002	2016 (UI)	Percent change from 2002	2016 (UI)	Percent change from 2002 (PA*)	2016 (UI)	Percent change from 2002 (PA*)	2016 (UI)	Percent change from 2002 (PA*)	2016 (UI)	Percent change from 2002 (PA*)
South Carolina	1630 (1477, 1806)	73.1	33 (30, 37)	43.9	21 (19, 24)	13.9	11 (10, 13)	14.7 (53.7)	6 (5, 7)	18.2 (34.8)	2 (1, 2)	7 (4.1)	3 (2, 4)	7.1 (7.4)
South Dakota	192 (175, 212)	51.4	22 (20, 24)	33.1	12 (11, 14)	21.1	6 (5, 7)	23.8 (54.8)	4 (3, 4)	21.1 (28.7)	1 (1, 1)	16.1 (6.1)	2 (1, 2)	14.9 (10.4)
Tennessee	1906 (1757, 2041)	79.3	29 (26, 31)	56.8	19 (18, 20)	31.6	10 (8, 11)	34.2 (54.1)	5 (4, 6)	33.8 (29.2)	1 (1, 2)	23 (5.9)	3 (2, 3)	23.2 (10.8)
Texas	6449 (5974, 6974)	101.9	23 (21, 25)	57.6	19 (18, 21)	32.9	10 (9, 11)	36.5 (55.5)	5 (4, 6)	33 (27.6)	1 (1, 2)	24.9 (6)	3 (2, 3)	24.7 (10.9)
Utah	479 (443, 518)	94.5	16 (15, 17)	50.4	15 (14, 16)	27.5	7 (6, 8)	29.5 (51.7)	4 (4, 5)	29.7 (31.8)	1 (1, 1)	20.4 (5.7)	2 (2, 3)	20.4 (10.8)
Vermont	131 (120, 143)	56.7	21 (19, 23)	54	11 (10, 12)	16.4	5 (5, 6)	16.4 (48.5)	3 (3, 4)	20.9 (37)	1 (1, 1)	10.6 (5)	2 (1, 2)	10.8 (9.5)
Virginia	2274 (2099, 2460)	62.1	27 (25, 29)	40.2	19 (17, 20)	12.8	9 (8, 11)	13.7 (53.2)	5 (5, 6)	16.8 (36.3)	1 (1, 2)	5.8 (3.5)	3 (2, 3)	6.2 (7)

© Bowe B et al. *JAMA Network Open*

Location	Death number		Death per 100,000 persons		Age-standardized Death per 100,000 persons									
					All causes		Due to diabetes		Due to hypertension		Due to glomerulonephritis		Due to other causes	
	2016 (UI)	Percent change from 2002	2016 (UI)	Percent change from 2002	2016 (UI)	Percent change from 2002	2016 (UI)	Percent change from 2002 (PA*)	2016 (UI)	Percent change from 2002 (PA*)	2016 (UI)	Percent change from 2002 (PA*)	2016 (UI)	Percent change from 2002 (PA*)
Washington	1281 (1186, 1382)	102.3	18 (16, 19)	69.4	12 (11, 13)	38.1	6 (5, 7)	40.1 (51)	3 (3, 4)	41.4 (31.8)	1 (1, 1)	29 (5.8)	2 (1, 2)	29.9 (11.3)
West Virginia	754 (698, 820)	51	41 (38, 45)	48.8	23 (22, 25)	30.6	12 (10, 13)	34 (55.1)	6 (5, 8)	30.5 (27.7)	2 (1, 2)	23.7 (6.2)	3 (3, 4)	23 (11)
Wisconsin	1613 (1486, 1728)	59.2	28 (26, 30)	49.9	16 (15, 17)	25.3	8 (7, 9)	26.4 (50.6)	5 (4, 6)	28.4 (33)	1 (1, 1)	18.7 (5.7)	2 (2, 3)	18.6 (10.7)
Wyoming	101 (91, 112)	58.5	17 (16, 19)	35.4	12 (11, 13)	15.4	6 (5, 7)	18.3 (58)	3 (3, 4)	15.6 (29)	1 (1, 1)	9.2 (4.7)	2 (1, 2)	8.8 (8.3)

UI is the uncertainty interval, it reflects the certainty of an estimate based on data availability, studies size and consistency across data sources
*PA: Percentage attributed to overall rate is the proportion of overall age-standardized rate change attributed by each of the following four causes: diabetes, hypertension, glomerulonephritis and other causes

© Bowe B et al. *JAMA Network Open*

eTable4: Years living with disability (YLD) due to CKD in the United States and by state and district in the overall population, and in females and males.

eTable 4a: Number and age-standardized rate for overall YLD due to CKD and by cause, with percentage change from 2002 to 2016 for the Unites States and by state and district.

location	YLD number		YLD per 100,000 persons		Age-standardized YLD per 100,000 persons									
					All causes		Due to diabetes		Due to hypertension		Due to glomerulonephritis		Due to other causes	
	2016 (UI)	Percent change from 2002	2016 (UI)	Percent change from 2002	2016 (UI)	Percent change from 2002	2016 (UI)	Percent change from 2002 (PA*)	2016 (UI)	Percent change from 2002 (PA*)	2016 (UI)	Percent change from 2002 (PA*)	2016 (UI)	Percent change from 2002 (PA*)
United States	723448 (539476, 901307)	47.8	224 (167, 279)	31.7	170 (127, 212)	17.7	93 (69, 118)	20.4 (61.8)	30 (21, 38)	21 (20.1)	20 (14, 26)	10.6 (7.4)	28 (20, 37)	11 (10.7)
Alabama	12405 (9175, 15605)	42.5	255 (189, 321)	31.4	190 (141, 239)	18	104 (76, 132)	21.1 (62.5)	33 (23, 43)	21 (19.7)	23 (16, 30)	10.6 (7.4)	31 (22, 41)	10.7 (10.4)
Alaska	1245 (932, 1553)	63.7	169 (127, 211)	43.9	159 (119, 197)	18.8	87 (64, 110)	22.4 (63.2)	28 (20, 36)	20.8 (18.9)	18 (13, 24)	11.6 (7.6)	26 (18, 34)	11.2 (10.3)
Arizona	15414 (11426, 19179)	76.2	223 (165, 278)	37.7	165 (123, 205)	18.8	90 (67, 114)	21.6 (61.4)	29 (20, 37)	21.5 (19.4)	19 (14, 25)	11 (7.3)	27 (19, 35)	13 (11.8)
Arkansas	7161 (5280, 9030)	42	241 (178, 304)	29.2	179 (133, 225)	19.4	98 (71, 125)	22.8 (62.7)	31 (22, 41)	22.1 (19.3)	21 (15, 28)	11.5 (7.5)	29 (20, 39)	11.8 (10.5)

© Bowe B et al. *JAMA Network Open*

location	YLD number		YLD per 100,000 persons		Age-standardized YLD per 100,000 persons									
					All causes		Due to diabetes		Due to hypertension		Due to glomerulonephritis		Due to other causes	
	2016 (UI)	Percent change from 2002	2016 (UI)	Percent change from 2002	2016 (UI)	Percent change from 2002	2016 (UI)	Percent change from 2002 (PA*)	2016 (UI)	Percent change from 2002 (PA*)	2016 (UI)	Percent change from 2002 (PA*)	2016 (UI)	Percent change from 2002 (PA*)
California	79894 (59378, 99831)	47.5	202 (150, 253)	30.7	164 (121, 204)	13.3	89 (65, 114)	15.9 (63.6)	28 (20, 37)	18.5 (22.8)	19 (14, 26)	6.1 (5.8)	27 (20, 36)	6 (7.9)
Colorado	10262 (7662, 12874)	66.5	186 (139, 234)	35.7	151 (112, 190)	16.3	83 (61, 105)	18.8 (61.8)	26 (19, 34)	18.6 (19.5)	17 (13, 23)	10.5 (7.8)	24 (17, 33)	10.3 (10.8)
Connecticut	8008 (5975, 10029)	34	224 (167, 280)	29	160 (120, 200)	17.8	88 (65, 112)	20.9 (62.5)	28 (19, 36)	20.5 (19.4)	19 (13, 25)	9.6 (6.8)	26 (18, 34)	11.8 (11.3)
Delaware	2353 (1754, 2977)	62	247 (184, 312)	37.1	176 (131, 222)	17.5	96 (71, 122)	20.2 (61.9)	30 (21, 40)	20.4 (19.7)	21 (15, 28)	11.3 (8)	29 (20, 38)	10.5 (10.4)
District of Columbia	1215 (909, 1531)	21.5	177 (133, 223)	2.9	157 (117, 198)	5.3	86 (63, 110)	8.3 (63.1)	27 (19, 36)	10.5 (25)	18 (13, 24)	-3.5 (6.5)	25 (18, 34)	-2.2 (5.4)
Florida	52387 (38940, 65904)	61.8	256 (190, 322)	31.9	168 (126, 212)	20.7	92 (68, 117)	23.4 (60.8)	29 (20, 38)	24 (19.6)	20 (14, 26)	14 (8.4)	27 (19, 37)	13.5 (11.3)
Georgia	22767 (16817, 28704)	62.4	221 (164, 279)	34.5	183 (136, 232)	15.7	100 (74, 128)	18.4 (62.7)	32 (22, 42)	19 (20.5)	21 (15, 29)	8.3 (6.6)	30 (21, 40)	9.3 (10.2)

© Bowe B et al. *JAMA Network Open*

location	YLD number		YLD per 100,000 persons		Age-standardized YLD per 100,000 persons									
					All causes		Due to diabetes		Due to hypertension		Due to glomerulonephritis		Due to other causes	
	2016 (UI)	Percent change from 2002	2016 (UI)	Percent change from 2002	2016 (UI)	Percent change from 2002	2016 (UI)	Percent change from 2002 (PA*)	2016 (UI)	Percent change from 2002 (PA*)	2016 (UI)	Percent change from 2002 (PA*)	2016 (UI)	Percent change from 2002 (PA*)
Hawaii	3372 (2514, 4261)	55.5	234 (175, 296)	34.6	168 (126, 213)	18.7	92 (68, 119)	21.1 (60.7)	29 (21, 38)	22.2 (20.1)	19 (14, 26)	11.7 (7.7)	27 (20, 36)	12.6 (11.6)
Idaho	3455 (2581, 4313)	72.6	206 (154, 257)	37.7	162 (122, 202)	20.7	89 (66, 112)	23.5 (60.6)	28 (20, 37)	23.5 (19.3)	19 (14, 25)	14.8 (8.8)	26 (18, 35)	13.6 (11.3)
Illinois	28139 (20787, 35543)	31.2	219 (162, 277)	28.1	168 (124, 211)	13.5	92 (68, 117)	16.1 (63.6)	29 (21, 39)	17.7 (22.2)	19 (14, 26)	6.2 (5.7)	27 (20, 36)	6.7 (8.5)
Indiana	15491 (11599, 19429)	41.3	234 (175, 293)	31.2	180 (135, 227)	18.5	98 (72, 125)	21.4 (61.5)	31 (22, 41)	20.7 (19.1)	21 (15, 28)	12.2 (8.1)	29 (21, 39)	12.1 (11.2)
Iowa	6936 (5176, 8662)	35.4	221 (165, 277)	26.6	161 (120, 202)	20	88 (65, 112)	22.5 (60.4)	28 (20, 36)	23.1 (19.6)	19 (13, 25)	14.5 (8.8)	26 (19, 35)	12.9 (11.1)
Kansas	6464 (4860, 8036)	36.6	222 (167, 276)	27.4	171 (129, 214)	18.5	94 (70, 119)	21.4 (61.8)	30 (21, 39)	21.4 (19.7)	20 (14, 27)	11.1 (7.4)	28 (20, 36)	11.9 (11.1)
Kentucky	10742 (7941, 13398)	43.9	243 (179, 303)	32.9	183 (137, 228)	19	100 (74, 126)	22.2 (62.1)	32 (23, 41)	21.1 (19)	21 (15, 29)	11.6 (7.7)	30 (21, 40)	12.3 (11.2)

© Bowe B et al. *JAMA Network Open*

location	YLD number		YLD per 100,000 persons		Age-standardized YLD per 100,000 persons									
					All causes		Due to diabetes		Due to hypertension		Due to glomerulonephritis		Due to other causes	
	2016 (UI)	Percent change from 2002	2016 (UI)	Percent change from 2002	2016 (UI)	Percent change from 2002	2016 (UI)	Percent change from 2002 (PA*)	2016 (UI)	Percent change from 2002 (PA*)	2016 (UI)	Percent change from 2002 (PA*)	2016 (UI)	Percent change from 2002 (PA*)
Louisiana	11311 (8372, 14323)	37.5	242 (179, 306)	32.2	192 (142, 245)	18.3	105 (78, 134)	21.1 (61.6)	33 (24, 44)	21.2 (19.6)	23 (16, 30)	11.6 (7.9)	31 (22, 42)	11.6 (10.9)
Maine	3386 (2526, 4273)	41.4	255 (190, 322)	37.6	165 (123, 209)	17.2	90 (67, 116)	19.5 (60.7)	29 (20, 37)	19.7 (19.5)	19 (14, 26)	10.5 (7.6)	27 (19, 35)	12.4 (12.2)
Maryland	13187 (9722, 16632)	44.9	219 (161, 276)	30.5	168 (124, 212)	15.4	92 (68, 119)	17.9 (62.4)	29 (21, 38)	19.7 (21.4)	20 (14, 26)	7.3 (5.9)	27 (20, 36)	9.3 (10.3)
Massachusetts	14950 (11053, 18898)	35.9	219 (162, 277)	27.5	159 (118, 200)	16.2	88 (65, 112)	19.2 (63.6)	28 (20, 37)	19.8 (20.8)	18 (13, 24)	8.5 (6.4)	26 (18, 34)	8.7 (9.2)
Michigan	23532 (17619, 29418)	35.1	237 (178, 297)	36.3	172 (129, 216)	16.9	94 (70, 121)	19.7 (62.4)	30 (22, 39)	19.4 (19.5)	20 (15, 27)	9.1 (6.7)	28 (20, 37)	11.3 (11.4)
Minnesota	11695 (8727, 14738)	44.5	212 (158, 268)	31.4	160 (119, 202)	16.7	87 (64, 112)	19.4 (62.3)	28 (20, 37)	19.5 (20)	19 (13, 25)	9.7 (7.2)	26 (18, 35)	10.2 (10.5)
Mississippi	7572 (5609, 9506)	39.2	253 (188, 318)	33.2	198 (147, 249)	18.6	108 (80, 139)	21.6 (62.1)	34 (24, 44)	22.2 (19.9)	23 (16, 30)	10.9 (7.4)	32 (23, 43)	11.4 (10.6)

© Bowe B et al. *JAMA Network Open*

location	YLD number		YLD per 100,000 persons		Age-standardized YLD per 100,000 persons									
					All causes		Due to diabetes		Due to hypertension		Due to glomerulonephritis		Due to other causes	
	2016 (UI)	Percent change from 2002	2016 (UI)	Percent change from 2002	2016 (UI)	Percent change from 2002	2016 (UI)	Percent change from 2002 (PA*)	2016 (UI)	Percent change from 2002 (PA*)	2016 (UI)	Percent change from 2002 (PA*)	2016 (UI)	Percent change from 2002 (PA*)
Missouri	14337 (10618, 17967)	42.5	236 (175, 295)	32.8	174 (129, 218)	20.3	95 (70, 121)	23.6 (62)	30 (21, 39)	22.5 (18.9)	20 (14, 27)	12.6 (7.7)	28 (20, 37)	13.4 (11.4)
Montana	2238 (1691, 2811)	50.2	216 (163, 271)	31.9	152 (115, 191)	17	83 (61, 106)	19.9 (62.6)	26 (19, 34)	19 (19.1)	18 (13, 23)	9.6 (7)	25 (18, 33)	11.1 (11.3)
Nebraska	4169 (3132, 5201)	39.8	219 (164, 273)	26.8	168 (126, 210)	19.7	92 (68, 118)	22.6 (61.2)	29 (21, 38)	22.2 (19.3)	20 (14, 26)	13.2 (8.3)	27 (20, 37)	12.8 (11.3)
Nevada	6448 (4823, 8139)	81.4	220 (165, 278)	34.1	171 (127, 215)	16.3	94 (69, 120)	18.8 (62)	30 (21, 39)	19 (19.9)	20 (14, 27)	10.4 (7.8)	28 (20, 36)	9.7 (10.3)
New Hampshire	3034 (2265, 3820)	48.7	227 (170, 286)	40.8	157 (117, 198)	16.3	86 (62, 109)	18.7 (61.1)	27 (19, 35)	19.4 (20.1)	18 (13, 25)	11.8 (8.8)	26 (18, 34)	9.4 (10)
New Jersey	20173 (14957, 25386)	34	225 (167, 283)	27.6	166 (123, 209)	15.8	91 (67, 117)	18.4 (62.5)	29 (21, 38)	19.3 (20.7)	19 (14, 26)	9.5 (7.4)	27 (19, 35)	8.7 (9.5)
New Mexico	4766 (3580, 5968)	58.9	230 (173, 288)	42.4	171 (129, 214)	20.8	94 (69, 120)	23.4 (60.1)	30 (21, 39)	23.3 (19.1)	20 (14, 27)	14.5 (8.6)	28 (20, 37)	15 (12.3)

© Bowe B et al. *JAMA Network Open*

location	YLD number		YLD per 100,000 persons		Age-standardized YLD per 100,000 persons									
					All causes		Due to diabetes		Due to hypertension		Due to glomerulonephritis		Due to other causes	
	2016 (UI)	Percent change from 2002	2016 (UI)	Percent change from 2002	2016 (UI)	Percent change from 2002	2016 (UI)	Percent change from 2002 (PA*)	2016 (UI)	Percent change from 2002 (PA*)	2016 (UI)	Percent change from 2002 (PA*)	2016 (UI)	Percent change from 2002 (PA*)
New York	43766 (32637, 55430)	36	221 (165, 280)	31.5	165 (123, 210)	19.1	90 (66, 116)	21.6 (60.5)	29 (20, 37)	22.1 (19.5)	19 (14, 26)	12.4 (8.1)	27 (19, 36)	13.3 (11.9)
North Carolina	24086 (18052, 30206)	63.6	238 (179, 299)	34.9	180 (134, 227)	18.7	99 (73, 126)	21.1 (60.8)	31 (22, 41)	21.5 (19.6)	21 (15, 28)	13.7 (8.8)	29 (21, 39)	11.7 (10.8)
North Dakota	1605 (1197, 2003)	40.9	208 (155, 260)	16.7	165 (123, 206)	19.6	90 (67, 115)	22.3 (61)	29 (20, 37)	21.5 (18.6)	19 (14, 26)	13.4 (8.4)	27 (19, 36)	13.6 (11.9)
Ohio	28167 (20993, 35445)	34.8	243 (181, 305)	32.4	177 (131, 223)	18.1	97 (71, 123)	21 (62)	31 (22, 40)	21.2 (19.8)	21 (15, 28)	10.6 (7.3)	29 (20, 38)	11.4 (10.9)
Oklahoma	8715 (6538, 10942)	44.5	223 (167, 280)	28.9	174 (132, 219)	21.3	95 (71, 121)	24.4 (61)	30 (21, 39)	23.9 (19)	21 (15, 27)	14 (8.2)	28 (20, 38)	14.4 (11.8)
Oregon	8782 (6579, 10987)	55.6	217 (163, 272)	35	156 (116, 195)	18.8	85 (63, 108)	21.1 (60.3)	27 (20, 36)	21.7 (19.8)	18 (13, 24)	14.1 (9.1)	25 (18, 33)	11.8 (10.8)
Pennsylvania	31091 (23018, 39196)	28.7	244 (180, 307)	24.1	169 (125, 214)	15.1	92 (68, 118)	17.5 (61.9)	29 (21, 39)	18.2 (20.4)	20 (14, 26)	8.7 (7.1)	27 (19, 36)	9.3 (10.5)

© Bowe B et al. *JAMA Network Open*

location	YLD number		YLD per 100,000 persons		Age-standardized YLD per 100,000 persons									
					All causes		Due to diabetes		Due to hypertension		Due to glomerulonephritis		Due to other causes	
	2016 (UI)	Percent change from 2002	2016 (UI)	Percent change from 2002	2016 (UI)	Percent change from 2002	2016 (UI)	Percent change from 2002 (PA*)	2016 (UI)	Percent change from 2002 (PA*)	2016 (UI)	Percent change from 2002 (PA*)	2016 (UI)	Percent change from 2002 (PA*)
Rhode Island	2359 (1760, 2944)	26.6	223 (167, 279)	27.5	159 (118, 199)	16.5	87 (64, 110)	19.3 (62.3)	28 (20, 36)	18.4 (19)	19 (13, 25)	10.2 (7.7)	26 (19, 35)	10.6 (11)
South Carolina	12263 (9115, 15543)	65.2	249 (185, 315)	37.4	183 (136, 231)	18.1	100 (74, 128)	20.9 (61.6)	32 (23, 42)	22.1 (20.6)	21 (15, 28)	11.1 (7.6)	30 (21, 39)	10.8 (10.2)
South Dakota	1874 (1406, 2359)	45	217 (162, 273)	27.5	160 (120, 203)	18.8	88 (65, 112)	21.4 (61)	28 (20, 36)	22 (19.9)	19 (13, 25)	11.1 (7.4)	26 (19, 35)	12.8 (11.7)
Tennessee	15513 (11554, 19573)	52.4	234 (174, 295)	33.3	176 (132, 221)	19	96 (71, 124)	22.5 (62.8)	31 (22, 40)	21.9 (19.5)	21 (15, 27)	10.7 (7.1)	29 (21, 38)	11.6 (10.6)
Texas	57200 (42901, 71101)	74.6	205 (154, 255)	36.3	179 (134, 223)	22.2	98 (72, 125)	24.9 (60)	31 (22, 41)	26 (19.8)	21 (15, 28)	14.9 (8.3)	29 (21, 38)	15.4 (11.9)
Utah	5163 (3888, 6495)	73.1	171 (128, 215)	33.8	167 (125, 210)	17.8	91 (68, 116)	20.2 (60.6)	29 (21, 38)	21.2 (20.3)	19 (14, 25)	11.5 (7.9)	27 (19, 36)	11.6 (11.2)
Vermont	1410 (1057, 1785)	39.2	226 (169, 286)	36.8	153 (115, 193)	15.6	84 (61, 108)	18.6 (63.5)	27 (19, 34)	18 (19.5)	18 (13, 24)	9.2 (7.3)	25 (18, 33)	8.7 (9.7)

© Bowe B et al. *JAMA Network Open*

location	YLD number		YLD per 100,000 persons		Age-standardized YLD per 100,000 persons									
					All causes		Due to diabetes		Due to hypertension		Due to glomerulonephritis		Due to other causes	
	2016 (UI)	Percent change from 2002	2016 (UI)	Percent change from 2002	2016 (UI)	Percent change from 2002	2016 (UI)	Percent change from 2002 (PA*)	2016 (UI)	Percent change from 2002 (PA*)	2016 (UI)	Percent change from 2002 (PA*)	2016 (UI)	Percent change from 2002 (PA*)
Virginia	18885 (13962, 23775)	53.2	224 (166, 282)	32.5	174 (129, 219)	17.5	95 (69, 121)	20.1 (61.6)	30 (22, 40)	19.7 (19.2)	20 (15, 27)	11.2 (7.9)	28 (20, 37)	11.6 (11.3)
Washington	14631 (10953, 18580)	63.7	203 (152, 258)	37.1	156 (117, 199)	20.8	85 (63, 109)	23.6 (60.6)	27 (19, 35)	24.2 (19.7)	18 (13, 24)	13 (7.8)	25 (18, 34)	14.5 (11.9)
West Virginia	5101 (3829, 6381)	35	279 (209, 348)	33	190 (142, 238)	21.7	104 (77, 131)	25 (61.1)	33 (23, 43)	23.7 (18.6)	23 (16, 30)	14.8 (8.6)	31 (22, 42)	14.6 (11.7)
Wisconsin	13188 (9856, 16428)	41.1	229 (171, 285)	32.8	165 (123, 207)	17.7	91 (67, 115)	20.8 (62.6)	29 (20, 38)	20.5 (19.8)	19 (14, 25)	9.8 (6.8)	27 (19, 36)	11.1 (10.8)
Wyoming	1138 (856, 1421)	50.4	195 (146, 243)	28.5	151 (114, 189)	17.1	83 (61, 105)	19.5 (61.2)	26 (19, 34)	20 (19.8)	18 (13, 23)	10.4 (7.5)	25 (18, 33)	11.5 (11.5)

YLD: Years Lost due to Disability
UI is the uncertainty interval, it reflects the certainty of an estimate based on data availability, studies size and consistency across data sources
*Percentage attributed to overall rate is the proportion of overall age-standardized rate change attributed by each of the following four causes: diabetes, hypertension, glomerulonephritis and other causes

© Bowe B et al. *JAMA Network Open*

eTable 4b: Number and age-standardized rate for overall YLD due to CKD and by cause in females, with percentage change from 2002 to 2016 for the Unites States and by state and district.

location	YLD number		YLD per 100,000 persons		Age-standardized YLD per 100,000 persons									
					All causes		Due to diabetes		Due to hypertension		Due to glomerulonephritis		Due to other causes	
	2016 (UI)	Percent change from 2002	2016 (UI)	Percent change from 2002	2016 (UI)	Percent change from 2002	2016 (UI)	Percent change from 2002 (PA*)	2016 (UI)	Percent change from 2002 (PA*)	2016 (UI)	Percent change from 2002 (PA*)	2016 (UI)	Percent change from 2002 (PA*)
United States	388224 (292199, 483021)	41.4	237 (178, 295)	26.3	171 (129, 212)	16.3	90 (67, 115)	18.8 (59.6)	30 (21, 39)	19.6 (20.5)	22 (16, 29)	9.9 (8.3)	29 (20, 38)	10.7 (11.5)
Alabama	6861 (5079, 8603)	37.1	274 (202, 343)	26.5	194 (144, 243)	17.4	102 (75, 132)	20.6 (60.8)	34 (23, 45)	20.5 (20.1)	25 (18, 34)	9.2 (7.4)	33 (23, 44)	11.5 (11.7)
Alaska	610 (455, 769)	54.7	176 (131, 221)	38.6	159 (119, 201)	15.9	84 (62, 107)	19.2 (62)	28 (20, 37)	18 (19.6)	21 (14, 28)	9.1 (7.9)	27 (18, 36)	9.3 (10.5)
Arizona	8117 (6072, 10088)	71	233 (175, 290)	32.9	166 (124, 208)	18	87 (64, 112)	20.2 (58.2)	29 (21, 38)	20.6 (19.8)	21 (15, 28)	12 (9.1)	28 (19, 37)	13.3 (12.9)
Arkansas	3877 (2857, 4911)	35.9	256 (189, 325)	24.2	182 (135, 229)	18.4	96 (70, 124)	21.7 (60.5)	32 (22, 42)	20.7 (19.3)	23 (17, 32)	11.6 (8.6)	30 (21, 41)	12.1 (11.6)
California	42301 (31756, 52787)	41	213 (160, 266)	24.5	164 (123, 205)	10.6	86 (64, 110)	12.7 (61.9)	28 (20, 37)	15.8 (24.6)	22 (15, 29)	3.8 (5.1)	28 (20, 37)	5.1 (8.4)

© Bowe B et al. *JAMA Network Open*

location	YLD number		YLD per 100,000 persons		Age-standardized YLD per 100,000 persons									
					All causes		Due to diabetes		Due to hypertension		Due to glomerulonephritis		Due to other causes	
	2016 (UI)	Percent change from 2002	2016 (UI)	Percent change from 2002	2016 (UI)	Percent change from 2002	2016 (UI)	Percent change from 2002 (PA*)	2016 (UI)	Percent change from 2002 (PA*)	2016 (UI)	Percent change from 2002 (PA*)	2016 (UI)	Percent change from 2002 (PA*)
Colorado	5326 (4001, 6681)	59.3	194 (146, 244)	29.5	150 (113, 189)	14.7	79 (59, 102)	16.6 (58.6)	26 (18, 34)	16.7 (19.7)	19 (13, 26)	10.7 (9.7)	25 (18, 34)	10.2 (12.1)
Connecticut	4318 (3238, 5410)	27.5	236 (177, 295)	23.4	159 (118, 200)	16	84 (61, 107)	18.7 (60.2)	28 (19, 36)	18.8 (20.1)	21 (15, 28)	9.1 (7.8)	27 (19, 36)	10.8 (11.9)
Delaware	1276 (954, 1613)	57.6	260 (194, 329)	33	177 (132, 225)	17.1	93 (69, 119)	19.7 (59.3)	31 (22, 41)	20.6 (20.5)	23 (16, 31)	11.3 (9)	30 (21, 40)	10.8 (11.2)
District of Columbia	682 (511, 864)	17.3	190 (142, 240)	0	159 (118, 200)	5.1	84 (62, 108)	8 (62.4)	28 (20, 37)	10.2 (25.8)	20 (14, 28)	-3.5 (7.5)	26 (18, 36)	-1.6 (4.3)
Florida	27913 (20921, 34836)	56.6	267 (200, 333)	27.9	168 (126, 211)	20	89 (66, 114)	22.6 (58.6)	29 (20, 38)	22.5 (19.2)	22 (15, 29)	14 (9.5)	28 (20, 38)	14.3 (12.6)
Georgia	12497 (9257, 15811)	56.5	237 (176, 300)	28.5	186 (138, 235)	14.3	98 (73, 126)	16.8 (61)	33 (23, 44)	17.7 (21.2)	24 (16, 32)	6.7 (6.5)	31 (21, 42)	9.3 (11.3)
Hawaii	1778 (1324, 2262)	52.6	251 (187, 319)	33.2	168 (125, 214)	16.8	88 (66, 115)	18.9 (58.5)	29 (21, 39)	20.4 (20.7)	22 (15, 29)	10.5 (8.5)	28 (20, 38)	11.8 (12.3)

© Bowe B et al. *JAMA Network Open*

location	YLD number		YLD per 100,000 persons		Age-standardized YLD per 100,000 persons									
					All causes		Due to diabetes		Due to hypertension		Due to glomerulonephritis		Due to other causes	
	2016 (UI)	Percent change from 2002	2016 (UI)	Percent change from 2002	2016 (UI)	Percent change from 2002	2016 (UI)	Percent change from 2002 (PA*)	2016 (UI)	Percent change from 2002 (PA*)	2016 (UI)	Percent change from 2002 (PA*)	2016 (UI)	Percent change from 2002 (PA*)
Idaho	1788 (1329, 2224)	66.8	214 (159, 266)	33.2	163 (121, 203)	20.5	86 (63, 109)	23.1 (58.3)	29 (20, 38)	22.8 (19.3)	21 (15, 28)	15.2 (10)	27 (19, 37)	14.4 (12.5)
Illinois	15120 (11262, 19074)	25	231 (172, 292)	22.3	167 (125, 211)	12	88 (65, 112)	14.4 (61.8)	30 (21, 39)	16.2 (23.1)	21 (15, 29)	4.5 (5.2)	28 (20, 37)	6.9 (10)
Indiana	8368 (6196, 10442)	34.2	249 (184, 311)	24.9	181 (135, 227)	16.7	95 (71, 122)	19 (58.8)	32 (22, 43)	20 (20.4)	23 (16, 31)	10.9 (8.8)	30 (21, 40)	11.4 (11.9)
Iowa	3718 (2757, 4637)	27.8	236 (175, 294)	20.8	161 (119, 202)	18.6	85 (62, 109)	19.9 (55.8)	28 (20, 37)	21.5 (20)	21 (14, 28)	15.7 (11.1)	27 (18, 36)	14 (13.1)
Kansas	3441 (2603, 4353)	29	236 (179, 299)	21.3	171 (128, 217)	16.6	90 (68, 116)	19.7 (61)	30 (21, 40)	19.5 (20.2)	22 (15, 30)	8.4 (7)	29 (20, 39)	11.2 (11.8)
Kentucky	5801 (4308, 7282)	37.6	258 (192, 324)	27.8	184 (137, 233)	18.2	97 (71, 125)	21.1 (59.7)	32 (23, 42)	20.7 (19.6)	24 (17, 32)	11.7 (8.8)	31 (22, 41)	12.2 (11.9)
Louisiana	6228 (4621, 7945)	32.6	260 (193, 332)	28.5	197 (147, 250)	18.6	104 (77, 135)	21.1 (59)	34 (24, 45)	21.6 (19.8)	25 (18, 34)	12.7 (9.3)	33 (23, 45)	12.6 (11.9)

© Bowe B et al. *JAMA Network Open*

location	YLD number		YLD per 100,000 persons		Age-standardized YLD per 100,000 persons									
					All causes		Due to diabetes		Due to hypertension		Due to glomerulonephritis		Due to other causes	
	2016 (UI)	Percent change from 2002	2016 (UI)	Percent change from 2002	2016 (UI)	Percent change from 2002	2016 (UI)	Percent change from 2002 (PA*)	2016 (UI)	Percent change from 2002 (PA*)	2016 (UI)	Percent change from 2002 (PA*)	2016 (UI)	Percent change from 2002 (PA*)
Maine	1804 (1342, 2281)	33.5	266 (198, 337)	30.5	165 (123, 210)	15.3	87 (64, 112)	17.2 (58.3)	29 (20, 38)	18.1 (20.2)	21 (15, 29)	10.1 (9)	28 (20, 37)	11 (12.5)
Maryland	7168 (5245, 9104)	40.9	231 (169, 293)	27.4	167 (123, 212)	15	89 (65, 114)	17.5 (60.5)	29 (20, 39)	18.4 (20.8)	22 (15, 29)	8.2 (7.5)	28 (19, 37)	9.6 (11.2)
Massachusetts	7847 (5773, 9983)	28.2	223 (164, 284)	20.9	153 (113, 193)	13.7	81 (60, 104)	17 (63.6)	27 (19, 36)	17.6 (22)	19 (13, 26)	6.2 (6.1)	25 (18, 35)	6.4 (8.2)
Michigan	12601 (9437, 15775)	28.9	250 (187, 313)	30.2	172 (128, 218)	15.1	91 (67, 117)	17.5 (60.2)	30 (22, 40)	17.1 (19.5)	22 (16, 30)	7.5 (6.8)	29 (20, 39)	11.8 (13.5)
Minnesota	6154 (4597, 7791)	37.2	222 (166, 281)	25.2	159 (118, 201)	15	83 (62, 108)	18 (61.4)	28 (20, 37)	18.8 (21.6)	20 (15, 28)	7.8 (7.1)	27 (19, 36)	8.4 (9.9)
Mississippi	4243 (3152, 5381)	34	275 (205, 349)	28.3	204 (152, 259)	17.8	108 (79, 140)	20.7 (60.3)	35 (25, 47)	21.5 (20.3)	27 (18, 35)	9.8 (7.7)	34 (24, 47)	11.8 (11.8)
Missouri	7749 (5729, 9730)	36.4	250 (185, 314)	27.9	174 (129, 221)	19.8	92 (68, 119)	23.3 (60.5)	31 (22, 40)	21.6 (18.9)	22 (16, 30)	11.9 (8.2)	29 (21, 39)	14 (12.4)

© Bowe B et al. *JAMA Network Open*

location	YLD number		YLD per 100,000 persons		Age-standardized YLD per 100,000 persons									
					All causes		Due to diabetes		Due to hypertension		Due to glomerulonephritis		Due to other causes	
	2016 (UI)	Percent change from 2002	2016 (UI)	Percent change from 2002	2016 (UI)	Percent change from 2002	2016 (UI)	Percent change from 2002 (PA*)	2016 (UI)	Percent change from 2002 (PA*)	2016 (UI)	Percent change from 2002 (PA*)	2016 (UI)	Percent change from 2002 (PA*)
Montana	1152 (865, 1451)	41.3	224 (168, 282)	25.1	151 (114, 191)	14.5	80 (59, 102)	17.4 (61.8)	27 (19, 35)	17.4 (20.6)	19 (14, 26)	8.2 (7.7)	25 (18, 34)	8.2 (10)
Nebraska	2216 (1656, 2768)	31.3	232 (174, 290)	20.4	169 (127, 211)	17.3	89 (65, 114)	20.2 (59.8)	30 (21, 39)	20.3 (20.2)	22 (15, 29)	11.2 (8.7)	28 (20, 38)	11 (11.3)
Nevada	3281 (2432, 4161)	79.4	224 (166, 284)	30.8	170 (126, 215)	15.9	90 (66, 115)	18.2 (59.4)	30 (21, 39)	18.2 (19.8)	22 (15, 29)	10.9 (9.2)	28 (20, 38)	10.5 (11.6)
New Hampshire	1594 (1198, 2010)	41.9	236 (178, 298)	34.7	156 (117, 197)	14.8	82 (61, 105)	16.8 (58.4)	27 (19, 36)	17.8 (20.5)	20 (14, 28)	11.2 (10.2)	26 (18, 35)	9.1 (10.9)
New Jersey	10922 (8038, 13780)	28.4	238 (175, 301)	23	165 (121, 208)	14.5	87 (64, 112)	17.1 (61.1)	29 (20, 38)	18.1 (21.4)	21 (15, 29)	8.3 (7.8)	28 (19, 37)	7.8 (9.6)
New Mexico	2521 (1877, 3180)	53.6	241 (180, 304)	38.6	172 (129, 216)	19.8	91 (67, 117)	22.4 (58.8)	30 (21, 40)	21.7 (19)	22 (15, 29)	14.9 (10.1)	29 (20, 38)	13.7 (12.2)
New York	23821 (17799, 29969)	30.1	234 (175, 294)	26.6	165 (124, 209)	18	87 (64, 111)	20.1 (57.9)	29 (20, 38)	21.4 (20.3)	21 (15, 29)	12.5 (9.5)	28 (19, 37)	12.6 (12.3)

© Bowe B et al. *JAMA Network Open*

location	YLD number		YLD per 100,000 persons		Age-standardized YLD per 100,000 persons									
					All causes		Due to diabetes		Due to hypertension		Due to glomerulonephritis		Due to other causes	
	2016 (UI)	Percent change from 2002	2016 (UI)	Percent change from 2002	2016 (UI)	Percent change from 2002	2016 (UI)	Percent change from 2002 (PA*)	2016 (UI)	Percent change from 2002 (PA*)	2016 (UI)	Percent change from 2002 (PA*)	2016 (UI)	Percent change from 2002 (PA*)
North Carolina	13255 (9936, 16668)	58	256 (192, 321)	29.5	183 (137, 232)	18.1	97 (72, 124)	20 (57.5)	32 (23, 43)	20.1 (19.2)	23 (16, 32)	15.3 (11.1)	31 (22, 41)	12.6 (12.2)
North Dakota	833 (624, 1050)	31.9	222 (167, 280)	12.3	165 (123, 207)	17.7	87 (63, 111)	19.9 (57.9)	29 (20, 38)	19.3 (18.8)	21 (15, 29)	12.2 (9.4)	28 (20, 37)	14.1 (13.9)
Ohio	15248 (11288, 19228)	28.3	258 (191, 325)	27	177 (132, 225)	16.6	94 (69, 120)	19.6 (61)	31 (22, 42)	19.9 (20.5)	23 (16, 31)	9.5 (7.9)	30 (20, 40)	10 (10.7)
Oklahoma	4664 (3483, 5854)	38.1	236 (176, 296)	23.7	175 (132, 220)	20.3	92 (69, 118)	23.1 (58.6)	31 (22, 41)	23.5 (19.8)	23 (16, 31)	14.2 (9.5)	29 (21, 40)	13.8 (12.1)
Oregon	4644 (3461, 5830)	48.9	228 (170, 286)	29	156 (116, 196)	17.4	82 (60, 105)	18.8 (56.1)	28 (19, 36)	20.4 (20.3)	20 (14, 27)	14.4 (10.9)	26 (18, 35)	12.8 (12.7)
Pennsylvania	16849 (12607, 21262)	22.2	258 (193, 326)	19	168 (125, 213)	13.8	89 (65, 113)	16.1 (60.1)	30 (21, 39)	17.2 (21.3)	22 (15, 29)	8.1 (8)	28 (20, 38)	8.3 (10.6)
Rhode Island	1278 (956, 1602)	19.7	235 (176, 295)	21.3	157 (117, 198)	14.4	82 (60, 105)	16.8 (60.1)	27 (19, 36)	16.5 (19.7)	20 (14, 28)	8.5 (8.1)	26 (19, 35)	9.9 (12.1)

© Bowe B et al. *JAMA Network Open*

location	YLD number		YLD per 100,000 persons		Age-standardized YLD per 100,000 persons									
					All causes		Due to diabetes		Due to hypertension		Due to glomerulonephritis		Due to other causes	
	2016 (UI)	Percent change from 2002	2016 (UI)	Percent change from 2002	2016 (UI)	Percent change from 2002	2016 (UI)	Percent change from 2002 (PA*)	2016 (UI)	Percent change from 2002 (PA*)	2016 (UI)	Percent change from 2002 (PA*)	2016 (UI)	Percent change from 2002 (PA*)
South Carolina	6706 (4992, 8428)	58.6	264 (197, 332)	31.9	186 (139, 235)	17.5	98 (71, 126)	20.2 (59.7)	33 (23, 44)	21.6 (21)	24 (17, 32)	10.3 (8)	31 (22, 42)	11.2 (11.3)
South Dakota	989 (747, 1246)	37.4	230 (174, 290)	22.4	161 (120, 206)	17.9	85 (63, 109)	20.3 (58.7)	28 (20, 38)	21.3 (20.4)	21 (14, 27)	10.4 (8)	27 (19, 37)	13.3 (12.9)
Tennessee	8456 (6272, 10627)	45.4	249 (185, 313)	27.3	179 (134, 225)	18	94 (69, 120)	21.5 (61.4)	31 (22, 41)	19.9 (19)	23 (16, 31)	10.2 (7.9)	30 (22, 40)	11.9 (11.7)
Texas	30701 (23126, 38327)	68.7	219 (165, 274)	31.7	181 (136, 227)	21.3	96 (71, 121)	23.7 (57.6)	32 (22, 42)	24.8 (20)	23 (16, 31)	15.8 (10)	30 (21, 40)	15 (12.4)
Utah	2650 (1989, 3330)	66.2	176 (132, 221)	28.7	165 (124, 208)	16.3	87 (65, 111)	17.5 (55.8)	30 (21, 39)	20.5 (21.8)	21 (15, 28)	12.9 (10.5)	28 (20, 38)	11 (11.9)
Vermont	752 (559, 960)	31.6	237 (177, 303)	29.6	154 (116, 198)	13.3	81 (60, 105)	16.2 (62.7)	27 (19, 36)	15.1 (19.6)	20 (14, 27)	8.1 (8.3)	26 (19, 35)	7 (9.4)
Virginia	10143 (7577, 12846)	47.1	237 (177, 301)	27.7	175 (130, 221)	16.4	92 (67, 118)	19.2 (60.4)	31 (22, 41)	17.5 (18.6)	22 (16, 30)	10.2 (8.4)	29 (20, 39)	11.9 (12.6)

© Bowe B et al. *JAMA Network Open*

location	YLD number		YLD per 100,000 persons		Age-standardized YLD per 100,000 persons									
					All causes		Due to diabetes		Due to hypertension		Due to glomerulonephritis		Due to other causes	
	2016 (UI)	Percent change from 2002	2016 (UI)	Percent change from 2002	2016 (UI)	Percent change from 2002	2016 (UI)	Percent change from 2002 (PA*)	2016 (UI)	Percent change from 2002 (PA*)	2016 (UI)	Percent change from 2002 (PA*)	2016 (UI)	Percent change from 2002 (PA*)
Washington	7673 (5797, 9686)	56.6	213 (161, 269)	31.7	156 (117, 197)	19.3	82 (60, 106)	21.8 (58.3)	28 (20, 37)	23 (20.4)	20 (14, 27)	12.3 (8.7)	26 (19, 36)	13.8 (12.6)
West Virginia	2730 (2039, 3423)	27	295 (220, 369)	26.4	193 (145, 243)	20.5	102 (75, 130)	23.3 (58.6)	34 (24, 45)	22.8 (19)	25 (17, 34)	14.8 (9.9)	32 (23, 43)	14.4 (12.5)
Wisconsin	6995 (5243, 8805)	33.1	241 (181, 303)	25.9	165 (124, 208)	15.5	87 (65, 112)	18.2 (60.7)	29 (21, 39)	18.1 (20.2)	21 (15, 28)	8.8 (7.7)	28 (19, 37)	10 (11.4)
Wyoming	570 (425, 720)	40.6	199 (149, 252)	21.7	150 (112, 190)	15	79 (58, 101)	17.3 (59.7)	26 (18, 35)	17.9 (20.5)	19 (14, 26)	9.4 (8.6)	25 (18, 33)	9.6 (11.2)

YLD: Years Lost due to Disability
UI is the uncertainty interval, it reflects the certainty of an estimate based on data availability, studies size and consistency across data sources
*Percentage attributed to overall rate is the proportion of overall age-standardized rate change attributed by each of the following four causes: diabetes, hypertension, glomerulonephritis and other causes

© Bowe B et al. *JAMA Network Open*

eTable 4c: Number and age-standardized rate for overall YLD due to CKD and by cause in males, with percentage change from 2002 to 2016 for the Unites States and by state and district.

location	YLD number		YLD per 100,000 persons		Age-standardized YLD per 100,000 persons									
					All causes		Due to diabetes		Due to hypertension		Due to glomerulonephritis		Due to other causes	
	2016 (UI)	Percent change from 2002	2016 (UI)	Percent change from 2002	2016 (UI)	Percent change from 2002	2016 (UI)	Percent change from 2002 (PA*)	2016 (UI)	Percent change from 2002 (PA*)	2016 (UI)	Percent change from 2002 (PA*)	2016 (UI)	Percent change from 2002 (PA*)
United States	335224 (249762, 423072)	55.9	211 (157, 266)	38.5	170 (127, 213)	19.3	97 (71, 123)	21.9 (63.1)	29 (20, 38)	23.3 (19.9)	18 (13, 24)	12.1 (6.9)	27 (19, 35)	11.7 (10.2)
Alabama	5544 (4077, 7093)	49.9	236 (173, 301)	38.1	187 (138, 238)	18.8	106 (78, 137)	21.5 (63.7)	32 (22, 42)	22.2 (19.5)	20 (13, 27)	12.9 (7.6)	29 (21, 39)	10.2 (9.2)
Alaska	635 (475, 803)	73.4	164 (123, 207)	49.7	158 (118, 198)	22.2	90 (66, 116)	25.2 (63.1)	27 (19, 36)	24.4 (18.5)	16 (12, 22)	16.2 (8)	25 (17, 33)	13.8 (10.4)
Arizona	7297 (5437, 9109)	82.5	213 (158, 265)	43.3	164 (122, 204)	19.9	93 (69, 117)	23 (64.1)	28 (20, 37)	23 (19.3)	17 (12, 23)	10.1 (5.7)	26 (18, 34)	13.2 (10.9)
Arkansas	3285 (2417, 4164)	50.1	225 (165, 285)	35.8	177 (129, 223)	20.7	101 (73, 129)	23.9 (64.1)	30 (21, 40)	24.5 (19.6)	19 (13, 26)	11.9 (6.5)	28 (19, 37)	12 (9.8)
California	37593 (27578, 47157)	55.7	192 (141, 241)	38.3	164 (121, 206)	16.5	93 (68, 119)	19 (64.2)	28 (20, 37)	22.3 (21.8)	17 (12, 23)	9.3 (6.3)	26 (18, 35)	7.4 (7.6)

© Bowe B et al. *JAMA Network Open*

location	YLD number		YLD per 100,000 persons		Age-standardized YLD per 100,000 persons									
					All causes		Due to diabetes		Due to hypertension		Due to glomerulonephritis		Due to other causes	
	2016 (UI)	Percent change from 2002	2016 (UI)	Percent change from 2002	2016 (UI)	Percent change from 2002	2016 (UI)	Percent change from 2002 (PA*)	2016 (UI)	Percent change from 2002 (PA*)	2016 (UI)	Percent change from 2002 (PA*)	2016 (UI)	Percent change from 2002 (PA*)
Colorado	4936 (3650, 6262)	75	178 (132, 226)	43	152 (112, 193)	18.3	86 (63, 111)	21 (63.9)	26 (18, 34)	21.3 (19.4)	16 (11, 22)	11 (6.6)	24 (17, 32)	11.1 (10.2)
Connecticut	3690 (2748, 4718)	42.5	211 (157, 270)	36.5	162 (120, 206)	20.1	92 (67, 119)	22.9 (63.8)	28 (19, 36)	23.1 (19.1)	17 (12, 22)	10.9 (6.1)	25 (17, 34)	13.5 (11)
Delaware	1076 (788, 1361)	67.6	233 (171, 295)	42.1	174 (128, 221)	17.9	100 (71, 126)	20.6 (64.3)	30 (21, 39)	20.4 (19)	18 (12, 25)	11.6 (7.1)	27 (19, 37)	10.4 (9.7)
District of Columbia	533 (393, 680)	27.3	164 (121, 209)	7	156 (115, 199)	5.9	89 (64, 114)	8.5 (64.4)	27 (19, 36)	11.4 (25.4)	16 (11, 22)	-3.1 (4.7)	24 (17, 33)	-2.4 (5.6)
Florida	24475 (18202, 31046)	68	245 (182, 311)	36.9	168 (124, 214)	21.6	96 (70, 123)	24.3 (62.6)	29 (20, 38)	26.2 (20)	17 (12, 24)	14.5 (7.4)	26 (18, 35)	13 (10.1)
Georgia	10270 (7564, 13051)	70.2	205 (151, 261)	42.3	181 (133, 229)	17.6	103 (75, 133)	20 (63.5)	31 (22, 41)	21.5 (20.2)	19 (13, 25)	11.1 (6.9)	28 (20, 39)	9.9 (9.4)
Hawaii	1594 (1181, 1998)	58.8	218 (162, 273)	36.3	170 (125, 213)	21	97 (71, 124)	23.4 (62.1)	29 (20, 39)	24.6 (19.4)	18 (12, 24)	14.2 (7.4)	27 (18, 36)	13.9 (11)

© Bowe B et al. *JAMA Network Open*

location	YLD number		YLD per 100,000 persons		Age-standardized YLD per 100,000 persons									
					All causes		Due to diabetes		Due to hypertension		Due to glomerulonephritis		Due to other causes	
	2016 (UI)	Percent change from 2002	2016 (UI)	Percent change from 2002	2016 (UI)	Percent change from 2002	2016 (UI)	Percent change from 2002 (PA*)	2016 (UI)	Percent change from 2002 (PA*)	2016 (UI)	Percent change from 2002 (PA*)	2016 (UI)	Percent change from 2002 (PA*)
Idaho	1667 (1232, 2091)	79.2	198 (147, 249)	43	162 (122, 203)	21.2	92 (67, 117)	23.8 (62.3)	28 (20, 36)	24.9 (19.5)	17 (12, 23)	14.9 (7.8)	25 (18, 34)	13.3 (10.5)
Illinois	13019 (9633, 16356)	39.2	206 (153, 259)	35.7	169 (125, 213)	15.3	96 (71, 123)	17.6 (64.2)	29 (20, 38)	20 (21.6)	17 (12, 24)	9.1 (6.5)	26 (19, 35)	7 (7.7)
Indiana	7123 (5325, 9042)	50.7	218 (163, 277)	39.4	179 (133, 227)	20.8	102 (74, 130)	23.7 (63.3)	31 (22, 40)	22.3 (18.1)	19 (13, 25)	14.7 (7.8)	28 (20, 38)	13.4 (10.8)
Iowa	3218 (2413, 4128)	45.3	207 (155, 265)	34.5	161 (121, 205)	21.7	92 (68, 118)	25 (63.7)	27 (20, 36)	25.8 (19.6)	17 (12, 23)	13.8 (7.1)	25 (18, 34)	12.3 (9.6)
Kansas	3023 (2256, 3806)	46.3	209 (156, 263)	35.4	172 (128, 217)	20.7	97 (71, 124)	22.9 (61.8)	29 (21, 39)	24.1 (19.4)	18 (13, 25)	15.3 (8.1)	27 (19, 35)	13.4 (10.8)
Kentucky	4941 (3676, 6228)	52	227 (169, 286)	39.7	182 (135, 228)	20	103 (74, 131)	23.1 (63.7)	31 (22, 41)	22 (18.5)	19 (14, 26)	12.6 (7)	29 (20, 38)	12.9 (10.8)
Louisiana	5083 (3718, 6526)	43.9	222 (162, 285)	37.3	188 (137, 239)	18.1	107 (77, 136)	20.9 (64)	32 (23, 43)	21.2 (19.5)	19 (13, 26)	10.7 (6.5)	30 (21, 40)	10.8 (10)

© Bowe B et al. *JAMA Network Open*

location	YLD number		YLD per 100,000 persons		Age-standardized YLD per 100,000 persons									
					All causes		Due to diabetes		Due to hypertension		Due to glomerulonephritis		Due to other causes	
	2016 (UI)	Percent change from 2002	2016 (UI)	Percent change from 2002	2016 (UI)	Percent change from 2002	2016 (UI)	Percent change from 2002 (PA*)	2016 (UI)	Percent change from 2002 (PA*)	2016 (UI)	Percent change from 2002 (PA*)	2016 (UI)	Percent change from 2002 (PA*)
Maine	1582 (1182, 2009)	51.7	243 (182, 309)	46.8	165 (123, 209)	19.4	93 (68, 120)	21.7 (62)	28 (20, 37)	22.1 (19)	17 (12, 24)	11.7 (6.7)	26 (18, 35)	14.4 (12.2)
Maryland	6019 (4443, 7677)	49.9	206 (152, 263)	34.5	169 (125, 215)	16.1	97 (70, 125)	18.2 (63.4)	29 (20, 38)	21.9 (22)	17 (12, 24)	6.9 (4.8)	26 (18, 36)	9.6 (9.9)
Massachusetts	7103 (5235, 9034)	45.7	214 (158, 272)	35.9	167 (123, 211)	18.7	95 (70, 123)	21 (63)	29 (20, 38)	22.4 (20.2)	17 (12, 23)	12 (6.8)	26 (18, 35)	11.4 (10)
Michigan	10930 (8105, 13788)	43.1	224 (166, 283)	44.2	173 (129, 218)	19.1	98 (72, 126)	21.9 (63.5)	30 (21, 39)	22.9 (19.8)	18 (13, 24)	11.8 (6.9)	27 (19, 36)	11.2 (9.8)
Minnesota	5541 (4073, 7085)	53.4	202 (149, 259)	39.1	161 (118, 206)	18.2	92 (66, 119)	20.4 (62.5)	27 (19, 37)	20.2 (18.5)	17 (12, 23)	12.7 (7.6)	25 (18, 34)	12.6 (11.4)
Mississippi	3330 (2471, 4262)	46.6	230 (171, 294)	40	191 (142, 244)	19.7	109 (79, 141)	22.3 (63.3)	32 (23, 43)	23.6 (19.6)	20 (14, 27)	13.2 (7.4)	30 (21, 40)	11.3 (9.6)
Missouri	6588 (4860, 8299)	50.4	221 (163, 278)	39.2	173 (128, 218)	20.9	98 (72, 125)	23.7 (62.9)	30 (21, 39)	24.2 (19.2)	18 (12, 25)	14 (7.3)	27 (19, 37)	13.2 (10.6)

© Bowe B et al. *JAMA Network Open*

location	YLD number		YLD per 100,000 persons		Age-standardized YLD per 100,000 persons									
					All causes		Due to diabetes		Due to hypertension		Due to glomerulonephritis		Due to other causes	
	2016 (UI)	Percent change from 2002	2016 (UI)	Percent change from 2002	2016 (UI)	Percent change from 2002	2016 (UI)	Percent change from 2002 (PA*)	2016 (UI)	Percent change from 2002 (PA*)	2016 (UI)	Percent change from 2002 (PA*)	2016 (UI)	Percent change from 2002 (PA*)
Montana	1086 (810, 1380)	60.8	208 (155, 265)	40.1	152 (114, 191)	19.7	86 (63, 109)	22.2 (62.6)	26 (18, 34)	21.2 (18.1)	16 (11, 22)	12 (6.9)	24 (17, 32)	14.9 (12.5)
Nebraska	1953 (1452, 2463)	50.8	205 (153, 259)	35.3	169 (126, 213)	22.3	96 (71, 122)	24.7 (61.7)	29 (20, 38)	25.1 (18.8)	18 (12, 24)	16.6 (8.1)	26 (18, 36)	15.3 (11.4)
Nevada	3168 (2350, 4028)	83.5	216 (160, 274)	37.5	173 (127, 218)	16.8	98 (72, 125)	19.5 (64.3)	30 (21, 39)	20.3 (20.1)	18 (12, 24)	9.9 (6.5)	27 (19, 36)	9.1 (9.1)
New Hampshire	1439 (1069, 1854)	57.2	218 (162, 281)	48.4	158 (117, 202)	18.1	90 (65, 116)	20.4 (62.7)	27 (19, 35)	21.7 (19.9)	17 (12, 23)	13.2 (8)	25 (18, 34)	10.1 (9.5)
New Jersey	9251 (6845, 11659)	41.2	211 (156, 266)	33.7	168 (125, 210)	17.4	96 (70, 123)	19.4 (62.8)	29 (20, 38)	21.1 (20.1)	17 (12, 23)	12.2 (7.5)	26 (18, 35)	10.1 (9.6)
New Mexico	2245 (1687, 2839)	65.3	218 (164, 276)	47	171 (129, 216)	22.3	97 (71, 123)	24.4 (60.7)	29 (21, 39)	25.7 (19.3)	18 (13, 24)	14.9 (7.4)	27 (19, 36)	17 (12.5)
New York	19946 (14828, 25647)	43.8	207 (154, 266)	38.1	166 (124, 213)	20.5	95 (69, 122)	22.9 (62.4)	28 (20, 37)	23.4 (18.9)	17 (12, 23)	13 (6.9)	26 (18, 35)	14.7 (11.7)

© Bowe B et al. *JAMA Network Open*

location	YLD number		YLD per 100,000 persons		Age-standardized YLD per 100,000 persons									
					All causes		Due to diabetes		Due to hypertension		Due to glomerulonephritis		Due to other causes	
	2016 (UI)	Percent change from 2002	2016 (UI)	Percent change from 2002	2016 (UI)	Percent change from 2002	2016 (UI)	Percent change from 2002 (PA*)	2016 (UI)	Percent change from 2002 (PA*)	2016 (UI)	Percent change from 2002 (PA*)	2016 (UI)	Percent change from 2002 (PA*)
North Carolina	10832 (7995, 13706)	71.1	220 (163, 279)	41.9	178 (131, 225)	19.6	102 (74, 130)	22.3 (63.6)	30 (21, 39)	24.1 (20.2)	18 (12, 25)	12 (6.7)	28 (19, 37)	11.2 (9.5)
North Dakota	772 (575, 968)	52.1	195 (145, 245)	22.7	166 (124, 209)	21.7	94 (70, 120)	24.4 (62.6)	28 (20, 37)	24.3 (18.6)	17 (13, 24)	16 (8.1)	26 (18, 35)	13.8 (10.6)
Ohio	12919 (9594, 16354)	43.2	227 (169, 287)	39.7	176 (130, 222)	19.8	100 (74, 128)	22.1 (62.3)	30 (21, 40)	23.2 (19.4)	18 (13, 25)	12.8 (7.1)	28 (19, 37)	13.4 (11.3)
Oklahoma	4051 (3032, 5134)	52.8	209 (156, 265)	35.6	174 (129, 220)	22.5	98 (72, 126)	25.5 (62.7)	30 (21, 39)	24.8 (18.4)	18 (13, 25)	14.3 (7.2)	27 (19, 37)	15.7 (11.7)
Oregon	4138 (3090, 5274)	63.8	207 (154, 263)	42.3	156 (115, 197)	20.5	88 (65, 113)	23.4 (63.6)	27 (19, 35)	23.9 (19.5)	16 (11, 22)	14.2 (7.6)	24 (17, 32)	11.3 (9.4)
Pennsylvania	14241 (10508, 18066)	37.3	228 (168, 289)	30.9	169 (125, 214)	16.6	96 (70, 123)	18.7 (62.6)	29 (20, 39)	19.7 (19.8)	18 (12, 24)	10.4 (6.8)	27 (18, 36)	10.8 (10.8)
Rhode Island	1081 (796, 1369)	35.9	211 (156, 268)	35.9	162 (119, 205)	18.9	92 (67, 117)	21.6 (63.5)	28 (19, 36)	21 (18.7)	17 (12, 23)	13.1 (7.6)	25 (18, 35)	11.5 (10.3)

© Bowe B et al. *JAMA Network Open*

location	YLD number		YLD per 100,000 persons		Age-standardized YLD per 100,000 persons									
					All causes		Due to diabetes		Due to hypertension		Due to glomerulonephritis		Due to other causes	
	2016 (UI)	Percent change from 2002	2016 (UI)	Percent change from 2002	2016 (UI)	Percent change from 2002	2016 (UI)	Percent change from 2002 (PA*)	2016 (UI)	Percent change from 2002 (PA*)	2016 (UI)	Percent change from 2002 (PA*)	2016 (UI)	Percent change from 2002 (PA*)
South Carolina	5557 (4086, 7110)	74	232 (170, 297)	44.7	180 (132, 230)	19.1	102 (75, 132)	21.6 (62.9)	31 (21, 41)	23.4 (20.3)	19 (13, 25)	12.9 (7.4)	28 (20, 38)	10.8 (9.4)
South Dakota	885 (660, 1119)	54.6	203 (152, 257)	34.2	160 (119, 203)	19.9	90 (67, 117)	22.4 (62.6)	27 (19, 36)	23.2 (19.4)	17 (12, 23)	12.7 (7.1)	25 (18, 34)	12.9 (10.9)
Tennessee	7057 (5185, 8981)	61.8	218 (160, 278)	41.4	174 (128, 220)	20.7	99 (72, 127)	23.7 (63.4)	30 (21, 39)	25.3 (20.1)	18 (12, 25)	12.2 (6.6)	27 (19, 37)	12.2 (9.9)
Texas	26499 (19811, 33530)	82.1	192 (143, 242)	42.2	177 (132, 223)	23.5	101 (74, 130)	26 (61.9)	30 (21, 40)	28.1 (19.7)	18 (13, 25)	14.4 (6.8)	28 (19, 37)	16.5 (11.6)
Utah	2514 (1893, 3168)	81	165 (124, 208)	39.7	169 (127, 213)	19.5	96 (71, 122)	22.6 (64.2)	29 (21, 38)	22.3 (19.1)	17 (12, 23)	10.4 (5.9)	26 (19, 36)	12.6 (10.7)
Vermont	658 (490, 831)	49.1	214 (159, 270)	46.2	152 (112, 191)	18.6	87 (63, 111)	21.1 (63.1)	26 (18, 34)	22.5 (19.9)	16 (11, 21)	11.3 (6.8)	24 (17, 32)	11.4 (10.3)
Virginia	8743 (6382, 11072)	61	211 (154, 267)	38.7	174 (127, 220)	18.9	99 (71, 127)	20.9 (61.9)	30 (21, 39)	23.1 (20.1)	18 (13, 25)	13.4 (7.7)	27 (19, 36)	11.8 (10.3)

© Bowe B et al. *JAMA Network Open*

location	YLD number		YLD per 100,000 persons		Age-standardized YLD per 100,000 persons									
					All causes		Due to diabetes		Due to hypertension		Due to glomerulonephritis		Due to other causes	
	2016 (UI)	Percent change from 2002	2016 (UI)	Percent change from 2002	2016 (UI)	Percent change from 2002	2016 (UI)	Percent change from 2002 (PA*)	2016 (UI)	Percent change from 2002 (PA*)	2016 (UI)	Percent change from 2002 (PA*)	2016 (UI)	Percent change from 2002 (PA*)
Washington	6959 (5183, 8916)	72.4	193 (143, 247)	43.7	156 (116, 200)	22.8	89 (65, 115)	25.4 (62.1)	27 (19, 35)	26.3 (19.2)	16 (11, 22)	14.8 (7.2)	24 (17, 33)	15.9 (11.6)
West Virginia	2371 (1755, 3008)	45.7	262 (194, 333)	42	187 (141, 234)	23.4	106 (77, 135)	26.6 (62.7)	32 (23, 42)	25.9 (18.5)	20 (14, 27)	15.9 (7.6)	30 (21, 39)	15.6 (11.3)
Wisconsin	6193 (4560, 7765)	51.3	216 (159, 271)	41.8	166 (122, 208)	20.3	94 (68, 119)	23.1 (63.2)	28 (20, 38)	24.1 (19.8)	17 (12, 23)	11.8 (6.4)	26 (18, 35)	12.9 (10.6)
Wyoming	569 (419, 721)	61.7	191 (141, 242)	36.5	153 (114, 192)	19.5	86 (64, 111)	21.5 (61.5)	26 (19, 35)	22.7 (19.4)	16 (11, 22)	12.4 (7.1)	24 (17, 33)	14.2 (12)

YLD: Years Lost due to Disability
UI is the uncertainty interval, it reflects the certainty of an estimate based on data availability, studies size and consistency across data sources
*Percentage attributed to overall rate is the proportion of overall age-standardized rate change attributed by each of the following four causes: diabetes, hypertension, glomerulonephritis and other causes

© Bowe B et al. *JAMA Network Open*

eTable5: Years of life lost (YLL) due to CKD in the United States and by state and district in the overall population, and in females and males.

eTable 5a: Number and age-standardized rate for overall YLL due to CKD and by cause, with percentage change from 2002 to 2016 for the Unites States and by state and district.

location	YLL number		YLL per 100,000 persons		Age-standardized YLL per 100,000 persons									
					All causes		Due to diabetes		Due to hypertension		Due to glomerulonephritis		Due to other causes	
	2016 (UI)	Percent change from 2002	2016 (UI)	Percent change from 2002	2016 (UI)	Percent change from 2002	2016 (UI)	Percent change from 2002 (PA*)	2016 (UI)	Percent change from 2002 (PA*)	2016 (UI)	Percent change from 2002 (PA*)	2016 (UI)	Percent change from 2002 (PA*)
United States	1212506 (1179966, 1243846)	55.6	376 (365, 385)	38.6	270 (263, 277)	19.3	151 (140, 162)	22.6 (64)	53 (46, 62)	22.5 (22.4)	25 (21, 29)	10.2 (5.4)	40 (34, 47)	9.8 (8.2)
Alabama	28079 (24728, 31384)	47.9	578 (509, 646)	36.4	414 (364, 463)	20.1	233 (200, 265)	24.5 (66.5)	76 (63, 92)	17.6 (16.4)	41 (33, 50)	13.9 (7.2)	64 (52, 77)	12 (9.9)
Alaska	1545 (1356, 1742)	85.9	210 (184, 237)	63.4	201 (176, 226)	25.8	109 (94, 125)	29 (59.7)	40 (33, 48)	32.8 (24)	20 (16, 24)	14.9 (6.3)	31 (25, 38)	15.2 (10)
Arizona	21928 (19820, 24041)	77.8	317 (287, 348)	38.9	224 (203, 245)	16.7	126 (111, 141)	21.2 (68.9)	42 (36, 50)	16.7 (18.9)	21 (17, 26)	8.2 (5.1)	34 (28, 40)	7.3 (7.1)
Arkansas	15565 (14194, 17021)	60.7	523 (477, 572)	46.1	373 (341, 409)	32.3	210 (186, 235)	37.4 (62.8)	70 (59, 81)	35.2 (19.8)	37 (30, 44)	20.5 (6.8)	57 (48, 68)	20.4 (10.6)

© Bowe B et al. *JAMA Network Open*

location	YLL number		YLL per 100,000 persons		Age-standardized YLL per 100,000 persons									
					All causes		Due to diabetes		Due to hypertension		Due to glomerulonephritis		Due to other causes	
	2016 (UI)	Percent change from 2002	2016 (UI)	Percent change from 2002	2016 (UI)	Percent change from 2002	2016 (UI)	Percent change from 2002 (PA*)	2016 (UI)	Percent change from 2002 (PA*)	2016 (UI)	Percent change from 2002 (PA*)	2016 (UI)	Percent change from 2002 (PA*)
California	109134 (97343, 120602)	75.6	277 (247, 306)	55.6	213 (190, 235)	27.8	120 (105, 137)	31.3 (62)	43 (35, 51)	36 (24.3)	19 (15, 23)	13.5 (4.9)	31 (25, 38)	15.2 (8.8)
Colorado	12694 (11574, 13889)	69.9	230 (210, 252)	38.5	180 (164, 197)	11.4	99 (87, 111)	14.6 (68.4)	38 (32, 44)	12.7 (23)	17 (14, 20)	4.5 (3.9)	27 (22, 32)	3.3 (4.7)
Connecticut	10635 (9532, 11911)	26.1	297 (266, 333)	21.5	196 (175, 220)	6.5	108 (94, 124)	7.6 (63.1)	41 (34, 48)	11.5 (34.8)	18 (15, 22)	0.9 (1.4)	29 (24, 35)	-0.3 (0.7)
Delaware	3938 (3618, 4270)	68.3	413 (380, 448)	42.4	278 (255, 301)	18	156 (137, 173)	21.6 (65.2)	53 (45, 62)	19.3 (20.1)	27 (22, 32)	11.6 (6.7)	42 (35, 50)	8.7 (8)
District of Columbia	2696 (2395, 3026)	0.3	394 (350, 442)	-15.1	353 (313, 397)	-12.9	205 (177, 234)	-9.4 (40.6)	63 (52, 76)	-4.7 (5.9)	33 (26, 40)	-26.9 (23)	52 (42, 64)	-23.3 (30.4)
Florida	77859 (70554, 85859)	70.6	381 (345, 420)	39.1	235 (213, 260)	24.6	133 (117, 150)	29.9 (66.1)	44 (37, 52)	26.4 (19.7)	22 (18, 27)	12.4 (5.3)	35 (30, 43)	13.1 (8.9)
Georgia	48315 (43241, 53927)	65.2	470 (421, 525)	36.9	377 (337, 420)	11.3	212 (184, 240)	13.8 (67.3)	73 (61, 87)	15 (25.1)	35 (28, 43)	3.8 (3.4)	56 (46, 67)	3 (4.3)

© Bowe B et al. *JAMA Network Open*

location	YLL number		YLL per 100,000 persons		Age-standardized YLL per 100,000 persons									
					All causes		Due to diabetes		Due to hypertension		Due to glomerulonephritis		Due to other causes	
	2016 (UI)	Percent change from 2002	2016 (UI)	Percent change from 2002	2016 (UI)	Percent change from 2002	2016 (UI)	Percent change from 2002 (PA*)	2016 (UI)	Percent change from 2002 (PA*)	2016 (UI)	Percent change from 2002 (PA*)	2016 (UI)	Percent change from 2002 (PA*)
Hawaii	4579 (4207, 4989)	70.8	318 (292, 346)	47.8	216 (198, 234)	25.9	122 (108, 136)	30.4 (64.3)	41 (35, 49)	27.5 (20)	20 (16, 24)	15.9 (6.2)	32 (27, 38)	15.2 (9.5)
Idaho	4630 (4117, 5180)	100.2	276 (246, 309)	59.8	205 (182, 230)	34.7	112 (97, 129)	38.4 (58.8)	43 (35, 51)	39.1 (22.8)	19 (15, 23)	24.5 (7.1)	31 (25, 37)	23.8 (11.2)
Illinois	51079 (46812, 55429)	31.3	398 (365, 432)	28.2	288 (264, 313)	8.7	161 (143, 181)	10.9 (66.2)	59 (49, 69)	15.1 (32)	26 (21, 31)	-1 (1.1)	43 (35, 51)	-0.4 (0.8)
Indiana	30636 (27386, 34394)	56.3	462 (413, 519)	45.1	335 (299, 377)	26.6	185 (160, 214)	29.8 (60.4)	68 (56, 82)	28.5 (21.3)	32 (26, 38)	19.9 (7.5)	51 (42, 62)	17.7 (10.8)
Iowa	8961 (8150, 9839)	62.4	286 (260, 314)	51.8	189 (171, 207)	40.1	104 (91, 117)	44.1 (58.8)	40 (33, 47)	46 (23.1)	17 (14, 21)	27.2 (6.8)	28 (23, 34)	27.8 (11.3)
Kansas	10999 (9764, 12345)	46.2	378 (336, 425)	36.4	272 (240, 307)	24.7	151 (129, 173)	29.1 (63.3)	55 (46, 66)	24.4 (20)	25 (21, 31)	16.8 (6.8)	41 (33, 50)	15.1 (10)
Kentucky	22743 (20952, 24704)	63.3	514 (473, 558)	50.9	367 (339, 398)	30.3	205 (183, 231)	34.8 (62)	72 (60, 84)	30.1 (19.5)	35 (29, 41)	22.3 (7.4)	55 (46, 65)	20.8 (11.1)

© Bowe B et al. *JAMA Network Open*

location	YLL number		YLL per 100,000 persons		Age-standardized YLL per 100,000 persons									
					All causes		Due to diabetes		Due to hypertension		Due to glomerulonephritis		Due to other causes	
	2016 (UI)	Percent change from 2002	2016 (UI)	Percent change from 2002	2016 (UI)	Percent change from 2002	2016 (UI)	Percent change from 2002 (PA*)	2016 (UI)	Percent change from 2002 (PA*)	2016 (UI)	Percent change from 2002 (PA*)	2016 (UI)	Percent change from 2002 (PA*)
Louisiana	29904 (27750, 32623)	40.5	639 (593, 697)	35.1	489 (453, 532)	16.3	275 (248, 307)	19.5 (65.6)	91 (77, 107)	20.3 (22.4)	48 (39, 57)	7.3 (4.8)	75 (63, 88)	7.1 (7.2)
Maine	5164 (4743, 5605)	47.3	389 (357, 422)	43.4	229 (211, 249)	17.3	125 (111, 139)	19.6 (60.3)	48 (40, 57)	18.2 (21.9)	22 (18, 26)	12.9 (7.4)	35 (29, 42)	11.3 (10.5)
Maryland	21231 (19553, 23038)	38.5	352 (324, 382)	24.8	259 (239, 281)	5.7	145 (129, 162)	7.9 (62.1)	50 (42, 59)	10.9 (28.9)	25 (20, 29)	-2.4 (3.5)	39 (32, 47)	-2.3 (5.5)
Massachusetts	22393 (20464, 24280)	24.8	328 (300, 356)	17.1	222 (202, 241)	4	122 (108, 137)	5.1 (59.4)	47 (40, 55)	7.7 (33.4)	20 (16, 24)	-0.3 (0.6)	33 (27, 39)	-2 (6.6)
Michigan	40162 (36943, 43278)	42	405 (373, 437)	43.3	276 (253, 297)	17.8	153 (138, 169)	20.6 (62.8)	55 (47, 65)	20 (22)	26 (22, 31)	11.4 (6.4)	41 (35, 50)	9.6 (8.8)
Minnesota	16207 (14841, 17829)	56.6	294 (269, 324)	42.4	203 (186, 224)	21.7	110 (95, 123)	25.1 (60.7)	45 (38, 54)	24 (24)	18 (15, 22)	13.1 (5.9)	30 (25, 37)	12.6 (9.4)
Mississippi	19897 (17639, 22308)	52.2	666 (590, 746)	45.5	499 (443, 562)	25.3	283 (244, 324)	29.9 (64.5)	91 (75, 110)	27.2 (19.2)	49 (40, 59)	15.2 (6.5)	77 (63, 91)	14.9 (9.8)

© Bowe B et al. *JAMA Network Open*

location	YLL number		YLL per 100,000 persons		Age-standardized YLL per 100,000 persons									
					All causes		Due to diabetes		Due to hypertension		Due to glomerulonephritis		Due to other causes	
	2016 (UI)	Percent change from 2002	2016 (UI)	Percent change from 2002	2016 (UI)	Percent change from 2002	2016 (UI)	Percent change from 2002 (PA*)	2016 (UI)	Percent change from 2002 (PA*)	2016 (UI)	Percent change from 2002 (PA*)	2016 (UI)	Percent change from 2002 (PA*)
Missouri	26708 (24729, 28924)	53.1	439 (406, 475)	42.6	304 (281, 329)	25.5	170 (151, 189)	30 (63.6)	60 (51, 71)	26.6 (20.3)	29 (24, 34)	15.9 (6.4)	46 (38, 55)	15.2 (9.8)
Montana	2847 (2522, 3187)	53.9	275 (243, 307)	35.2	182 (161, 203)	17.1	99 (86, 115)	21.3 (66)	36 (30, 43)	17 (19.9)	18 (15, 22)	10 (6.2)	28 (23, 34)	8.1 (7.9)
Nebraska	6310 (5780, 6847)	47	331 (303, 359)	33.3	237 (216, 258)	22.5	130 (115, 145)	25.8 (61.4)	49 (41, 58)	26.6 (23.7)	22 (18, 26)	13.3 (5.9)	35 (29, 43)	12.4 (9)
Nevada	11471 (10418, 12532)	65.2	391 (355, 427)	22.2	292 (264, 318)	1.3	164 (144, 184)	4.2 (62.2)	56 (48, 66)	0.9 (4.8)	27 (22, 33)	-3.6 (9.5)	44 (36, 53)	-5.4 (23.4)
New Hampshire	3850 (3534, 4205)	63.7	289 (265, 315)	55	186 (171, 204)	21.4	101 (89, 113)	22.9 (57.2)	39 (33, 47)	22.2 (21.7)	18 (15, 21)	20.4 (9.2)	28 (24, 34)	16.1 (11.9)
New Jersey	30964 (28134, 34145)	21.3	346 (314, 381)	15.6	239 (217, 264)	1.4	133 (117, 150)	2.9 (39.5)	49 (41, 58)	5.7 (28)	22 (18, 26)	-4.5 (11.1)	36 (29, 43)	-5.4 (21.4)
New Mexico	7918 (6996, 8932)	86.8	382 (338, 431)	67.3	273 (241, 309)	37.8	153 (132, 176)	43 (61.4)	51 (42, 60)	38.3 (18.8)	27 (22, 33)	28.5 (8)	42 (34, 51)	26.5 (11.7)

© Bowe B et al. *JAMA Network Open*

location	YLL number		YLL per 100,000 persons		Age-standardized YLL per 100,000 persons									
					All causes		Due to diabetes		Due to hypertension		Due to glomerulonephritis		Due to other causes	
	2016 (UI)	Percent change from 2002	2016 (UI)	Percent change from 2002	2016 (UI)	Percent change from 2002	2016 (UI)	Percent change from 2002 (PA*)	2016 (UI)	Percent change from 2002 (PA*)	2016 (UI)	Percent change from 2002 (PA*)	2016 (UI)	Percent change from 2002 (PA*)
New York	56578 (50126, 63841)	24.6	286 (253, 322)	20.5	201 (178, 228)	4.7	113 (97, 130)	6.1 (55.1)	40 (33, 49)	10.8 (33.2)	18 (15, 22)	-2.7 (4.3)	30 (24, 36)	-2.8 (7.4)
North Carolina	46975 (43242, 50588)	69.1	465 (428, 501)	39.4	334 (308, 360)	17.6	189 (168, 209)	21 (65.2)	65 (55, 76)	19.4 (20.9)	31 (25, 37)	10.1 (5.7)	50 (41, 59)	8.9 (8.1)
North Dakota	2176 (1958, 2403)	49.8	282 (254, 312)	24.1	211 (189, 234)	27.6	116 (102, 131)	31 (60.2)	42 (35, 50)	29 (20.8)	20 (17, 25)	20.9 (7.8)	32 (27, 39)	18.8 (11.2)
Ohio	51811 (48162, 55915)	44	446 (415, 482)	41.5	305 (283, 330)	22.3	171 (152, 189)	25.8 (62.8)	60 (50, 71)	22.8 (20)	29 (23, 34)	16.1 (7.2)	46 (38, 54)	14 (10.1)
Oklahoma	17021 (15729, 18512)	70.4	435 (402, 473)	51.9	325 (300, 354)	40	183 (164, 202)	46 (61.9)	62 (52, 73)	39.7 (18.8)	31 (26, 38)	29.1 (7.6)	50 (42, 59)	27.9 (11.6)
Oregon	11423 (10511, 12436)	69.3	283 (260, 308)	46.9	187 (172, 203)	23.4	103 (91, 115)	26.7 (61.3)	39 (33, 46)	27.3 (23.8)	17 (14, 20)	13.9 (5.8)	27 (23, 33)	13.3 (9.1)
Pennsylvania	55315 (51280, 59991)	26.2	433 (402, 470)	21.7	276 (256, 299)	10.2	154 (137, 171)	12.6 (67.6)	56 (47, 66)	13.4 (26)	25 (21, 30)	3 (2.9)	41 (34, 49)	2.2 (3.5)

© Bowe B et al. *JAMA Network Open*

location	YLL number		YLL per 100,000 persons		Age-standardized YLL per 100,000 persons									
					All causes		Due to diabetes		Due to hypertension		Due to glomerulonephritis		Due to other causes	
	2016 (UI)	Percent change from 2002	2016 (UI)	Percent change from 2002	2016 (UI)	Percent change from 2002	2016 (UI)	Percent change from 2002 (PA*)	2016 (UI)	Percent change from 2002 (PA*)	2016 (UI)	Percent change from 2002 (PA*)	2016 (UI)	Percent change from 2002 (PA*)
Rhode Island	3143 (2790, 3508)	30.7	298 (264, 332)	31.6	196 (174, 220)	17.1	109 (94, 125)	19 (60.6)	40 (33, 48)	22.7 (25.9)	18 (14, 22)	9.2 (5.4)	29 (24, 35)	8.6 (8.1)
South Carolina	26010 (23399, 29053)	65.9	527 (474, 589)	38	367 (331, 410)	13.6	207 (182, 236)	16.2 (65.5)	70 (59, 84)	17.9 (24.1)	35 (28, 42)	5.9 (4.4)	55 (45, 66)	5 (6)
South Dakota	2523 (2287, 2804)	54.2	292 (264, 324)	35.6	205 (185, 227)	25.9	112 (99, 127)	30.6 (62.5)	40 (33, 47)	26.1 (19.5)	21 (17, 25)	17.8 (7.4)	32 (27, 39)	16.1 (10.6)
Tennessee	30007 (27455, 32264)	77.1	453 (414, 487)	54.9	325 (297, 349)	33.3	183 (161, 202)	38.1 (62.2)	62 (52, 73)	37.2 (20.8)	31 (25, 37)	20.5 (6.5)	49 (41, 58)	21 (10.5)
Texas	105665 (96757, 114918)	102.9	379 (347, 413)	58.5	323 (296, 351)	36.2	183 (163, 203)	41.1 (62.1)	63 (54, 75)	39.8 (20.8)	30 (24, 35)	23.2 (6.5)	48 (39, 57)	23.7 (10.6)
Utah	6898 (6363, 7478)	91.9	228 (210, 247)	48.3	221 (203, 239)	27.9	121 (107, 135)	31.2 (59.7)	47 (39, 55)	31.4 (23.3)	20 (17, 24)	18.9 (6.6)	33 (27, 39)	17.9 (10.3)
Vermont	1672 (1528, 1845)	46.4	268 (245, 295)	43.8	168 (154, 185)	16	91 (80, 103)	17.5 (58.4)	35 (30, 42)	18.3 (23.5)	16 (13, 19)	13 (8)	26 (21, 30)	10.1 (10.1)

© Bowe B et al. *JAMA Network Open*

location	YLL number		YLL per 100,000 persons		Age-standardized YLL per 100,000 persons									
					All causes		Due to diabetes		Due to hypertension		Due to glomerulonephritis		Due to other causes	
	2016 (UI)	Percent change from 2002	2016 (UI)	Percent change from 2002	2016 (UI)	Percent change from 2002	2016 (UI)	Percent change from 2002 (PA*)	2016 (UI)	Percent change from 2002 (PA*)	2016 (UI)	Percent change from 2002 (PA*)	2016 (UI)	Percent change from 2002 (PA*)
Virginia	33663 (30988, 36495)	54.3	400 (368, 433)	33.4	294 (271, 319)	11.9	164 (146, 183)	14.6 (66.7)	60 (50, 71)	16 (26.3)	27 (22, 32)	3.4 (2.8)	44 (36, 52)	3.1 (4.2)
Washington	17273 (15926, 18664)	95.3	239 (221, 259)	63.5	172 (158, 186)	36.3	95 (84, 106)	40.1 (59.2)	37 (31, 43)	42.6 (23.7)	15 (13, 18)	23.5 (6.4)	25 (21, 30)	23.8 (10.6)
West Virginia	11405 (10513, 12400)	55.1	623 (574, 677)	52.8	397 (366, 432)	36.4	222 (199, 248)	41.2 (61.2)	76 (64, 91)	36.4 (19.2)	38 (31, 46)	27.3 (7.8)	60 (50, 72)	26.3 (11.9)
Wisconsin	20410 (18823, 22012)	55.6	354 (326, 381)	46.5	236 (217, 254)	24.9	128 (114, 143)	27.7 (59.3)	50 (43, 59)	29.3 (24.3)	22 (18, 26)	16.3 (6.5)	35 (29, 42)	15.3 (9.9)
Wyoming	1496 (1341, 1671)	57.1	256 (229, 286)	34.2	192 (172, 215)	18.3	106 (92, 121)	22.8 (66.2)	38 (31, 45)	19.7 (20.9)	19 (15, 22)	9.1 (5.3)	29 (24, 35)	8.2 (7.6)

YLL: Years of Life Lost
UI is the uncertainty interval, it reflects the certainty of an estimate based on data availability, studies size and consistency across data sources
*Percentage attributed to overall rate is the proportion of overall age-standardized rate change attributed by each of the following four causes: diabetes, hypertension, glomerulonephritis and other causes

© Bowe B et al. *JAMA Network Open*

eTable 5b: Number and age-standardized rate for overall YLL due to CKD and by cause in females, with percentage change from 2002 to 2016 for the Unites States and by state and district.

location	YLL number		YLL per 100,000 persons		Age-standardized YLL per 100,000 persons									
					All causes		Due to diabetes		Due to hypertension		Due to glomerulonephritis		Due to other causes	
	2016 (UI)	Percent change from 2002	2016 (UI)	Percent change from 2002	2016 (UI)	Percent change from 2002	2016 (UI)	Percent change from 2002 (PA*)	2016 (UI)	Percent change from 2002 (PA*)	2016 (UI)	Percent change from 2002 (PA*)	2016 (UI)	Percent change from 2002 (PA*)
United States	566633 (543974, 587975)	50.2	346 (332, 359)	34.1	231 (222, 239)	18.4	124 (113, 134)	21.1 (60.4)	47 (40, 55)	23.1 (24.6)	24 (20, 29)	9.9 (6.1)	36 (30, 42)	9.9 (9)
Alabama	13581 (11490, 15855)	43.1	541 (458, 632)	32.1	364 (308, 427)	20.8	197 (162, 236)	25.1 (63)	69 (55, 86)	19.6 (18)	41 (32, 51)	14.4 (8.2)	58 (46, 72)	13.3 (10.8)
Alaska	718 (595, 855)	83.1	207 (171, 246)	64.1	187 (155, 222)	25.1	98 (80, 118)	27.7 (56.7)	38 (30, 47)	32.1 (24.5)	21 (16, 27)	15.8 (7.7)	30 (24, 37)	16.1 (11.1)
Arizona	10046 (8706, 11461)	74.9	289 (250, 329)	36	192 (167, 218)	17	103 (88, 121)	20.2 (62.4)	38 (30, 46)	18.4 (20.9)	21 (16, 25)	10.7 (7.2)	30 (24, 36)	9.6 (9.4)
Arkansas	7554 (6572, 8548)	57.5	500 (435, 565)	44	334 (290, 379)	34.5	181 (153, 210)	39.4 (59.6)	64 (52, 77)	38.3 (20.6)	37 (29, 46)	23 (8.1)	53 (43, 65)	23.6 (11.7)
California	49664 (41760, 58188)	67.1	250 (210, 293)	47.6	178 (149, 209)	22.7	96 (80, 117)	24.8 (58.1)	37 (29, 46)	33.6 (28.2)	18 (14, 23)	10.1 (5)	27 (21, 34)	12 (8.7)

© Bowe B et al. *JAMA Network Open*

location	YLL number		YLL per 100,000 persons		Age-standardized YLL per 100,000 persons									
					All causes		Due to diabetes		Due to hypertension		Due to glomerulonephritis		Due to other causes	
	2016 (UI)	Percent change from 2002	2016 (UI)	Percent change from 2002	2016 (UI)	Percent change from 2002	2016 (UI)	Percent change from 2002 (PA*)	2016 (UI)	Percent change from 2002 (PA*)	2016 (UI)	Percent change from 2002 (PA*)	2016 (UI)	Percent change from 2002 (PA*)
Colorado	5782 (5141, 6580)	58.2	211 (188, 240)	28.7	152 (135, 174)	6.5	80 (68, 93)	8.2 (64.9)	33 (27, 40)	10.2 (32.6)	16 (13, 20)	1.3 (2.2)	24 (19, 29)	0.1 (0.3)
Connecticut	4774 (3995, 5630)	20.3	261 (218, 307)	16.4	157 (131, 185)	3.5	83 (67, 99)	3.8 (45.1)	33 (27, 41)	9.9 (45.2)	16 (13, 21)	-1.1 (2.8)	24 (19, 30)	-1.9 (6.9)
Delaware	1835 (1617, 2069)	68.4	374 (329, 421)	42.1	235 (206, 264)	19.9	126 (108, 145)	22.7 (59.8)	46 (38, 55)	22 (21.4)	26 (21, 32)	14.5 (8.4)	37 (30, 45)	12.3 (10.5)
District of Columbia	1184 (992, 1403)	-1.6	330 (276, 391)	-16	280 (235, 333)	-11.7	156 (127, 187)	-9.2 (42.4)	52 (41, 65)	-4.2 (6.1)	30 (23, 37)	-22.3 (22.8)	43 (33, 55)	-19.8 (28.7)
Florida	33800 (29400, 38765)	63.4	323 (281, 371)	33.4	188 (164, 217)	21.3	102 (87, 120)	26.1 (63.9)	36 (29, 43)	24.6 (21.4)	21 (16, 26)	10 (5.6)	30 (24, 36)	11.2 (9)
Georgia	23321 (19621, 27487)	55.9	442 (372, 521)	28.1	330 (278, 390)	7.5	178 (147, 212)	9.3 (65.9)	67 (53, 83)	12.9 (33.1)	35 (27, 43)	0.3 (0.5)	51 (41, 64)	0.2 (0.4)
Hawaii	2023 (1773, 2285)	69.2	285 (250, 322)	47.7	176 (154, 199)	23.7	96 (82, 111)	27.7 (61.4)	35 (28, 42)	27.4 (22.1)	19 (15, 23)	13.7 (6.7)	27 (22, 33)	13.8 (9.8)

© Bowe B et al. *JAMA Network Open*

location	YLL number		YLL per 100,000 persons		Age-standardized YLL per 100,000 persons									
					All causes		Due to diabetes		Due to hypertension		Due to glomerulonephritis		Due to other causes	
	2016 (UI)	Percent change from 2002	2016 (UI)	Percent change from 2002	2016 (UI)	Percent change from 2002	2016 (UI)	Percent change from 2002 (PA*)	2016 (UI)	Percent change from 2002 (PA*)	2016 (UI)	Percent change from 2002 (PA*)	2016 (UI)	Percent change from 2002 (PA*)
Idaho	2154 (1844, 2511)	96.1	258 (221, 300)	56.6	179 (153, 209)	35.2	94 (78, 113)	37.7 (55.2)	38 (30, 47)	41.7 (24.2)	19 (15, 23)	26 (8.3)	28 (22, 35)	25.8 (12.2)
Illinois	23564 (20935, 26618)	25.2	361 (320, 407)	22.5	240 (213, 272)	6.7	128 (110, 149)	8.1 (57.4)	50 (41, 61)	14.4 (37.8)	25 (20, 30)	-1.6 (2.4)	37 (30, 45)	-1.1 (2.4)
Indiana	14760 (12486, 17444)	53.1	439 (371, 519)	42.5	293 (248, 347)	28.7	156 (128, 192)	31.7 (57.4)	61 (49, 76)	32.1 (22.6)	31 (24, 39)	21.4 (8.4)	46 (36, 57)	20 (11.6)
Iowa	4212 (3671, 4778)	54.4	267 (233, 303)	45.9	160 (139, 182)	38.6	85 (71, 99)	42.2 (56.3)	34 (28, 42)	46.5 (24.5)	17 (13, 20)	25.4 (7.5)	25 (20, 30)	26.9 (11.7)
Kansas	5214 (4437, 6181)	38.8	358 (304, 424)	30.5	237 (199, 282)	23.4	127 (104, 154)	27 (60)	49 (38, 61)	24.2 (21.1)	25 (19, 31)	17 (8)	37 (29, 47)	15.4 (10.9)
Kentucky	10839 (9601, 12134)	57.5	482 (427, 540)	46.3	320 (283, 358)	30.9	172 (148, 199)	34.9 (58.8)	64 (52, 78)	32.2 (20.7)	34 (28, 42)	23.1 (8.5)	50 (41, 60)	22.3 (12)
Louisiana	14586 (12841, 16390)	37	610 (537, 685)	32.7	437 (385, 492)	16.8	237 (203, 273)	19.4 (61.1)	83 (68, 101)	21.4 (23.4)	48 (38, 58)	9.4 (6.5)	69 (55, 83)	9 (9)

© Bowe B et al. *JAMA Network Open*

location	YLL number		YLL per 100,000 persons		Age-standardized YLL per 100,000 persons									
					All causes		Due to diabetes		Due to hypertension		Due to glomerulonephritis		Due to other causes	
	2016 (UI)	Percent change from 2002	2016 (UI)	Percent change from 2002	2016 (UI)	Percent change from 2002	2016 (UI)	Percent change from 2002 (PA*)	2016 (UI)	Percent change from 2002 (PA*)	2016 (UI)	Percent change from 2002 (PA*)	2016 (UI)	Percent change from 2002 (PA*)
Maine	2398 (2127, 2668)	38.5	354 (314, 394)	35.5	192 (171, 214)	13.6	100 (86, 115)	15.5 (58.3)	42 (34, 50)	17.1 (26.3)	20 (16, 25)	7.9 (6.5)	30 (24, 36)	7.3 (8.9)
Maryland	9872 (8776, 11104)	37.3	318 (282, 357)	24.2	217 (193, 244)	5.8	116 (100, 134)	7.5 (61.4)	44 (35, 53)	11.4 (33.7)	23 (18, 28)	-1.1 (2)	34 (27, 41)	-1.1 (2.8)
Massachusetts	9884 (8650, 11212)	17.6	281 (246, 319)	10.9	173 (151, 197)	1.7	91 (77, 107)	2.5 (40.5)	38 (31, 46)	5.5 (36.5)	18 (14, 22)	-1.8 (6.2)	27 (21, 33)	-3.3 (16.8)
Michigan	19003 (16903, 21162)	36.5	377 (335, 420)	38	237 (211, 264)	16	127 (109, 144)	18 (59)	49 (40, 59)	19.6 (24.4)	25 (20, 30)	10.4 (7.2)	37 (30, 45)	9 (9.3)
Minnesota	7487 (6610, 8466)	51.6	270 (239, 306)	38.3	171 (150, 193)	23.2	88 (75, 103)	26.3 (57.2)	39 (31, 47)	26.8 (25.4)	18 (14, 22)	14.5 (6.9)	26 (21, 33)	14.7 (10.5)
Mississippi	9739 (8082, 11324)	46.7	632 (525, 735)	40.5	446 (369, 522)	24.8	242 (197, 290)	29.2 (61.8)	83 (65, 102)	28.7 (20.9)	50 (39, 62)	14.2 (7)	71 (55, 88)	14.7 (10.3)
Missouri	12507 (11117, 13905)	47.8	404 (359, 449)	38.7	258 (228, 288)	25.8	138 (120, 158)	30.3 (60.6)	52 (43, 63)	27.7 (21.4)	27 (22, 33)	16.6 (7.4)	40 (32, 48)	16.3 (10.6)

© Bowe B et al. *JAMA Network Open*

location	YLL number		YLL per 100,000 persons		Age-standardized YLL per 100,000 persons									
					All causes		Due to diabetes		Due to hypertension		Due to glomerulonephritis		Due to other causes	
	2016 (UI)	Percent change from 2002	2016 (UI)	Percent change from 2002	2016 (UI)	Percent change from 2002	2016 (UI)	Percent change from 2002 (PA*)	2016 (UI)	Percent change from 2002 (PA*)	2016 (UI)	Percent change from 2002 (PA*)	2016 (UI)	Percent change from 2002 (PA*)
Montana	1338 (1142, 1560)	51.9	260 (222, 303)	34.4	162 (138, 190)	19.6	85 (70, 103)	22.8 (59.5)	33 (26, 41)	21.9 (22.5)	18 (14, 22)	13.3 (8)	26 (20, 32)	11.6 (10.1)
Nebraska	3026 (2687, 3390)	44.6	317 (282, 355)	32.6	208 (184, 234)	24.7	110 (94, 127)	27.9 (58.3)	44 (36, 53)	31.1 (25.3)	22 (17, 27)	13.8 (6.4)	32 (26, 39)	14.7 (10)
Nevada	4762 (4135, 5388)	63.7	326 (283, 368)	19.4	232 (201, 262)	0.7	125 (105, 145)	3.3 (51.2)	47 (38, 56)	1.4 (8.5)	25 (20, 30)	-4.3 (14.2)	36 (29, 44)	-5.4 (26.1)
New Hampshire	1748 (1555, 1979)	51.2	259 (231, 294)	43.6	155 (137, 176)	16	80 (69, 93)	16.3 (52.7)	34 (27, 40)	17.9 (23.8)	17 (14, 21)	16.7 (11.2)	24 (20, 30)	12.1 (12.3)
New Jersey	13776 (11983, 15639)	15.3	301 (262, 341)	10.4	189 (164, 216)	-1.3	101 (85, 119)	0 (0.8)	40 (32, 49)	3.7 (26)	20 (16, 24)	-7.6 (29.6)	29 (23, 36)	-7.6 (43.6)
New Mexico	3645 (3048, 4350)	90.4	349 (292, 416)	71.8	233 (194, 278)	42.1	125 (102, 153)	46.8 (57.8)	45 (36, 55)	42.8 (19.6)	26 (20, 33)	34.2 (9.6)	37 (29, 46)	32.2 (13)
New York	25781 (21532, 30200)	20	253 (211, 296)	16.8	164 (135, 194)	3.3	88 (72, 108)	4.3 (44.4)	34 (26, 42)	10.2 (38.1)	17 (13, 21)	-3.3 (7.2)	25 (20, 32)	-3.2 (10.2)

© Bowe B et al. *JAMA Network Open*

location	YLL number		YLL per 100,000 persons		Age-standardized YLL per 100,000 persons									
					All causes		Due to diabetes		Due to hypertension		Due to glomerulonephritis		Due to other causes	
	2016 (UI)	Percent change from 2002	2016 (UI)	Percent change from 2002	2016 (UI)	Percent change from 2002	2016 (UI)	Percent change from 2002 (PA*)	2016 (UI)	Percent change from 2002 (PA*)	2016 (UI)	Percent change from 2002 (PA*)	2016 (UI)	Percent change from 2002 (PA*)
North Carolina	22836 (20204, 25387)	64.8	440 (390, 489)	35.1	295 (261, 327)	18.6	160 (138, 183)	22.1 (62.8)	58 (48, 70)	20.4 (21.4)	31 (25, 38)	10.8 (6.6)	45 (37, 55)	10.4 (9.3)
North Dakota	976 (841, 1131)	41.9	260 (224, 302)	20.8	176 (149, 206)	26.1	93 (78, 111)	30.1 (59)	36 (28, 44)	28.7 (22)	19 (15, 24)	16.8 (7.6)	28 (22, 34)	17.4 (11.3)
Ohio	24822 (22458, 27481)	37.8	419 (379, 464)	36.4	264 (239, 294)	20.7	142 (124, 161)	23 (58.4)	54 (44, 65)	24.4 (23.3)	28 (23, 34)	14.7 (7.9)	41 (33, 50)	13.1 (10.4)
Oklahoma	8229 (7319, 9256)	67.1	417 (371, 469)	49.7	290 (258, 327)	42.5	157 (135, 181)	48.1 (58.8)	57 (46, 68)	44.2 (20)	32 (25, 39)	31.3 (8.7)	46 (37, 55)	31 (12.5)
Oregon	5313 (4740, 5907)	61.2	260 (232, 290)	39.7	160 (142, 179)	20.3	84 (73, 97)	22.4 (57.5)	34 (28, 41)	25.4 (26)	16 (13, 20)	12.6 (6.8)	24 (20, 29)	12 (9.7)
Pennsylvania	25851 (23097, 28792)	22.6	397 (354, 442)	19.4	231 (204, 258)	10.6	123 (107, 140)	12.3 (60.9)	48 (40, 58)	15.3 (29)	24 (19, 29)	4.2 (4.5)	36 (29, 44)	3.6 (5.7)
Rhode Island	1455 (1240, 1694)	30.9	268 (228, 311)	32.7	160 (136, 187)	19.6	85 (71, 103)	20.9 (56.1)	34 (27, 42)	28.6 (28.8)	16 (13, 20)	10 (5.7)	24 (19, 30)	11.2 (9.4)

© Bowe B et al. *JAMA Network Open*

location	YLL number		YLL per 100,000 persons		Age-standardized YLL per 100,000 persons									
					All causes		Due to diabetes		Due to hypertension		Due to glomerulonephritis		Due to other causes	
	2016 (UI)	Percent change from 2002	2016 (UI)	Percent change from 2002	2016 (UI)	Percent change from 2002	2016 (UI)	Percent change from 2002 (PA*)	2016 (UI)	Percent change from 2002 (PA*)	2016 (UI)	Percent change from 2002 (PA*)	2016 (UI)	Percent change from 2002 (PA*)
South Carolina	12760 (10748, 15107)	65.7	503 (424, 596)	37.8	328 (276, 387)	16.8	177 (146, 216)	19.4 (61)	65 (51, 81)	21.9 (24.7)	35 (27, 44)	8.8 (6)	51 (39, 64)	8.4 (8.3)
South Dakota	1212 (1043, 1418)	55.6	282 (243, 330)	38.6	185 (158, 217)	31.1	98 (82, 117)	35.8 (58.9)	36 (29, 45)	34.1 (20.9)	21 (17, 26)	21.1 (8.4)	30 (24, 37)	20.9 (11.8)
Tennessee	14520 (12854, 16212)	72.2	428 (379, 478)	50.7	286 (252, 319)	33.8	154 (132, 176)	38.4 (59.1)	57 (46, 69)	40 (22.4)	31 (25, 37)	20.7 (7.3)	44 (36, 54)	22.2 (11.2)
Texas	50172 (44323, 56834)	95.3	358 (316, 406)	52.5	285 (252, 323)	34.4	155 (132, 179)	38.5 (59.1)	57 (47, 70)	40.9 (22.8)	29 (23, 36)	21.3 (7.1)	43 (34, 54)	22.9 (11.1)
Utah	3222 (2871, 3605)	90.3	214 (191, 239)	47.3	195 (173, 218)	29.9	103 (88, 119)	32.3 (55.8)	43 (35, 52)	35.5 (24.9)	20 (16, 25)	21.5 (7.8)	30 (24, 36)	21.1 (11.5)
Vermont	801 (698, 904)	36.6	253 (220, 285)	34.5	147 (128, 166)	11.2	76 (65, 88)	11.4 (53)	31 (25, 38)	14.7 (27.2)	16 (13, 20)	10 (10)	23 (19, 28)	6.7 (9.9)
Virginia	16059 (14250, 18025)	51.9	376 (333, 422)	31.9	256 (227, 287)	13	137 (117, 157)	15.4 (62.4)	53 (44, 65)	18.2 (27.9)	26 (21, 32)	4.2 (3.6)	39 (32, 48)	4.8 (6.1)

© Bowe B et al. *JAMA Network Open*

location	YLL number		YLL per 100,000 persons		Age-standardized YLL per 100,000 persons									
					All causes		Due to diabetes		Due to hypertension		Due to glomerulonephritis		Due to other causes	
	2016 (UI)	Percent change from 2002	2016 (UI)	Percent change from 2002	2016 (UI)	Percent change from 2002	2016 (UI)	Percent change from 2002 (PA*)	2016 (UI)	Percent change from 2002 (PA*)	2016 (UI)	Percent change from 2002 (PA*)	2016 (UI)	Percent change from 2002 (PA*)
Washington	8118 (7194, 9102)	86.8	225 (200, 253)	57.2	150 (133, 169)	33.3	79 (68, 91)	36 (56.1)	32 (26, 39)	40.7 (25)	15 (12, 19)	22.9 (7.6)	23 (18, 28)	22.8 (11.3)
West Virginia	5343 (4746, 6029)	44.5	576 (512, 651)	43.8	346 (307, 389)	33.9	186 (160, 212)	38.2 (58.7)	68 (55, 83)	34.7 (20)	38 (30, 46)	26 (8.9)	54 (44, 66)	24.9 (12.4)
Wisconsin	9700 (8651, 10871)	55.2	334 (298, 375)	46.7	205 (182, 230)	29	107 (93, 123)	31.6 (55.8)	45 (37, 54)	35.2 (25.3)	21 (17, 26)	19.8 (7.6)	32 (25, 38)	19.6 (11.3)
Wyoming	663 (565, 782)	47.8	232 (198, 274)	27.9	164 (139, 194)	16.1	87 (72, 106)	18.9 (60.9)	33 (26, 41)	19 (23)	18 (14, 23)	10 (7.2)	26 (21, 32)	8.4 (8.9)

YLL: Years of Life Lost
UI is the uncertainty interval, it reflects the certainty of an estimate based on data availability, studies size and consistency across data sources
*Percentage attributed to overall rate is the proportion of overall age-standardized rate change attributed by each of the following four causes: diabetes, hypertension, glomerulonephritis and other causes

© Bowe B et al. *JAMA Network Open*

eTable 5c: Number and age-standardized rate for overall YLL due to CKD and by cause in males, with percentage change from 2002 to 2016 for the Unites States and by state and district.

location	YLL number		YLL per 100,000 persons		Age-standardized YLL per 100,000 persons									
					All causes		Due to diabetes		Due to hypertension		Due to glomerulonephritis		Due to other causes	
	2016 (UI)	Percent change from 2002	2016 (UI)	Percent change from 2002	2016 (UI)	Percent change from 2002	2016 (UI)	Percent change from 2002 (PA*)	2016 (UI)	Percent change from 2002 (PA*)	2016 (UI)	Percent change from 2002 (PA*)	2016 (UI)	Percent change from 2002 (PA*)
United States	645873 (621356, 669078)	60.6	406 (391, 421)	42.7	317 (305, 329)	17.8	183 (168, 197)	21.7 (68.1)	62 (53, 73)	17.9 (19.7)	26 (21, 31)	9.9 (4.9)	46 (38, 54)	8.2 (7.3)
Alabama	14497 (12331, 17089)	52.6	616 (524, 726)	40.6	475 (404, 558)	17.2	277 (231, 330)	21.9 (71.5)	86 (70, 106)	11.9 (13.1)	41 (32, 51)	12.7 (6.7)	71 (56, 89)	9.4 (8.7)
Alaska	827 (692, 976)	88.4	213 (179, 252)	62.7	216 (182, 253)	25.8	122 (101, 145)	29.2 (61.9)	43 (35, 53)	31.8 (23.5)	19 (15, 24)	14.5 (5.4)	33 (26, 41)	14.2 (9.1)
Arizona	11881 (10283, 13497)	80.3	346 (300, 393)	41.6	259 (224, 294)	14.9	151 (128, 177)	20.6 (76.9)	48 (39, 58)	11.5 (14.8)	22 (17, 27)	5.6 (3.5)	38 (30, 47)	4.5 (4.8)
Arkansas	8011 (6986, 9065)	63.8	548 (478, 620)	48.1	419 (367, 475)	28.3	244 (208, 280)	33.8 (66.8)	77 (63, 92)	27.8 (18.1)	36 (29, 45)	17.3 (5.8)	62 (50, 76)	16.1 (9.3)
California	59470 (50560, 68417)	83.5	303 (258, 349)	63	253 (215, 291)	30.3	148 (123, 173)	34.8 (64.8)	50 (40, 61)	34.4 (21.6)	20 (15, 25)	16.6 (4.9)	36 (28, 45)	17 (8.8)

© Bowe B et al. *JAMA Network Open*

location	YLL number		YLL per 100,000 persons		Age-standardized YLL per 100,000 persons									
					All causes		Due to diabetes		Due to hypertension		Due to glomerulonephritis		Due to other causes	
	2016 (UI)	Percent change from 2002	2016 (UI)	Percent change from 2002	2016 (UI)	Percent change from 2002	2016 (UI)	Percent change from 2002 (PA*)	2016 (UI)	Percent change from 2002 (PA*)	2016 (UI)	Percent change from 2002 (PA*)	2016 (UI)	Percent change from 2002 (PA*)
Colorado	6912 (6131, 7755)	81.1	250 (221, 280)	48	212 (189, 238)	13.1	120 (103, 138)	17.4 (72.3)	44 (37, 54)	10.8 (17.6)	17 (14, 21)	7.1 (4.7)	31 (25, 38)	4.6 (5.5)
Connecticut	5861 (4992, 6873)	31.3	336 (286, 394)	25.8	245 (209, 287)	6.6	139 (116, 168)	8.4 (69.4)	51 (41, 63)	8.9 (26.9)	20 (16, 25)	2 (2.6)	35 (28, 45)	-0.5 (1.1)
Delaware	2103 (1867, 2363)	68.2	455 (404, 512)	42.6	330 (293, 371)	14.4	191 (164, 218)	18.9 (72.8)	62 (50, 74)	12.7 (16.7)	29 (23, 35)	8.3 (5.3)	49 (40, 59)	4.5 (5.1)
District of Columbia	1512 (1298, 1766)	1.8	464 (398, 542)	-14.5	444 (381, 519)	-14.8	265 (222, 313)	-10.7 (41.2)	78 (63, 97)	-7.6 (8.3)	37 (28, 47)	-30.8 (21.1)	64 (50, 81)	-26.3 (29.4)
Florida	44059 (38492, 50451)	76.6	441 (385, 505)	43.9	289 (252, 332)	25.4	169 (142, 197)	31.2 (68.5)	53 (44, 64)	24.3 (17.8)	24 (19, 30)	14.1 (5.1)	42 (34, 52)	13.5 (8.6)
Georgia	24994 (21389, 29479)	74.9	499 (427, 589)	46.3	434 (373, 509)	13.3	253 (213, 303)	16.2 (69.4)	83 (67, 102)	14.1 (20.2)	36 (28, 45)	7.4 (4.9)	63 (50, 78)	4.7 (5.5)
Hawaii	2556 (2275, 2866)	72.1	350 (311, 392)	47.7	261 (233, 294)	27	152 (132, 174)	31.6 (65.7)	50 (41, 60)	27 (19.1)	21 (17, 26)	17.9 (5.9)	38 (30, 46)	16.1 (9.4)

© Bowe B et al. *JAMA Network Open*

location	YLL number		YLL per 100,000 persons		Age-standardized YLL per 100,000 persons									
					All causes		Due to diabetes		Due to hypertension		Due to glomerulonephritis		Due to other causes	
	2016 (UI)	Percent change from 2002	2016 (UI)	Percent change from 2002	2016 (UI)	Percent change from 2002	2016 (UI)	Percent change from 2002 (PA*)	2016 (UI)	Percent change from 2002 (PA*)	2016 (UI)	Percent change from 2002 (PA*)	2016 (UI)	Percent change from 2002 (PA*)
Idaho	2476 (2106, 2911)	103.8	295 (251, 346)	62.6	234 (200, 275)	32.1	132 (109, 158)	36.7 (62.2)	48 (39, 59)	33.1 (21.2)	19 (15, 25)	22.5 (6.3)	34 (27, 43)	20.7 (10.3)
Illinois	27515 (24348, 30660)	37	436 (386, 486)	33.5	348 (309, 387)	8.3	200 (174, 227)	11.1 (70.7)	70 (58, 84)	11.9 (26.3)	28 (22, 34)	-1 (1)	50 (39, 62)	-1.1 (2)
Indiana	15876 (13505, 18531)	59.4	486 (413, 567)	47.5	388 (330, 452)	22	220 (181, 263)	25.6 (64.3)	78 (62, 97)	20.6 (19.1)	33 (25, 41)	17.3 (6.9)	57 (45, 70)	13.6 (9.7)
Iowa	4749 (4164, 5399)	70.1	305 (267, 347)	57.4	223 (196, 254)	38	126 (108, 147)	42.3 (61)	47 (39, 57)	39.7 (21.8)	18 (14, 22)	27.8 (6.4)	32 (26, 40)	26.3 (10.9)
Kansas	5785 (4894, 6801)	53.7	399 (338, 469)	42.2	316 (266, 371)	23	180 (148, 214)	28.1 (66.9)	64 (52, 78)	20 (18.1)	26 (20, 33)	15.9 (6.1)	46 (36, 57)	13.1 (9)
Kentucky	11904 (10584, 13395)	69	546 (486, 615)	55.3	426 (380, 478)	26.8	245 (214, 283)	31.7 (65.6)	83 (69, 101)	23.3 (17.5)	35 (28, 43)	20.4 (6.7)	62 (50, 75)	17.5 (10.2)
Louisiana	15318 (13590, 17165)	44	669 (594, 750)	37.4	553 (494, 619)	14.4	322 (279, 367)	18.2 (71)	102 (85, 122)	16.4 (20.6)	48 (38, 58)	5 (3.3)	82 (67, 99)	4.5 (5.1)

© Bowe B et al. *JAMA Network Open*

location	YLL number		YLL per 100,000 persons		Age-standardized YLL per 100,000 persons									
					All causes		Due to diabetes		Due to hypertension		Due to glomerulonephritis		Due to other causes	
	2016 (UI)	Percent change from 2002	2016 (UI)	Percent change from 2002	2016 (UI)	Percent change from 2002	2016 (UI)	Percent change from 2002 (PA*)	2016 (UI)	Percent change from 2002 (PA*)	2016 (UI)	Percent change from 2002 (PA*)	2016 (UI)	Percent change from 2002 (PA*)
Maine	2766 (2466, 3081)	55.9	425 (379, 474)	51	274 (246, 305)	17.6	153 (132, 174)	20.1 (62.6)	57 (47, 69)	15.1 (18.3)	23 (19, 28)	16.8 (8.2)	40 (33, 49)	12.5 (11)
Maryland	11359 (10160, 12763)	39.6	389 (348, 437)	25.3	312 (280, 349)	4.2	180 (158, 206)	6.8 (61.6)	60 (49, 72)	7.3 (21.7)	27 (21, 33)	-3.9 (5.8)	46 (36, 56)	-4.3 (11)
Massachusetts	12509 (11072, 14074)	31.1	377 (334, 424)	22.3	285 (252, 320)	3.4	160 (137, 185)	4.8 (63.2)	61 (51, 73)	5.5 (27)	23 (18, 28)	0.1 (0.2)	41 (33, 50)	-2.7 (9.7)
Michigan	21159 (18888, 23645)	47.2	434 (388, 485)	48.3	323 (288, 360)	17	184 (161, 210)	20.5 (66.7)	64 (54, 77)	16.5 (19.3)	27 (22, 33)	11.7 (6)	47 (38, 57)	8.7 (8)
Minnesota	8720 (7697, 9906)	61.1	318 (281, 362)	46	243 (214, 276)	17.1	135 (115, 155)	21.1 (66.2)	54 (44, 66)	15.9 (20.9)	19 (15, 24)	10.4 (5.2)	35 (28, 43)	8.5 (7.7)
Mississippi	10158 (8511, 11776)	57.8	701 (587, 813)	50.6	566 (478, 654)	23.8	333 (275, 395)	28.6 (67.9)	101 (81, 125)	22.1 (16.8)	49 (38, 61)	15.8 (6.1)	83 (65, 105)	13.7 (9.2)
Missouri	14201 (12628, 15887)	58	476 (423, 532)	46.2	359 (320, 401)	22.4	207 (180, 235)	27.2 (67.3)	70 (59, 84)	20.6 (18.2)	30 (24, 37)	14.2 (5.7)	52 (43, 64)	12.3 (8.8)

© Bowe B et al. *JAMA Network Open*

location	YLL number		YLL per 100,000 persons		Age-standardized YLL per 100,000 persons									
					All causes		Due to diabetes		Due to hypertension		Due to glomerulonephritis		Due to other causes	
	2016 (UI)	Percent change from 2002	2016 (UI)	Percent change from 2002	2016 (UI)	Percent change from 2002	2016 (UI)	Percent change from 2002 (PA*)	2016 (UI)	Percent change from 2002 (PA*)	2016 (UI)	Percent change from 2002 (PA*)	2016 (UI)	Percent change from 2002 (PA*)
Montana	1510 (1268, 1783)	55.8	289 (243, 342)	35.7	204 (171, 241)	12.2	115 (94, 140)	17.6 (77.8)	40 (32, 50)	7.8 (13.1)	18 (14, 22)	6 (4.6)	31 (24, 38)	3.4 (4.5)
Nebraska	3284 (2916, 3697)	49.2	345 (306, 388)	33.9	272 (242, 306)	17.5	154 (132, 177)	21.3 (66.3)	57 (47, 68)	17.5 (20.8)	22 (18, 28)	11.3 (5.6)	39 (32, 49)	8.1 (7.3)
Nevada	6708 (5919, 7555)	66.3	457 (403, 514)	24.7	356 (314, 400)	0.4	206 (178, 236)	3.9 (55.6)	67 (56, 81)	-2.2 (10.9)	30 (24, 37)	-3.5 (7.9)	52 (42, 63)	-6.3 (25.5)
New Hampshire	2103 (1871, 2352)	75.7	319 (284, 357)	65.9	224 (200, 252)	23.1	125 (108, 144)	25.5 (60.1)	47 (39, 57)	21.3 (19.8)	19 (15, 23)	23.2 (8.5)	33 (27, 41)	17.4 (11.6)
New Jersey	17188 (15120, 19618)	26.6	392 (345, 448)	19.9	302 (266, 344)	1.7	172 (148, 199)	3.2 (49.1)	62 (51, 74)	4.4 (24.1)	25 (19, 31)	-2.5 (5.8)	44 (35, 54)	-4.9 (21)
New Mexico	4273 (3596, 4979)	83.7	416 (350, 484)	63.4	319 (268, 372)	32.8	185 (152, 220)	38.6 (65.1)	59 (47, 72)	31.3 (17.7)	28 (22, 36)	23 (6.7)	48 (38, 60)	20.9 (10.4)
New York	30797 (26215, 35887)	28.8	320 (272, 373)	23.7	248 (211, 289)	3.9	143 (118, 172)	5.6 (58.1)	49 (39, 61)	7.8 (27)	20 (16, 25)	-2.7 (4.2)	36 (27, 44)	-3.8 (10.7)

© Bowe B et al. *JAMA Network Open*

location	YLL number		YLL per 100,000 persons		Age-standardized YLL per 100,000 persons									
					All causes		Due to diabetes		Due to hypertension		Due to glomerulonephritis		Due to other causes	
	2016 (UI)	Percent change from 2002	2016 (UI)	Percent change from 2002	2016 (UI)	Percent change from 2002	2016 (UI)	Percent change from 2002 (PA*)	2016 (UI)	Percent change from 2002 (PA*)	2016 (UI)	Percent change from 2002 (PA*)	2016 (UI)	Percent change from 2002 (PA*)
North Carolina	24139 (21689, 26967)	73.4	491 (441, 548)	43.8	384 (345, 429)	14.9	223 (195, 253)	18.3 (69.4)	74 (62, 88)	14.6 (18.9)	32 (25, 39)	8.6 (5.1)	55 (45, 67)	6.3 (6.6)
North Dakota	1200 (1043, 1379)	56.8	303 (263, 348)	26.5	252 (218, 291)	26.3	142 (120, 167)	29.1 (60.8)	51 (42, 62)	26.4 (20.3)	22 (17, 27)	23.7 (8)	38 (30, 46)	18.1 (11)
Ohio	26989 (24151, 30300)	50.3	474 (424, 533)	46.6	354 (318, 397)	20.6	204 (178, 232)	25 (67.5)	69 (57, 84)	16.4 (16.1)	30 (24, 36)	16.4 (6.9)	51 (42, 63)	12.5 (9.5)
Oklahoma	8793 (7812, 9841)	73.6	454 (403, 508)	54.1	367 (327, 410)	35.2	213 (184, 242)	41.6 (65.5)	68 (56, 83)	31.1 (17)	31 (25, 38)	25.9 (6.8)	54 (44, 66)	23.5 (10.8)
Oregon	6110 (5491, 6853)	77	305 (274, 342)	53.8	218 (197, 245)	24	124 (107, 142)	28.2 (64.5)	46 (38, 55)	25 (21.6)	17 (14, 21)	14.8 (5.3)	31 (26, 38)	13.3 (8.6)
Pennsylvania	29465 (26449, 32828)	29.6	472 (423, 525)	23.6	332 (297, 370)	7.1	190 (166, 218)	10.2 (77.2)	67 (55, 80)	7.3 (20)	27 (22, 33)	0.8 (1)	48 (39, 58)	-0.9 (1.8)
Rhode Island	1688 (1461, 1962)	30.5	330 (286, 384)	30.6	243 (210, 282)	11.7	138 (115, 163)	14.4 (68.3)	49 (40, 61)	12.5 (21.6)	20 (16, 25)	6.5 (4.9)	35 (28, 44)	4 (5.3)

© Bowe B et al. *JAMA Network Open*

location	YLL number		YLL per 100,000 persons		Age-standardized YLL per 100,000 persons									
					All causes		Due to diabetes		Due to hypertension		Due to glomerulonephritis		Due to other causes	
	2016 (UI)	Percent change from 2002	2016 (UI)	Percent change from 2002	2016 (UI)	Percent change from 2002	2016 (UI)	Percent change from 2002 (PA*)	2016 (UI)	Percent change from 2002 (PA*)	2016 (UI)	Percent change from 2002 (PA*)	2016 (UI)	Percent change from 2002 (PA*)
South Carolina	13249 (11398, 15255)	66.1	553 (475, 636)	38.2	415 (358, 478)	9	242 (203, 286)	11.9 (75.6)	77 (63, 95)	10.1 (20.9)	35 (27, 44)	2.3 (2.3)	60 (48, 74)	0.7 (1.2)
South Dakota	1311 (1131, 1518)	52.9	301 (260, 348)	32.8	229 (198, 265)	18.4	129 (108, 152)	23.5 (68.9)	45 (37, 54)	14.2 (15.7)	20 (16, 25)	13.1 (6.6)	35 (28, 43)	9.9 (8.8)
Tennessee	15487 (13674, 17385)	81.9	479 (423, 537)	58.9	371 (330, 415)	30.1	216 (186, 247)	35.4 (65.7)	70 (58, 83)	29.8 (18.7)	31 (25, 38)	19.5 (5.9)	54 (44, 66)	18.3 (9.7)
Texas	55493 (48668, 62529)	110.3	401 (352, 452)	64.2	369 (325, 414)	35.3	216 (185, 248)	41 (65.3)	71 (58, 85)	33.8 (18.5)	30 (24, 37)	24.3 (6.1)	52 (42, 65)	22.8 (10.1)
Utah	3677 (3260, 4136)	93.3	242 (214, 272)	49.2	251 (223, 282)	23.9	141 (122, 162)	28.2 (64.4)	53 (43, 63)	23.6 (20.8)	21 (16, 25)	15.7 (5.8)	36 (29, 44)	13.7 (9.1)
Vermont	871 (764, 986)	56.7	283 (248, 321)	53.7	195 (171, 220)	18.5	109 (93, 127)	20.7 (61.4)	41 (33, 50)	18.4 (21)	16 (13, 20)	16.2 (7.5)	28 (23, 35)	12.1 (10.1)
Virginia	17603 (15757, 19715)	56.5	424 (380, 475)	34.9	342 (306, 381)	8.5	197 (171, 224)	11.6 (76.1)	69 (57, 82)	9.4 (22.1)	27 (22, 33)	1.5 (1.5)	49 (39, 59)	-0.1 (0.3)

© Bowe B et al. *JAMA Network Open*

location	YLL number		YLL per 100,000 persons		Age-standardized YLL per 100,000 persons									
					All causes		Due to diabetes		Due to hypertension		Due to glomerulonephritis		Due to other causes	
	2016 (UI)	Percent change from 2002	2016 (UI)	Percent change from 2002	2016 (UI)	Percent change from 2002	2016 (UI)	Percent change from 2002 (PA*)	2016 (UI)	Percent change from 2002 (PA*)	2016 (UI)	Percent change from 2002 (PA*)	2016 (UI)	Percent change from 2002 (PA*)
Washington	9155 (8120, 10250)	103.4	253 (225, 284)	69.6	199 (177, 223)	36.8	113 (97, 130)	41.6 (61.9)	42 (35, 51)	39.8 (22.4)	16 (12, 19)	23.9 (5.7)	28 (23, 34)	23.5 (10)
West Virginia	6062 (5387, 6804)	65.8	670 (596, 752)	61.6	459 (409, 514)	36.3	264 (230, 304)	41.2 (63.1)	88 (73, 105)	34.4 (18.3)	39 (31, 47)	28.4 (7.1)	68 (55, 82)	26.1 (11.4)
Wisconsin	10710 (9579, 11888)	56.1	373 (334, 414)	46.2	274 (245, 304)	18.2	153 (134, 173)	21.7 (64.9)	59 (48, 70)	18.5 (21.8)	22 (18, 27)	11.3 (5.4)	40 (31, 48)	9.2 (7.9)
Wyoming	833 (716, 966)	65.4	279 (240, 324)	39.6	221 (191, 257)	16.6	125 (105, 150)	22.5 (72.9)	44 (35, 53)	13.9 (16.9)	19 (15, 24)	7.5 (4.3)	33 (27, 41)	5.9 (5.9)

YLL: Years of Life Lost
UI is the uncertainty interval, it reflects the certainty of an estimate based on data availability, studies size and consistency across data sources
*Percentage attributed to overall rate is the proportion of overall age-standardized rate change attributed by each of the following four causes: diabetes, hypertension, glomerulonephritis and other causes

© Bowe B et al. *JAMA Network Open*

eTable6: Disability-adjusted life years (DALYs) due to CKD in the United States and by state and district in females and males.

eTable 6a: Number and age-standardized rate for overall DALY due to CKD and by cause in females, with percentage change from 2002 to 2016 for the Unites States and by state and district.

location	DALY number		DALY per 100,000 persons		Age-standardized DALY per 100,000 persons									
					All causes		Due to diabetes		Due to hypertension		Due to glomerulonephritis		Due to other causes	
	2016 (UI)	Percent change from 2002	2016 (UI)	Percent change from 2002	2016 (UI)	Percent change from 2002	2016 (UI)	Percent change from 2002 (PA*)	2016 (UI)	Percent change from 2002 (PA*)	2016 (UI)	Percent change from 2002 (PA*)	2016 (UI)	Percent change from 2002 (PA*)
United States	954857 (859005, 1055540)	46.5	583 (524, 644)	30.8	402 (359, 446)	17.5	214 (188, 242)	20.2 (60)	77 (63, 90)	21.7 (22.9)	46 (38, 56)	9.9 (7)	64 (52, 78)	10.3 (10)
Alabama	20442 (17706, 23318)	41	815 (706, 930)	30.2	558 (482, 637)	19.6	299 (252, 349)	23.5 (62.3)	102 (82, 125)	19.9 (18.6)	66 (52, 82)	12.4 (7.9)	90 (73, 111)	12.7 (11.1)
Alaska	1328 (1134, 1527)	68.9	382 (326, 439)	51.4	346 (297, 399)	20.7	182 (152, 214)	23.6 (58.6)	66 (53, 81)	25.7 (22.7)	42 (32, 52)	12.4 (7.8)	57 (45, 70)	12.8 (10.9)
Arizona	18164 (15660, 20807)	73.1	522 (450, 598)	34.6	358 (307, 410)	17.4	191 (160, 222)	20.2 (60.4)	67 (54, 81)	19.4 (20.4)	42 (33, 52)	11.4 (8.1)	58 (46, 71)	11.3 (11.1)
Arkansas	11431 (10005, 12820)	49.5	756 (662, 848)	36.6	515 (450, 579)	28.4	277 (237, 318)	32.7 (59.8)	96 (78, 115)	31.9 (20.3)	60 (48, 73)	18.3 (8.2)	83 (67, 101)	19.2 (11.7)
California	91965 (79881, 105553)	54	463 (402, 532)	36	342 (295, 393)	16.6	182 (155, 215)	18.8 (59.3)	65 (52, 79)	25.3 (27.1)	39 (31, 49)	6.6 (5)	54 (43, 67)	8.4 (8.6)

© Bowe B et al. *JAMA Network Open*

location	DALY number		DALY per 100,000 persons		Age-standardized DALY per 100,000 persons									
					All causes		Due to diabetes		Due to hypertension		Due to glomerulonephritis		Due to other causes	
	2016 (UI)	Percent change from 2002	2016 (UI)	Percent change from 2002	2016 (UI)	Percent change from 2002	2016 (UI)	Percent change from 2002 (PA*)	2016 (UI)	Percent change from 2002 (PA*)	2016 (UI)	Percent change from 2002 (PA*)	2016 (UI)	Percent change from 2002 (PA*)
Colorado	11107 (9672, 12641)	58.7	406 (353, 462)	29.1	302 (261, 346)	10.5	159 (134, 186)	12.2 (60.6)	59 (47, 71)	13 (23.9)	35 (28, 44)	6.2 (7.3)	49 (39, 60)	5.1 (8.3)
Connecticut	9092 (7746, 10339)	23.6	496 (423, 564)	19.6	316 (268, 361)	9.5	166 (139, 195)	10.8 (59.3)	61 (49, 74)	13.8 (27.2)	37 (29, 46)	4.3 (5.6)	51 (40, 63)	4.4 (7.9)
Delaware	3112 (2713, 3508)	63.8	634 (552, 714)	38.2	412 (359, 466)	18.7	219 (187, 252)	21.4 (59.6)	77 (62, 93)	21.5 (21)	49 (39, 60)	13 (8.6)	67 (54, 82)	11.7 (10.8)
District of Columbia	1866 (1592, 2136)	4.6	519 (443, 595)	-10.8	439 (373, 503)	-6.3	239 (200, 279)	-3.8 (31.6)	80 (63, 96)	0.4 (1.1)	50 (39, 61)	-15.6 (30.6)	70 (55, 87)	-13.7 (36.8)
Florida	61713 (53016, 70319)	60.2	590 (507, 673)	30.8	356 (305, 407)	20.7	191 (160, 224)	24.5 (61.4)	65 (52, 78)	23.7 (20.4)	42 (33, 52)	12 (7.4)	58 (46, 71)	12.7 (10.7)
Georgia	35818 (31273, 41080)	56.1	679 (593, 779)	28.2	516 (450, 594)	9.9	276 (236, 321)	11.9 (63.4)	99 (80, 122)	14.5 (27.2)	59 (46, 71)	2.9 (3.5)	82 (66, 101)	3.5 (5.9)
Hawaii	3801 (3293, 4307)	61	536 (464, 607)	40.5	344 (295, 392)	20.2	184 (156, 215)	23.3 (60.2)	64 (52, 77)	24.1 (21.5)	40 (32, 50)	12 (7.4)	55 (44, 67)	12.8 (10.9)

© Bowe B et al. *JAMA Network Open*

location	DALY number		DALY per 100,000 persons		Age-standardized DALY per 100,000 persons									
					All causes		Due to diabetes		Due to hypertension		Due to glomerulonephritis		Due to other causes	
	2016 (UI)	Percent change from 2002	2016 (UI)	Percent change from 2002	2016 (UI)	Percent change from 2002	2016 (UI)	Percent change from 2002 (PA*)	2016 (UI)	Percent change from 2002 (PA*)	2016 (UI)	Percent change from 2002 (PA*)	2016 (UI)	Percent change from 2002 (PA*)
Idaho	3942 (3388, 4532)	81.6	472 (405, 542)	45.1	342 (293, 395)	27.8	180 (150, 212)	30.4 (56.3)	67 (53, 81)	33 (22.4)	40 (32, 50)	20.1 (8.9)	55 (44, 68)	20 (12.3)
Illinois	38684 (34122, 43211)	25.1	592 (522, 661)	22.4	407 (358, 457)	8.8	216 (187, 247)	10.6 (62.4)	80 (65, 97)	15.1 (31.6)	46 (37, 56)	1.2 (1.6)	65 (53, 79)	2.2 (4.3)
Indiana	23128 (20026, 26516)	45.7	688 (596, 789)	35.6	474 (409, 546)	23.9	251 (214, 295)	26.6 (57.8)	93 (75, 114)	27.7 (22)	54 (43, 67)	16.7 (8.5)	76 (61, 93)	16.4 (11.7)
Iowa	7930 (6851, 9071)	40.7	503 (435, 576)	32.9	321 (275, 370)	27.8	169 (142, 199)	30.1 (56.1)	63 (50, 76)	34 (22.9)	37 (29, 46)	19.9 (8.8)	51 (41, 64)	19.9 (12.2)
Kansas	8655 (7507, 9916)	34.7	594 (515, 680)	26.7	407 (350, 469)	20.5	217 (183, 252)	23.9 (60.3)	79 (63, 96)	22.4 (20.8)	47 (37, 57)	12.9 (7.7)	65 (53, 80)	13.6 (11.2)
Kentucky	16640 (14718, 18693)	49.9	741 (655, 832)	39.2	504 (444, 568)	25.9	269 (232, 310)	29.6 (59)	97 (81, 116)	28.1 (20.4)	58 (46, 70)	18.2 (8.6)	81 (66, 97)	18.2 (12)
Louisiana	20814 (18403, 23216)	35.7	870 (769, 971)	31.4	634 (559, 709)	17.4	341 (295, 387)	19.9 (60.3)	118 (97, 142)	21.5 (22.2)	73 (58, 89)	10.6 (7.5)	102 (83, 123)	10.2 (10)

© Bowe B et al. *JAMA Network Open*

location	DALY number		DALY per 100,000 persons		Age-standardized DALY per 100,000 persons									
					All causes		Due to diabetes		Due to hypertension		Due to glomerulonephritis		Due to other causes	
	2016 (UI)	Percent change from 2002	2016 (UI)	Percent change from 2002	2016 (UI)	Percent change from 2002	2016 (UI)	Percent change from 2002 (PA*)	2016 (UI)	Percent change from 2002 (PA*)	2016 (UI)	Percent change from 2002 (PA*)	2016 (UI)	Percent change from 2002 (PA*)
Maine	4202 (3669, 4753)	36.3	621 (542, 702)	33.3	357 (311, 409)	14.4	187 (159, 218)	16.3 (58.3)	71 (57, 84)	17.5 (23.3)	42 (33, 51)	9.1 (7.7)	58 (46, 70)	9.1 (10.7)
Maryland	17040 (14964, 19306)	38.8	548 (481, 621)	25.5	384 (336, 435)	9.6	205 (174, 237)	11.6 (63.1)	73 (59, 88)	14.1 (26.6)	45 (36, 55)	3.2 (4.1)	62 (50, 75)	3.5 (6.2)
Massachusetts	17731 (15503, 20208)	22	505 (441, 575)	15.1	326 (282, 375)	7	172 (146, 201)	8.8 (65.1)	65 (52, 78)	10.2 (28.2)	37 (29, 46)	2.2 (3.8)	52 (41, 64)	1.2 (2.9)
Michigan	31605 (27732, 35502)	33.4	627 (550, 705)	34.8	410 (358, 463)	15.6	218 (187, 249)	17.8 (59.4)	79 (65, 94)	18.7 (22.4)	47 (38, 58)	9 (7.1)	66 (53, 80)	10.3 (11.1)
Minnesota	13642 (11829, 15420)	44.8	493 (427, 557)	32.1	329 (284, 375)	19.1	171 (144, 199)	22.2 (58.8)	67 (54, 81)	23.3 (23.9)	38 (30, 47)	10.8 (7)	53 (42, 65)	11.4 (10.3)
Mississippi	13982 (12016, 15856)	42.6	908 (780, 1029)	36.6	650 (558, 741)	22.5	350 (295, 405)	26.5 (61.4)	118 (95, 144)	26.5 (20.7)	77 (61, 93)	12.7 (7.2)	105 (84, 129)	13.8 (10.7)
Missouri	20256 (17788, 22638)	43.2	654 (574, 730)	34.4	432 (376, 486)	23.3	230 (198, 267)	27.4 (60.5)	83 (68, 98)	25.4 (20.5)	50 (39, 62)	14.5 (7.7)	69 (56, 84)	15.3 (11.3)

© Bowe B et al. *JAMA Network Open*

location	DALY number		DALY per 100,000 persons		Age-standardized DALY per 100,000 persons									
					All causes		Due to diabetes		Due to hypertension		Due to glomerulonephritis		Due to other causes	
	2016 (UI)	Percent change from 2002	2016 (UI)	Percent change from 2002	2016 (UI)	Percent change from 2002	2016 (UI)	Percent change from 2002 (PA*)	2016 (UI)	Percent change from 2002 (PA*)	2016 (UI)	Percent change from 2002 (PA*)	2016 (UI)	Percent change from 2002 (PA*)
Montana	2490 (2132, 2867)	46.8	483 (414, 556)	30	313 (266, 361)	17.1	165 (138, 195)	20.1 (60.4)	60 (48, 73)	19.9 (21.7)	37 (29, 46)	10.6 (7.8)	51 (41, 63)	9.9 (10)
Nebraska	5242 (4594, 5905)	38.7	550 (482, 619)	27.2	376 (327, 427)	21.3	199 (169, 231)	24.3 (58.8)	74 (60, 88)	26.5 (23.4)	43 (34, 53)	12.5 (7.3)	60 (48, 73)	13 (10.5)
Nevada	8043 (7054, 9039)	69.8	550 (482, 618)	23.8	402 (352, 454)	6.6	215 (183, 248)	9.1 (71.8)	77 (63, 91)	7.4 (21.3)	46 (37, 57)	2.3 (4.2)	65 (52, 78)	1 (2.7)
New Hampshire	3342 (2904, 3792)	46.6	496 (431, 562)	39.3	311 (269, 356)	15.4	163 (138, 189)	16.5 (55.4)	61 (48, 74)	17.9 (22.2)	37 (29, 46)	13.6 (10.7)	51 (40, 62)	10.6 (11.6)
New Jersey	24698 (21506, 28031)	20.7	539 (469, 612)	15.7	354 (305, 402)	5.5	188 (159, 217)	7.3 (67.1)	69 (55, 82)	9.3 (30.8)	41 (32, 51)	0.1 (0.2)	57 (45, 69)	-0.6 (1.9)
New Mexico	6166 (5315, 7090)	73.4	590 (509, 679)	56.5	405 (348, 468)	31.7	216 (182, 257)	35.5 (58.1)	75 (60, 91)	33.5 (19.4)	48 (38, 59)	24.6 (9.8)	66 (53, 80)	23.4 (12.8)
New York	49601 (42620, 57366)	24.7	487 (418, 563)	21.3	328 (280, 382)	10.2	175 (146, 206)	11.6 (59.6)	62 (50, 76)	15.1 (27)	39 (30, 49)	4.9 (5.9)	53 (42, 66)	4.5 (7.4)

© Bowe B et al. *JAMA Network Open*

location	DALY number		DALY per 100,000 persons		Age-standardized DALY per 100,000 persons									
					All causes		Due to diabetes		Due to hypertension		Due to glomerulonephritis		Due to other causes	
	2016 (UI)	Percent change from 2002	2016 (UI)	Percent change from 2002	2016 (UI)	Percent change from 2002	2016 (UI)	Percent change from 2002 (PA*)	2016 (UI)	Percent change from 2002 (PA*)	2016 (UI)	Percent change from 2002 (PA*)	2016 (UI)	Percent change from 2002 (PA*)
North Carolina	36091 (31922, 40346)	62.3	696 (615, 778)	33	478 (421, 534)	18.4	257 (223, 294)	21.3 (60.8)	90 (74, 108)	20.3 (20.6)	55 (44, 66)	12.8 (8.3)	76 (61, 91)	11.3 (10.4)
North Dakota	1809 (1562, 2067)	37.1	483 (417, 552)	16.8	340 (292, 390)	21.9	179 (151, 210)	24.9 (58.5)	65 (51, 78)	24.4 (20.7)	41 (32, 50)	14.4 (8.3)	56 (44, 68)	15.8 (12.4)
Ohio	40070 (35665, 44685)	34	677 (603, 755)	32.7	442 (393, 496)	19	236 (204, 270)	21.7 (59.3)	85 (70, 103)	22.7 (22.3)	51 (41, 62)	12.4 (7.9)	70 (57, 86)	11.8 (10.5)
Oklahoma	12892 (11437, 14421)	55.3	653 (579, 730)	39.1	466 (413, 522)	33.2	249 (215, 288)	37.8 (58.8)	87 (72, 104)	36.2 (19.9)	54 (44, 66)	23.6 (8.9)	75 (62, 90)	23.7 (12.4)
Oregon	9957 (8650, 11248)	55.2	488 (424, 551)	34.5	316 (271, 359)	18.9	167 (141, 194)	20.6 (56.8)	62 (50, 75)	23.2 (23.3)	36 (29, 45)	13.6 (8.7)	50 (40, 62)	12.4 (11.1)
Pennsylvania	42700 (37464, 47841)	22.4	655 (575, 734)	19.3	399 (349, 451)	11.9	211 (181, 243)	13.9 (60.5)	78 (63, 93)	16 (25.3)	46 (37, 56)	6.1 (6.2)	64 (51, 78)	5.7 (8.1)
Rhode Island	2733 (2335, 3141)	25.4	502 (429, 577)	27.1	317 (269, 365)	17	168 (141, 198)	18.9 (57.8)	61 (49, 75)	22.9 (24.9)	37 (28, 46)	9.2 (6.7)	51 (40, 63)	10.6 (10.6)

© Bowe B et al. *JAMA Network Open*

location	DALY number		DALY per 100,000 persons		Age-standardized DALY per 100,000 persons									
					All causes		Due to diabetes		Due to hypertension		Due to glomerulonephritis		Due to other causes	
	2016 (UI)	Percent change from 2002	2016 (UI)	Percent change from 2002	2016 (UI)	Percent change from 2002	2016 (UI)	Percent change from 2002 (PA*)	2016 (UI)	Percent change from 2002 (PA*)	2016 (UI)	Percent change from 2002 (PA*)	2016 (UI)	Percent change from 2002 (PA*)
South Carolina	19466 (16840, 22329)	63.2	768 (664, 881)	35.7	514 (441, 591)	17.1	276 (233, 324)	19.7 (60.5)	98 (78, 118)	21.8 (23.3)	59 (46, 73)	9.5 (6.8)	82 (66, 99)	9.5 (9.4)
South Dakota	2201 (1876, 2511)	46.9	512 (437, 585)	30.8	346 (295, 397)	24.6	183 (153, 214)	28.1 (58.8)	64 (52, 78)	28.2 (20.8)	42 (33, 51)	15.6 (8.2)	57 (45, 71)	17.2 (12.2)
Tennessee	22976 (20179, 25722)	61.3	677 (594, 758)	41.2	465 (408, 522)	27.3	248 (214, 283)	31.5 (59.7)	88 (71, 106)	32.1 (21.5)	54 (43, 66)	16 (7.5)	74 (61, 90)	17.9 (11.3)
Texas	80873 (71014, 91196)	84.3	577 (507, 651)	43.9	466 (408, 526)	29	251 (216, 290)	32.4 (58.6)	89 (73, 107)	34.7 (21.9)	53 (42, 65)	18.8 (8)	74 (59, 90)	19.6 (11.5)
Utah	5871 (5132, 6648)	78.6	390 (341, 441)	38.3	360 (313, 408)	23.3	189 (160, 220)	25.1 (55.8)	72 (59, 86)	29 (23.8)	41 (32, 50)	16.9 (8.7)	57 (46, 70)	16 (11.7)
Vermont	1553 (1340, 1770)	34.2	490 (423, 559)	32.1	301 (258, 345)	12.3	157 (132, 184)	13.9 (58.3)	58 (47, 70)	14.9 (23)	36 (29, 45)	9 (9.1)	49 (39, 61)	6.9 (9.6)
Virginia	26202 (22967, 29284)	50	613 (537, 685)	30.2	430 (376, 483)	14.3	229 (197, 265)	16.9 (61.5)	84 (69, 100)	18 (23.7)	49 (39, 60)	6.9 (5.8)	68 (55, 84)	7.7 (9.1)

© Bowe B et al. *JAMA Network Open*

location	DALY number		DALY per 100,000 persons		Age-standardized DALY per 100,000 persons									
					All causes		Due to diabetes		Due to hypertension		Due to glomerulonephritis		Due to other causes	
	2016 (UI)	Percent change from 2002	2016 (UI)	Percent change from 2002	2016 (UI)	Percent change from 2002	2016 (UI)	Percent change from 2002 (PA*)	2016 (UI)	Percent change from 2002 (PA*)	2016 (UI)	Percent change from 2002 (PA*)	2016 (UI)	Percent change from 2002 (PA*)
Washington	15790 (13602, 18012)	70.8	438 (377, 500)	43.7	306 (261, 350)	25.8	162 (135, 189)	28.4 (57)	60 (48, 72)	32 (23.2)	35 (28, 44)	16.7 (8)	49 (39, 60)	17.9 (11.8)
West Virginia	8073 (7174, 9088)	38	871 (774, 981)	37.4	538 (478, 610)	28.8	287 (249, 330)	32.6 (58.6)	101 (83, 122)	30.6 (19.7)	63 (51, 76)	21.3 (9.2)	87 (71, 105)	20.8 (12.4)
Wisconsin	16696 (14718, 18743)	45.1	575 (507, 646)	37.2	370 (324, 418)	22.6	195 (166, 224)	25.2 (57.4)	74 (60, 88)	27.9 (23.6)	42 (34, 52)	14.1 (7.7)	59 (47, 72)	15 (11.3)
Wyoming	1232 (1056, 1425)	44.4	431 (369, 499)	25	314 (267, 363)	15.6	167 (139, 196)	18.1 (60.3)	59 (47, 72)	18.6 (21.9)	38 (30, 46)	9.7 (7.9)	51 (41, 63)	9 (10)

DALY: Disability Adjusted Life Year
UI is the uncertainty interval, it reflects the certainty of an estimate based on data availability, studies size and consistency across data sources
*Percentage attributed to overall rate is the proportion of overall age-standardized rate change attributed by each of the following four causes: diabetes, hypertension, glomerulonephritis and other causes

© Bowe B et al. *JAMA Network Open*

eTable 6b: Number and age-standardized rate for overall DALY due to CKD and by cause in males, with percentage change from 2002 to 2016 for the Unites States and by state and district.

location	DALY number		DALY per 100,000 persons		Age-standardized DALY per 100,000 persons									
					All causes		Due to diabetes		Due to hypertension		Due to glomerulonephritis		Due to other causes	
	2016 (UI)	Percent change from 2002	2016 (UI)	Percent change from 2002	2016 (UI)	Percent change from 2002	2016 (UI)	Percent change from 2002 (PA*)	2016 (UI)	Percent change from 2002 (PA*)	2016 (UI)	Percent change from 2002 (PA*)	2016 (UI)	Percent change from 2002 (PA*)
United States	981097 (889245, 1073356)	59	617 (559, 675)	41.3	487 (440, 534)	18.3	280 (248, 312)	21.7 (66.3)	91 (76, 107)	19.6 (19.8)	44 (35, 53)	10.8 (5.7)	72 (60, 87)	9.5 (8.3)
Alabama	20041 (17336, 22865)	51.9	852 (737, 972)	39.9	662 (573, 750)	17.6	383 (327, 441)	21.8 (69.1)	117 (97, 143)	14.5 (15)	61 (48, 75)	12.8 (7)	100 (80, 122)	9.7 (8.9)
Alaska	1462 (1240, 1678)	81.6	377 (320, 433)	56.8	374 (321, 427)	24.3	211 (176, 246)	27.5 (62.4)	70 (58, 85)	28.9 (21.6)	35 (28, 44)	15.3 (6.4)	57 (46, 69)	14 (9.6)
Arizona	19178 (16715, 21659)	81.1	559 (487, 631)	42.2	424 (368, 479)	16.8	245 (210, 283)	21.5 (71.1)	76 (62, 91)	15.6 (16.8)	39 (31, 47)	7.6 (4.5)	64 (51, 78)	7.8 (7.6)
Arkansas	11295 (9997, 12671)	59.6	773 (684, 867)	44.3	596 (529, 669)	25.9	345 (300, 391)	30.8 (66.1)	107 (89, 128)	26.9 (18.5)	55 (44, 66)	15.5 (6)	90 (74, 109)	14.9 (9.5)
California	97062 (83648, 110459)	71.6	495 (427, 564)	52.5	417 (359, 475)	24.5	241 (205, 280)	28.2 (64.6)	77 (63, 94)	29.8 (21.7)	37 (29, 46)	13.1 (5.3)	61 (50, 76)	12.8 (8.5)

© Bowe B et al. *JAMA Network Open*

location	DALY number		DALY per 100,000 persons		Age-standardized DALY per 100,000 persons									
					All causes		Due to diabetes		Due to hypertension		Due to glomerulonephritis		Due to other causes	
	2016 (UI)	Percent change from 2002	2016 (UI)	Percent change from 2002	2016 (UI)	Percent change from 2002	2016 (UI)	Percent change from 2002 (PA*)	2016 (UI)	Percent change from 2002 (PA*)	2016 (UI)	Percent change from 2002 (PA*)	2016 (UI)	Percent change from 2002 (PA*)
Colorado	11848 (10228, 13405)	78.5	428 (369, 484)	45.8	364 (315, 411)	15.2	206 (175, 239)	18.9 (68.1)	70 (58, 85)	14.5 (18.5)	33 (26, 41)	8.9 (5.6)	55 (44, 67)	7.4 (7.8)
Connecticut	9551 (8284, 10969)	35.4	547 (475, 629)	29.7	407 (353, 466)	11.6	231 (196, 270)	13.8 (66.3)	79 (65, 96)	13.5 (22.1)	37 (29, 45)	5.9 (4.9)	60 (48, 75)	4.9 (6.7)
Delaware	3179 (2786, 3568)	68	688 (603, 773)	42.5	505 (443, 566)	15.6	290 (249, 330)	19.5 (69.4)	91 (75, 109)	15.1 (17.6)	47 (37, 58)	9.6 (6)	76 (62, 93)	6.6 (6.9)
District of Columbia	2045 (1775, 2343)	7.4	628 (545, 719)	-9.7	600 (523, 686)	-10.3	354 (304, 409)	-6.6 (36.3)	105 (87, 127)	-3.4 (5.4)	53 (41, 65)	-24.2 (24.4)	88 (71, 108)	-21 (33.9)
Florida	68533 (59639, 78095)	73.5	686 (597, 781)	41.3	457 (395, 519)	24	264 (226, 303)	28.6 (66.4)	82 (68, 98)	25 (18.6)	42 (33, 51)	14.3 (5.9)	68 (55, 84)	13.4 (9.1)
Georgia	35263 (30362, 40272)	73.5	705 (607, 805)	45.1	615 (531, 700)	14.5	356 (302, 412)	17.3 (67.3)	114 (93, 138)	16.1 (20.2)	55 (43, 67)	8.7 (5.6)	91 (74, 111)	6.3 (6.9)
Hawaii	4150 (3640, 4698)	66.8	568 (498, 643)	43.1	431 (378, 490)	24.6	249 (216, 288)	28.3 (64.4)	79 (65, 94)	26.1 (19.2)	39 (31, 48)	16.2 (6.4)	64 (52, 79)	15.2 (10)

© Bowe B et al. *JAMA Network Open*

location	DALY number		DALY per 100,000 persons		Age-standardized DALY per 100,000 persons									
					All causes		Due to diabetes		Due to hypertension		Due to glomerulonephritis		Due to other causes	
	2016 (UI)	Percent change from 2002	2016 (UI)	Percent change from 2002	2016 (UI)	Percent change from 2002	2016 (UI)	Percent change from 2002 (PA*)	2016 (UI)	Percent change from 2002 (PA*)	2016 (UI)	Percent change from 2002 (PA*)	2016 (UI)	Percent change from 2002 (PA*)
Idaho	4143 (3550, 4727)	93.2	493 (422, 563)	54.1	396 (339, 452)	27.4	224 (188, 261)	31.1 (62.2)	76 (62, 92)	30 (20.6)	37 (29, 46)	18.8 (6.8)	60 (48, 73)	17.5 (10.4)
Illinois	40535 (35843, 45093)	37.7	643 (568, 715)	34.2	517 (456, 577)	10.5	296 (259, 336)	13.2 (70.1)	99 (82, 118)	14.2 (25.1)	46 (37, 55)	2.7 (2.4)	76 (61, 93)	1.6 (2.4)
Indiana	22999 (19823, 26122)	56.6	704 (607, 800)	44.9	566 (488, 643)	21.6	322 (270, 372)	25 (64)	109 (88, 132)	21.1 (18.8)	51 (40, 63)	16.4 (7.2)	85 (69, 104)	13.6 (10.1)
Iowa	7967 (6922, 9017)	59.2	512 (445, 579)	47.3	384 (333, 436)	30.7	218 (187, 254)	34.5 (61.9)	75 (61, 89)	34.3 (21.1)	35 (27, 43)	20.6 (6.6)	57 (46, 70)	19.8 (10.5)
Kansas	8808 (7652, 9984)	51.1	607 (528, 689)	39.8	487 (424, 552)	22.2	277 (235, 322)	26.2 (65.1)	93 (76, 113)	21.3 (18.5)	44 (35, 55)	15.7 (6.8)	73 (58, 88)	13.3 (9.6)
Kentucky	16846 (14900, 18665)	63.6	773 (684, 857)	50.4	607 (535, 671)	24.7	348 (299, 395)	29.1 (65.1)	114 (94, 137)	23 (17.7)	54 (44, 66)	17.6 (6.8)	90 (74, 109)	16 (10.4)
Louisiana	20401 (18213, 22584)	44	892 (796, 987)	37.4	741 (661, 819)	15.3	428 (373, 482)	18.8 (68.9)	134 (112, 158)	17.6 (20.3)	67 (54, 81)	6.6 (4.2)	111 (92, 134)	6.1 (6.5)

© Bowe B et al. *JAMA Network Open*

location	DALY number		DALY per 100,000 persons		Age-standardized DALY per 100,000 persons									
					All causes		Due to diabetes		Due to hypertension		Due to glomerulonephritis		Due to other causes	
	2016 (UI)	Percent change from 2002	2016 (UI)	Percent change from 2002	2016 (UI)	Percent change from 2002	2016 (UI)	Percent change from 2002 (PA*)	2016 (UI)	Percent change from 2002 (PA*)	2016 (UI)	Percent change from 2002 (PA*)	2016 (UI)	Percent change from 2002 (PA*)
Maine	4348 (3833, 4859)	54.3	668 (589, 747)	49.4	439 (387, 490)	18.3	247 (214, 282)	20.7 (62.4)	85 (70, 101)	17.4 (18.6)	40 (32, 49)	14.6 (7.6)	66 (54, 80)	13.3 (11.5)
Maryland	17378 (15438, 19524)	43	595 (528, 668)	28.4	481 (427, 539)	8.1	277 (241, 315)	10.6 (73.4)	88 (73, 106)	11.7 (25.7)	44 (35, 54)	0.1 (0.1)	72 (59, 88)	0.4 (0.8)
Massachusetts	19612 (17226, 22083)	36.1	591 (519, 666)	26.9	452 (396, 510)	8.6	256 (219, 294)	10.3 (66.9)	90 (74, 108)	10.4 (23.7)	40 (31, 49)	4.8 (5.1)	66 (53, 81)	2.4 (4.3)
Michigan	32089 (28330, 35878)	45.8	658 (581, 736)	46.9	495 (437, 553)	17.8	282 (245, 319)	20.9 (65.4)	94 (78, 113)	18.4 (19.5)	45 (36, 55)	11.7 (6.3)	74 (60, 89)	9.6 (8.7)
Minnesota	14260 (12457, 16097)	58	520 (455, 587)	43.3	404 (352, 456)	17.5	226 (194, 261)	20.8 (64.7)	81 (66, 98)	17.3 (19.9)	36 (28, 45)	11.5 (6.2)	60 (49, 74)	10.2 (9.3)
Mississippi	13487 (11604, 15382)	54.9	931 (801, 1061)	47.9	758 (654, 862)	22.8	442 (374, 510)	27 (66.8)	134 (110, 161)	22.5 (17.5)	69 (54, 84)	15.1 (6.4)	113 (92, 140)	13.1 (9.3)
Missouri	20789 (18416, 22978)	55.5	696 (617, 770)	43.9	532 (472, 589)	21.9	305 (265, 347)	26.1 (65.9)	99 (84, 118)	21.7 (18.5)	48 (39, 59)	14.2 (6.2)	80 (65, 97)	12.7 (9.3)

© Bowe B et al. *JAMA Network Open*

location	DALY number		DALY per 100,000 persons		Age-standardized DALY per 100,000 persons									
					All causes		Due to diabetes		Due to hypertension		Due to glomerulonephritis		Due to other causes	
	2016 (UI)	Percent change from 2002	2016 (UI)	Percent change from 2002	2016 (UI)	Percent change from 2002	2016 (UI)	Percent change from 2002 (PA*)	2016 (UI)	Percent change from 2002 (PA*)	2016 (UI)	Percent change from 2002 (PA*)	2016 (UI)	Percent change from 2002 (PA*)
Montana	2596 (2196, 2980)	57.9	498 (421, 571)	37.5	356 (301, 408)	15.3	201 (167, 235)	19.5 (69.6)	66 (53, 79)	12.7 (15.8)	34 (27, 42)	8.8 (5.8)	55 (44, 66)	8.2 (8.8)
Nebraska	5237 (4641, 5860)	49.8	550 (488, 616)	34.4	441 (390, 494)	19.3	250 (214, 287)	22.6 (64.3)	85 (70, 102)	20 (19.9)	40 (32, 49)	13.6 (6.7)	66 (53, 80)	10.9 (9.1)
Nevada	9876 (8703, 11042)	71.5	672 (593, 752)	28.5	528 (464, 590)	5.3	304 (265, 347)	8.4 (81.7)	97 (80, 115)	3.8 (12.2)	48 (38, 58)	1.2 (1.9)	79 (64, 96)	-1.5 (4.2)
New Hampshire	3542 (3082, 4014)	67.7	537 (468, 609)	58.3	382 (331, 433)	21	214 (183, 248)	23.3 (61)	74 (61, 89)	21.5 (19.8)	36 (29, 44)	18.4 (8.3)	58 (47, 71)	14.2 (10.8)
New Jersey	26439 (23186, 29879)	31.4	604 (529, 682)	24.4	470 (413, 532)	6.8	268 (231, 314)	8.5 (69.9)	91 (75, 110)	9.2 (25.4)	42 (33, 52)	3.1 (4.2)	70 (56, 85)	0.2 (0.5)
New Mexico	6518 (5624, 7424)	76.9	634 (547, 722)	57.4	490 (421, 559)	29	281 (239, 328)	33.4 (63.9)	88 (70, 106)	29.4 (18.1)	46 (36, 57)	19.7 (6.9)	75 (61, 92)	19.5 (11.1)
New York	50743 (43762, 57880)	34.3	527 (455, 601)	29	414 (357, 473)	10	238 (201, 276)	11.9 (67.4)	77 (63, 93)	13 (23.7)	37 (29, 46)	4 (3.8)	62 (49, 75)	3.2 (5.1)

© Bowe B et al. *JAMA Network Open*

location	DALY number		DALY per 100,000 persons		Age-standardized DALY per 100,000 persons									
					All causes		Due to diabetes		Due to hypertension		Due to glomerulonephritis		Due to other causes	
	2016 (UI)	Percent change from 2002	2016 (UI)	Percent change from 2002	2016 (UI)	Percent change from 2002	2016 (UI)	Percent change from 2002 (PA*)	2016 (UI)	Percent change from 2002 (PA*)	2016 (UI)	Percent change from 2002 (PA*)	2016 (UI)	Percent change from 2002 (PA*)
North Carolina	34971 (31115, 38815)	72.7	711 (633, 789)	43.2	562 (499, 623)	16.3	325 (285, 365)	19.5 (67.2)	104 (87, 124)	17.2 (19.4)	50 (40, 60)	9.9 (5.7)	83 (68, 101)	7.9 (7.7)
North Dakota	1972 (1700, 2234)	55	498 (429, 564)	25	418 (361, 475)	24.4	236 (200, 273)	27.2 (61.4)	79 (65, 95)	25.7 (19.7)	39 (31, 48)	20.2 (8)	63 (51, 77)	16.4 (10.9)
Ohio	39909 (35535, 44272)	47.9	701 (625, 778)	44.3	531 (472, 588)	20.3	305 (266, 344)	24 (65.8)	99 (82, 119)	18.4 (17.2)	48 (38, 58)	15 (7)	79 (65, 96)	12.9 (10.1)
Oklahoma	12844 (11458, 14276)	66.5	663 (591, 737)	47.8	541 (482, 600)	30.9	311 (270, 351)	36.1 (64.8)	98 (81, 117)	29.2 (17.4)	50 (39, 61)	21.4 (6.9)	82 (67, 99)	20.8 (11)
Oregon	10248 (8942, 11525)	71.4	512 (447, 576)	49	374 (324, 419)	22.5	212 (181, 243)	26.2 (64.1)	72 (60, 88)	24.6 (20.8)	33 (27, 41)	14.6 (6.2)	55 (44, 68)	12.4 (8.9)
Pennsylvania	43706 (38685, 48880)	32	700 (619, 782)	25.9	501 (443, 561)	10.1	286 (249, 325)	12.9 (70.8)	96 (80, 115)	10.8 (20.3)	45 (36, 54)	4.4 (4.1)	74 (60, 90)	3.1 (4.8)
Rhode Island	2769 (2387, 3156)	32.6	541 (467, 617)	32.6	405 (349, 463)	14.5	230 (194, 265)	17.2 (65.8)	77 (63, 93)	15.4 (20.1)	37 (29, 45)	9.5 (6.3)	61 (49, 75)	7 (7.8)

© Bowe B et al. *JAMA Network Open*

location	DALY number		DALY per 100,000 persons		Age-standardized DALY per 100,000 persons									
					All causes		Due to diabetes		Due to hypertension		Due to glomerulonephritis		Due to other causes	
	2016 (UI)	Percent change from 2002	2016 (UI)	Percent change from 2002	2016 (UI)	Percent change from 2002	2016 (UI)	Percent change from 2002 (PA*)	2016 (UI)	Percent change from 2002 (PA*)	2016 (UI)	Percent change from 2002 (PA*)	2016 (UI)	Percent change from 2002 (PA*)
South Carolina	18806 (16408, 21348)	68.4	784 (684, 890)	40.1	595 (520, 673)	11.9	344 (295, 398)	14.7 (69.7)	108 (88, 130)	13.7 (20.6)	54 (42, 65)	5.8 (4.7)	89 (71, 108)	3.7 (5)
South Dakota	2196 (1906, 2491)	53.6	504 (438, 572)	33.4	389 (338, 442)	19	219 (186, 254)	23.1 (66.2)	72 (59, 86)	17.4 (17.3)	37 (30, 45)	13 (6.9)	60 (48, 73)	11.1 (9.7)
Tennessee	22544 (19975, 25260)	75.1	697 (617, 781)	53	545 (484, 612)	27	315 (273, 358)	31.5 (65.1)	100 (82, 117)	28.5 (19)	49 (39, 60)	16.8 (6.1)	81 (67, 100)	16.2 (9.8)
Texas	81993 (72109, 92415)	100.3	593 (521, 668)	56.4	546 (481, 614)	31.2	317 (275, 362)	35.8 (64.4)	101 (83, 122)	32.1 (18.8)	48 (38, 59)	20.4 (6.3)	80 (65, 98)	20.6 (10.5)
Utah	6190 (5442, 6988)	88.1	407 (358, 459)	45.2	419 (369, 473)	22.1	237 (202, 272)	25.9 (64.3)	81 (67, 98)	23.2 (20.2)	38 (30, 47)	13.2 (5.8)	63 (51, 77)	13.3 (9.7)
Vermont	1529 (1337, 1736)	53.3	497 (435, 564)	50.4	347 (303, 394)	18.6	196 (169, 227)	20.9 (62.1)	67 (55, 81)	20 (20.5)	32 (25, 40)	13.7 (7.2)	52 (42, 64)	11.8 (10.2)
Virginia	26346 (23267, 29515)	58	635 (561, 712)	36.1	516 (455, 577)	11.8	296 (258, 337)	14.5 (69)	99 (83, 117)	13.2 (21.2)	45 (36, 56)	5.9 (4.7)	76 (62, 92)	3.9 (5.2)

© Bowe B et al. *JAMA Network Open*

location	DALY number		DALY per 100,000 persons		Age-standardized DALY per 100,000 persons									
					All causes		Due to diabetes		Due to hypertension		Due to glomerulonephritis		Due to other causes	
	2016 (UI)	Percent change from 2002	2016 (UI)	Percent change from 2002	2016 (UI)	Percent change from 2002	2016 (UI)	Percent change from 2002 (PA*)	2016 (UI)	Percent change from 2002 (PA*)	2016 (UI)	Percent change from 2002 (PA*)	2016 (UI)	Percent change from 2002 (PA*)
Washington	16114 (14066, 18206)	88.8	446 (389, 504)	57.4	355 (309, 401)	30.3	202 (171, 231)	34 (61.9)	69 (56, 82)	34.3 (21.3)	32 (25, 39)	19.1 (6.2)	53 (43, 65)	19.9 (10.6)
West Virginia	8434 (7496, 9390)	59.6	932 (829, 1038)	55.6	646 (575, 718)	32.3	370 (324, 422)	36.7 (63)	120 (99, 142)	32 (18.4)	59 (47, 71)	23.9 (7.2)	97 (80, 117)	22.8 (11.4)
Wisconsin	16902 (14904, 18882)	54.3	589 (520, 658)	44.6	439 (388, 492)	19	247 (213, 281)	22.3 (64.2)	87 (72, 103)	20.3 (21)	39 (31, 48)	11.6 (5.8)	66 (53, 80)	10.6 (9)
Wyoming	1402 (1200, 1600)	63.9	470 (402, 536)	38.3	374 (320, 426)	17.8	212 (178, 245)	22.1 (67.9)	70 (56, 84)	17.1 (18)	35 (28, 44)	9.7 (5.5)	57 (46, 69)	9.3 (8.6)

DALY: Disability Adjusted Life Year
UI is the uncertainty interval, it reflects the certainty of an estimate based on data availability, studies size and consistency across data sources
*Percentage attributed to overall rate is the proportion of overall age-standardized rate change attributed by each of the following four causes: diabetes, hypertension, glomerulonephritis and other causes

© Bowe B et al. *JAMA Network Open*

eTable 7: Death due to CKD in the United States and by state and district in females and males.

eTable 7a: Number and age-standardized rate for overall Death due to CKD and by cause in females, with percentage change from 2002 to 2016 for the Unites States and by state and district.

Location	Death number		Death per 100,000 persons		Age-standardized Death per 100,000 persons									
					All causes		Due to diabetes		Due to hypertension		Due to glomerulonephritis		Due to other causes	
	2016 (UI)	Percent change from 2002	2016 (UI)	Percent change from 2002	2016 (UI)	Percent change from 2002	2016 (UI)	Percent change from 2002 (PA*)	2016 (UI)	Percent change from 2002 (PA*)	2016 (UI)	Percent change from 2002 (PA*)	2016 (UI)	Percent change from 2002 (PA*)
United States	41606 (40050, 43173)	51.5	25 (24, 26)	35.4	14 (14, 15)	18	7 (6, 8)	19.3 (51.6)	4 (3, 5)	21.3 (33.1)	1 (1, 1)	11.4 (5.6)	2 (2, 2)	11.7 (9.7)
Alabama	889 (762, 1033)	33	35 (30, 41)	22.8	20 (17, 24)	12.3	10 (8, 12)	15.8 (62.1)	6 (4, 7)	10.3 (23.4)	2 (1, 2)	8.4 (6.2)	3 (2, 4)	6.8 (8.3)
Alaska	43 (36, 51)	111.2	12 (10, 15)	89.2	12 (10, 14)	28.9	6 (4, 7)	28.4 (46.9)	3 (3, 4)	36.5 (35.2)	1 (1, 1)	20.3 (6.4)	2 (1, 2)	22 (11.5)
Arizona	703 (614, 799)	77	20 (18, 23)	37.7	11 (10, 13)	13.6	6 (5, 7)	15.6 (55.1)	3 (3, 4)	15 (30.8)	1 (1, 1)	8.4 (5.4)	2 (1, 2)	7.9 (8.7)
Arkansas	529 (469, 593)	53.9	35 (31, 39)	40.7	19 (17, 22)	32.2	9 (8, 11)	34.9 (52.2)	5 (4, 6)	34 (29.1)	2 (1, 2)	24.8 (7)	3 (2, 3)	25 (11.8)
California	3644 (3143, 4206)	81.3	18 (16, 21)	60.2	11 (10, 13)	29	5 (5, 7)	28.4 (47.9)	3 (3, 4)	37.6 (35.4)	1 (1, 1)	18.9 (5.8)	2 (1, 2)	21.1 (10.9)

© Bowe B et al. *JAMA Network Open*

Location	Death number		Death per 100,000 persons		Age-standardized Death per 100,000 persons									
					All causes		Due to diabetes		Due to hypertension		Due to glomerulonephritis		Due to other causes	
	2016 (UI)	Percent change from 2002	2016 (UI)	Percent change from 2002	2016 (UI)	Percent change from 2002	2016 (UI)	Percent change from 2002 (PA*)	2016 (UI)	Percent change from 2002 (PA*)	2016 (UI)	Percent change from 2002 (PA*)	2016 (UI)	Percent change from 2002 (PA*)
Colorado	440 (393, 497)	62.4	16 (14, 18)	32.1	10 (9, 12)	6.6	5 (4, 6)	7.1 (50.6)	3 (3, 4)	10 (44.8)	1 (1, 1)	1.2 (1.5)	1 (1, 2)	1.4 (3.1)
Connecticut	406 (347, 469)	28.6	22 (19, 26)	24.5	10 (9, 12)	7.5	5 (4, 6)	6.4 (41.3)	3 (2, 4)	13.9 (52.6)	1 (1, 1)	1.3 (1.5)	1 (1, 2)	2.3 (4.6)
Delaware	130 (116, 146)	70.9	27 (24, 30)	44.3	14 (12, 16)	18	7 (6, 8)	19.2 (51.3)	4 (3, 5)	21.1 (32.7)	1 (1, 2)	12.1 (6)	2 (2, 2)	11.9 (10)
District of Columbia	74 (63, 86)	1	21 (18, 24)	-13.9	15 (13, 18)	-8.5	8 (6, 9)	-7.3 (43.5)	4 (3, 5)	-4.3 (12.4)	1 (1, 2)	-16.1 (17.8)	2 (2, 3)	-14.6 (26.3)
Florida	2478 (2172, 2847)	63.3	24 (21, 27)	33.3	11 (9, 12)	18.9	5 (5, 6)	22.1 (56.3)	3 (2, 4)	19.3 (28.3)	1 (1, 1)	11.9 (5.7)	2 (1, 2)	12.2 (9.8)
Georgia	1535 (1313, 1777)	57.5	29 (25, 34)	29.4	20 (17, 23)	8.1	10 (8, 12)	8.5 (51.6)	6 (5, 7)	12.4 (42.2)	2 (1, 2)	1.9 (2.1)	3 (2, 4)	2.2 (4.2)
Hawaii	158 (140, 177)	90.6	22 (20, 25)	66.4	10 (9, 12)	22.4	5 (4, 6)	24.9 (53.8)	3 (2, 4)	24.4 (30.1)	1 (1, 1)	15 (6)	1 (1, 2)	15.2 (10.2)

© Bowe B et al. *JAMA Network Open*

Location	Death number		Death per 100,000 persons		Age-standardized Death per 100,000 persons									
					All causes		Due to diabetes		Due to hypertension		Due to glomerulonephritis		Due to other causes	
	2016 (UI)	Percent change from 2002	2016 (UI)	Percent change from 2002	2016 (UI)	Percent change from 2002	2016 (UI)	Percent change from 2002 (PA*)	2016 (UI)	Percent change from 2002 (PA*)	2016 (UI)	Percent change from 2002 (PA*)	2016 (UI)	Percent change from 2002 (PA*)
Idaho	169 (145, 196)	98.2	20 (17, 23)	58.3	12 (10, 14)	36.7	6 (5, 7)	38.1 (48.5)	4 (3, 4)	39.9 (32.4)	1 (1, 1)	29.3 (6.9)	2 (1, 2)	30 (12.2)
Illinois	1808 (1606, 2027)	29.5	28 (25, 31)	26.7	15 (14, 17)	9.7	7 (6, 9)	9.9 (49.5)	4 (4, 5)	14.8 (42.7)	1 (1, 2)	2.7 (2.4)	2 (2, 3)	3.5 (5.4)
Indiana	1118 (955, 1299)	51.3	33 (28, 39)	40.8	18 (16, 22)	26	9 (7, 11)	27.6 (50.4)	5 (4, 7)	28.6 (31.7)	2 (1, 2)	20 (6.7)	3 (2, 3)	19.5 (11.2)
Iowa	373 (329, 426)	55.1	24 (21, 27)	46.6	11 (9, 12)	41.3	5 (4, 6)	42.8 (48.7)	3 (3, 4)	45.5 (32.4)	1 (1, 1)	32.2 (6.8)	2 (1, 2)	33.5 (12.2)
Kansas	413 (354, 481)	30.9	28 (24, 33)	23.1	15 (13, 17)	18.4	7 (6, 9)	21.4 (54.9)	4 (3, 5)	18.1 (28.6)	1 (1, 2)	13.5 (6.3)	2 (2, 3)	12.6 (10.2)
Kentucky	761 (679, 847)	50.8	34 (30, 38)	40	19 (17, 21)	25.1	9 (8, 11)	28 (53.1)	6 (4, 7)	25.4 (28.9)	2 (1, 2)	19.7 (6.9)	3 (2, 3)	18.8 (11.2)
Louisiana	935 (834, 1038)	35	39 (35, 43)	30.8	25 (22, 27)	15.3	12 (11, 14)	16.4 (52.8)	7 (5, 8)	18.6 (32.4)	2 (2, 3)	9.4 (5.6)	4 (3, 4)	9.3 (9.2)

© Bowe B et al. *JAMA Network Open*

Location	Death number		Death per 100,000 persons		Age-standardized Death per 100,000 persons									
					All causes		Due to diabetes		Due to hypertension		Due to glomerulonephritis		Due to other causes	
	2016 (UI)	Percent change from 2002	2016 (UI)	Percent change from 2002	2016 (UI)	Percent change from 2002	2016 (UI)	Percent change from 2002 (PA*)	2016 (UI)	Percent change from 2002 (PA*)	2016 (UI)	Percent change from 2002 (PA*)	2016 (UI)	Percent change from 2002 (PA*)
Maine	201 (180, 223)	40	30 (27, 33)	36.9	13 (12, 14)	13.9	6 (5, 7)	14.3 (48.5)	4 (3, 5)	17.7 (37.7)	1 (1, 1)	7.9 (4.9)	2 (1, 2)	8.3 (9)
Maryland	696 (622, 781)	45.2	22 (20, 25)	31.3	13 (12, 15)	7.7	6 (6, 7)	8.2 (51.9)	4 (3, 4)	12.2 (42.7)	1 (1, 1)	1.4 (1.6)	2 (1, 2)	2 (3.8)
Massachusetts	858 (755, 967)	21.2	24 (21, 28)	14.3	12 (10, 13)	3	6 (5, 7)	2.9 (35.3)	4 (3, 4)	7 (53.2)	1 (1, 1)	-2.1 (4.6)	2 (1, 2)	-1.8 (6.9)
Michigan	1444 (1286, 1603)	40.1	29 (26, 32)	41.6	15 (13, 16)	15.2	7 (6, 8)	16.4 (51.8)	4 (3, 5)	17.8 (33.2)	1 (1, 2)	9.7 (5.6)	2 (2, 3)	9.5 (9.4)
Minnesota	684 (607, 775)	52.3	25 (22, 28)	38.9	12 (11, 14)	22.5	6 (5, 7)	23.6 (48)	4 (3, 5)	25.8 (35.3)	1 (1, 1)	15.7 (5.9)	2 (1, 2)	16.3 (10.8)
Mississippi	624 (527, 719)	41.8	41 (34, 47)	35.8	24 (20, 28)	21.5	12 (10, 14)	24.2 (55)	7 (5, 8)	22.9 (28.4)	2 (2, 3)	14.6 (6.3)	3 (3, 4)	14.6 (10.3)
Missouri	944 (847, 1047)	42.7	30 (27, 34)	33.9	16 (14, 18)	22.2	8 (7, 9)	24.9 (53.4)	5 (4, 6)	23 (29.7)	1 (1, 2)	16 (6.3)	2 (2, 3)	15.7 (10.6)

© Bowe B et al. *JAMA Network Open*

Location	Death number		Death per 100,000 persons		Age-standardized Death per 100,000 persons									
					All causes		Due to diabetes		Due to hypertension		Due to glomerulonephritis		Due to other causes	
	2016 (UI)	Percent change from 2002	2016 (UI)	Percent change from 2002	2016 (UI)	Percent change from 2002	2016 (UI)	Percent change from 2002 (PA*)	2016 (UI)	Percent change from 2002 (PA*)	2016 (UI)	Percent change from 2002 (PA*)	2016 (UI)	Percent change from 2002 (PA*)
Montana	106 (91, 122)	49.8	20 (18, 24)	32.6	10 (9, 12)	16.8	5 (4, 6)	18.7 (52)	3 (2, 4)	17.9 (31.4)	1 (1, 1)	12.2 (6.4)	1 (1, 2)	11.4 (10.2)
Nebraska	256 (227, 288)	44.8	27 (24, 30)	32.8	14 (12, 15)	26.7	6 (5, 8)	27.7 (48.9)	4 (3, 5)	30.9 (33.6)	1 (1, 1)	19 (6.2)	2 (1, 2)	20 (11.2)
Nevada	311 (273, 350)	68.3	21 (19, 24)	22.8	14 (12, 16)	-2.1	7 (6, 8)	-0.6 (12.7)	4 (3, 5)	-0.7 (9.2)	1 (1, 1)	-6.7 (28.1)	2 (2, 3)	-7 (50)
New Hampshire	146 (130, 164)	56.8	22 (19, 24)	48.9	10 (9, 12)	14.5	5 (4, 6)	14.8 (47.9)	3 (3, 4)	17.3 (35.5)	1 (1, 1)	11 (6.5)	1 (1, 2)	9.8 (10.1)
New Jersey	1099 (964, 1238)	21	24 (21, 27)	15.9	12 (11, 14)	0.4	6 (5, 7)	1 (16.6)	4 (3, 4)	3.9 (40.4)	1 (1, 1)	-5.4 (17)	2 (1, 2)	-4.8 (25.9)
New Mexico	248 (210, 291)	90.6	24 (20, 28)	72	14 (11, 16)	36	7 (5, 8)	39.1 (52.3)	4 (3, 5)	35.9 (27.8)	1 (1, 1)	30.6 (7.6)	2 (1, 2)	29.3 (12.3)
New York	1991 (1687, 2294)	27.9	20 (17, 23)	24.4	10 (9, 12)	7	5 (4, 6)	6.6 (45.6)	3 (2, 4)	13 (50.7)	1 (1, 1)	0.5 (0.6)	1 (1, 2)	1.5 (3.1)

© Bowe B et al. *JAMA Network Open*

Location	Death number		Death per 100,000 persons		Age-standardized Death per 100,000 persons									
					All causes		Due to diabetes		Due to hypertension		Due to glomerulonephritis		Due to other causes	
	2016 (UI)	Percent change from 2002	2016 (UI)	Percent change from 2002	2016 (UI)	Percent change from 2002	2016 (UI)	Percent change from 2002 (PA*)	2016 (UI)	Percent change from 2002 (PA*)	2016 (UI)	Percent change from 2002 (PA*)	2016 (UI)	Percent change from 2002 (PA*)
North Carolina	1573 (1403, 1745)	62.9	30 (27, 34)	33.6	18 (16, 19)	15.2	9 (7, 10)	17.5 (55.6)	5 (4, 6)	16.2 (29.7)	1 (1, 2)	9.7 (5.6)	2 (2, 3)	9.2 (9.1)
North Dakota	86 (75, 99)	39.4	23 (20, 26)	18.7	11 (10, 13)	22.6	5 (4, 6)	25.2 (51.9)	3 (3, 4)	22.7 (30)	1 (1, 1)	17.3 (6.7)	2 (1, 2)	17.2 (11.4)
Ohio	1848 (1675, 2047)	37.9	31 (28, 35)	36.4	16 (15, 18)	18.5	8 (7, 9)	19.9 (52)	5 (4, 6)	20.9 (31.9)	1 (1, 2)	12.9 (6.1)	2 (2, 3)	12.3 (10)
Oklahoma	562 (503, 628)	54.9	28 (25, 32)	38.8	17 (15, 19)	35.5	8 (7, 10)	39.7 (53.4)	5 (4, 6)	34.3 (27.3)	1 (1, 2)	29 (7.3)	2 (2, 3)	28.1 (11.9)
Oregon	432 (384, 477)	63.8	21 (19, 23)	41.9	11 (10, 12)	22.2	5 (4, 6)	22.6 (48)	3 (3, 4)	27 (35.6)	1 (1, 1)	15 (5.8)	2 (1, 2)	15.8 (10.6)
Pennsylvania	2130 (1907, 2356)	27.5	33 (29, 36)	24.2	15 (13, 16)	11.7	7 (6, 8)	12.1 (49.6)	4 (4, 5)	15.8 (38.4)	1 (1, 2)	5.6 (4.2)	2 (2, 3)	6.1 (7.8)
Rhode Island	122 (105, 140)	37.6	22 (19, 26)	39.4	10 (9, 12)	24.8	5 (4, 6)	24.6 (47.8)	3 (2, 4)	31.2 (35.4)	1 (1, 1)	16.6 (5.9)	1 (1, 2)	18.1 (10.9)

© Bowe B et al. *JAMA Network Open*

Location	Death number		Death per 100,000 persons		Age-standardized Death per 100,000 persons									
					All causes		Due to diabetes		Due to hypertension		Due to glomerulonephritis		Due to other causes	
	2016 (UI)	Percent change from 2002	2016 (UI)	Percent change from 2002	2016 (UI)	Percent change from 2002	2016 (UI)	Percent change from 2002 (PA*)	2016 (UI)	Percent change from 2002 (PA*)	2016 (UI)	Percent change from 2002 (PA*)	2016 (UI)	Percent change from 2002 (PA*)
South Carolina	865 (736, 1014)	69.4	34 (29, 40)	40.8	19 (16, 23)	17.3	10 (8, 12)	17.6 (49.7)	6 (4, 7)	22.9 (35.9)	2 (1, 2)	10.1 (5.2)	3 (2, 4)	10.6 (9.2)
South Dakota	100 (87, 114)	52.9	23 (20, 27)	36.2	11 (9, 13)	28.3	5 (4, 6)	30.6 (51.2)	3 (3, 4)	29.7 (29.9)	1 (1, 1)	22 (7)	2 (1, 2)	22 (11.8)
Tennessee	1002 (893, 1112)	72	30 (26, 33)	50.6	17 (15, 19)	33.4	8 (7, 10)	35.4 (51.1)	5 (4, 6)	37.2 (30.8)	1 (1, 2)	24.5 (6.6)	2 (2, 3)	25.4 (11.5)
Texas	3288 (2931, 3718)	92.7	23 (21, 27)	50.5	17 (15, 19)	33.4	8 (7, 10)	35.8 (52)	5 (4, 6)	36 (29.8)	1 (1, 2)	25 (6.6)	2 (2, 3)	25.6 (11.5)
Utah	236 (210, 265)	89.9	16 (14, 18)	47	13 (12, 15)	31.1	6 (5, 7)	32.1 (48.3)	4 (3, 5)	35.1 (33.4)	1 (1, 1)	23.9 (6.6)	2 (1, 2)	24.5 (11.7)
Vermont	68 (60, 76)	45.8	22 (19, 24)	43.6	10 (9, 11)	12.6	5 (4, 5)	11.5 (43)	3 (2, 4)	18.3 (42.4)	1 (1, 1)	7.8 (5.4)	1 (1, 2)	7.8 (9.2)
Virginia	1171 (1046, 1306)	57.8	27 (24, 31)	37	16 (14, 18)	14.9	8 (7, 9)	15.2 (49.1)	5 (4, 6)	20.1 (37.7)	1 (1, 2)	7.6 (4.5)	2 (2, 3)	8.6 (8.6)

© Bowe B et al. *JAMA Network Open*

Location	Death number		Death per 100,000 persons		Age-standardized Death per 100,000 persons									
					All causes		Due to diabetes		Due to hypertension		Due to glomerulonephritis		Due to other causes	
	2016 (UI)	Percent change from 2002	2016 (UI)	Percent change from 2002	2016 (UI)	Percent change from 2002	2016 (UI)	Percent change from 2002 (PA*)	2016 (UI)	Percent change from 2002 (PA*)	2016 (UI)	Percent change from 2002 (PA*)	2016 (UI)	Percent change from 2002 (PA*)
Washington	647 (581, 718)	92.1	18 (16, 20)	61.6	10 (9, 11)	36.1	5 (4, 6)	36.5 (47.8)	3 (3, 4)	41.7 (33.8)	1 (1, 1)	27.7 (6.6)	1 (1, 2)	28.6 (11.8)
West Virginia	380 (339, 425)	38.6	41 (37, 46)	38	20 (18, 23)	27.8	10 (8, 11)	30.4 (52.3)	6 (5, 7)	28.8 (29.1)	2 (1, 2)	22.3 (7.1)	3 (2, 4)	21.3 (11.5)
Wisconsin	833 (748, 926)	58.2	29 (26, 32)	49.6	14 (12, 15)	30.7	7 (6, 8)	31.4 (47.8)	4 (3, 5)	35.1 (33.8)	1 (1, 1)	23.6 (6.6)	2 (2, 2)	24.3 (11.8)
Wyoming	48 (41, 55)	48.1	17 (14, 19)	28.2	10 (9, 12)	14.6	5 (4, 6)	15.7 (51.2)	3 (2, 4)	17.6 (34.1)	1 (1, 1)	9.2 (5.6)	1 (1, 2)	8.8 (9.1)

UI is the uncertainty interval, it reflects the certainty of an estimate based on data availability, studies size and consistency across data sources
*Percentage attributed to overall rate is the proportion of overall age-standardized rate change attributed by each of the following four causes: diabetes, hypertension, glomerulonephritis and other causes

© Bowe B et al. *JAMA Network Open*

eTable 7b: Number and age-standardized rate for overall Death due to CKD and by cause in males, with percentage change from 2002 to 2016 for the Unites States and by state and district.

Location	Death number		Death per 100,000 persons		Age-standardized Death per 100,000 persons									
					All causes		Due to diabetes		Due to hypertension		Due to glomerulonephritis		Due to other causes	
	2016 (UI)	Percent change from 2002	2016 (UI)	Percent change from 2002	2016 (UI)	Percent change from 2002	2016 (UI)	Percent change from 2002 (PA*)	2016 (UI)	Percent change from 2002 (PA*)	2016 (UI)	Percent change from 2002 (PA*)	2016 (UI)	Percent change from 2002 (PA*)
United States	40933 (39545, 42288)	65.9	26 (25, 27)	47.4	20 (19, 20)	13.3	10 (9, 11)	16.3 (62.4)	6 (5, 7)	12.1 (26.1)	1 (1, 2)	8.6 (4.4)	3 (2, 3)	6.7 (7.1)
Alabama	814 (699, 936)	48.6	35 (30, 40)	36.9	27 (23, 31)	6.2	14 (12, 17)	10.9 (89.2)	7 (6, 9)	1.1 (4.8)	2 (1, 2)	4.1 (4.6)	4 (3, 5)	0.6 (1.5)
Alaska	44 (38, 52)	123.1	11 (10, 13)	92.6	14 (12, 16)	29.7	7 (6, 9)	29.8 (51)	4 (3, 5)	35.6 (33.5)	1 (1, 1)	21.3 (5)	2 (1, 2)	21.7 (10.4)
Arizona	744 (651, 837)	85	22 (19, 24)	45.3	15 (13, 17)	5.7	8 (7, 9)	10.9 (94.8)	4 (3, 5)	0.3 (1.4)	1 (1, 1)	2 (2.4)	2 (2, 3)	-0.6 (1.3)
Arkansas	477 (419, 532)	65	33 (29, 36)	49.2	24 (21, 27)	21.2	13 (11, 15)	25.9 (62.5)	6 (5, 8)	17.5 (22.9)	2 (1, 2)	16.2 (5.4)	3 (3, 4)	13.8 (9.3)
California	3771 (3248, 4310)	97.5	19 (17, 22)	75.5	16 (14, 18)	29.2	8 (7, 10)	32.3 (56.5)	4 (4, 5)	29.5 (28.4)	1 (1, 1)	21.6 (5.1)	2 (2, 3)	20.7 (10)

© Bowe B et al. *JAMA Network Open*

Location	Death number		Death per 100,000 persons		Age-standardized Death per 100,000 persons									
					All causes		Due to diabetes		Due to hypertension		Due to glomerulonephritis		Due to other causes	
	2016 (UI)	Percent change from 2002	2016 (UI)	Percent change from 2002	2016 (UI)	Percent change from 2002	2016 (UI)	Percent change from 2002 (PA*)	2016 (UI)	Percent change from 2002 (PA*)	2016 (UI)	Percent change from 2002 (PA*)	2016 (UI)	Percent change from 2002 (PA*)
Colorado	451 (403, 507)	88.9	16 (15, 18)	54.3	15 (13, 16)	7.6	7 (6, 9)	10.6 (68.4)	4 (4, 5)	6.3 (25.2)	1 (1, 1)	3.7 (3.2)	2 (1, 2)	1.8 (3.3)
Connecticut	428 (371, 496)	41	25 (21, 28)	35.1	17 (15, 19)	6.2	8 (7, 10)	7 (56.7)	5 (4, 6)	8.4 (39.9)	1 (1, 1)	1.9 (2)	2 (2, 3)	0.6 (1.4)
Delaware	130 (117, 145)	77.3	28 (25, 31)	50.4	20 (18, 22)	8.3	10 (9, 12)	11 (66.9)	6 (5, 7)	7.6 (25.8)	1 (1, 2)	4.5 (3.8)	3 (2, 3)	2.1 (3.5)
District of Columbia	80 (69, 91)	9.2	24 (21, 28)	-8.2	24 (21, 28)	-11	13 (11, 16)	-8.6 (41.7)	6 (5, 8)	-8.8 (19.8)	2 (1, 2)	-20.8 (14.2)	3 (3, 4)	-18.6 (24.4)
Florida	2924 (2583, 3314)	77.9	29 (26, 33)	45	17 (15, 19)	17.6	9 (8, 10)	22.9 (66.1)	5 (4, 5)	12.8 (20.6)	1 (1, 1)	12.7 (5)	2 (2, 3)	10.3 (8.3)
Georgia	1391 (1206, 1611)	85.2	28 (24, 32)	54.9	26 (23, 30)	9.6	14 (12, 17)	11.9 (63.9)	7 (6, 9)	9.6 (27.3)	2 (1, 2)	5.5 (3.9)	3 (3, 4)	3.4 (4.9)
Hawaii	175 (157, 194)	80.5	24 (22, 27)	54.9	16 (14, 18)	20.9	8 (7, 10)	24.8 (60.3)	4 (4, 5)	18.4 (25.1)	1 (1, 1)	16.2 (5.3)	2 (2, 3)	13.9 (9.3)

© Bowe B et al. *JAMA Network Open*

Location	Death number		Death per 100,000 persons		Age-standardized Death per 100,000 persons									
					All causes		Due to diabetes		Due to hypertension		Due to glomerulonephritis		Due to other causes	
	2016 (UI)	Percent change from 2002	2016 (UI)	Percent change from 2002	2016 (UI)	Percent change from 2002	2016 (UI)	Percent change from 2002 (PA*)	2016 (UI)	Percent change from 2002 (PA*)	2016 (UI)	Percent change from 2002 (PA*)	2016 (UI)	Percent change from 2002 (PA*)
Idaho	171 (149, 198)	113	20 (18, 24)	70	16 (14, 18)	28.7	8 (7, 9)	31.6 (54.3)	5 (4, 6)	28.5 (29.8)	1 (1, 1)	23.1 (5.4)	2 (2, 3)	21.5 (10.5)
Illinois	1801 (1609, 1997)	45.9	29 (26, 32)	42.3	23 (20, 25)	8.2	12 (10, 13)	10 (62)	7 (5, 8)	9.6 (33.3)	1 (1, 2)	2.3 (1.9)	3 (2, 4)	1.6 (2.8)
Indiana	1031 (888, 1180)	62.3	32 (27, 36)	50.1	25 (22, 29)	16.4	13 (11, 15)	19.1 (58.2)	7 (6, 9)	15.7 (28.1)	2 (1, 2)	12.5 (5.1)	3 (3, 4)	10.1 (8.6)
Iowa	350 (311, 396)	72.5	23 (20, 25)	59.6	15 (14, 17)	34	8 (7, 9)	37.3 (54)	5 (4, 6)	33.1 (29.6)	1 (1, 1)	28.4 (5.5)	2 (2, 3)	26.6 (10.9)
Kansas	399 (342, 462)	51.7	27 (24, 32)	40.4	21 (18, 24)	14.8	11 (9, 13)	19 (63.4)	6 (5, 7)	11.7 (23.8)	1 (1, 2)	11 (5)	3 (2, 4)	8.4 (7.9)
Kentucky	721 (647, 805)	68.3	33 (30, 37)	54.7	27 (24, 30)	17.9	14 (12, 16)	21.8 (61.3)	8 (6, 9)	14.9 (24.2)	2 (1, 2)	14.5 (5.4)	4 (3, 4)	11.6 (9.1)
Louisiana	864 (775, 957)	49.8	38 (34, 42)	43	32 (29, 36)	12	17 (15, 20)	14.5 (62.6)	9 (7, 10)	12.4 (27.8)	2 (2, 3)	6.2 (3.6)	4 (3, 5)	5 (6)

© Bowe B et al. *JAMA Network Open*

Location	Death number		Death per 100,000 persons		Age-standardized Death per 100,000 persons									
					All causes		Due to diabetes		Due to hypertension		Due to glomerulonephritis		Due to other causes	
	2016 (UI)	Percent change from 2002	2016 (UI)	Percent change from 2002	2016 (UI)	Percent change from 2002	2016 (UI)	Percent change from 2002 (PA*)	2016 (UI)	Percent change from 2002 (PA*)	2016 (UI)	Percent change from 2002 (PA*)	2016 (UI)	Percent change from 2002 (PA*)
Maine	199 (178, 220)	62	31 (27, 34)	56.8	19 (17, 21)	12.5	9 (8, 11)	14 (55.2)	6 (5, 7)	13.3 (32.2)	1 (1, 2)	9.2 (4.9)	2 (2, 3)	7 (7.8)
Maryland	687 (621, 764)	52.8	24 (21, 26)	37.2	19 (17, 21)	3.5	10 (9, 11)	5.4 (60.2)	5 (4, 6)	4.8 (28.5)	1 (1, 2)	-1.9 (2.9)	3 (2, 3)	-2.7 (8.4)
Massachusetts	911 (814, 1015)	39	27 (25, 31)	29.7	20 (18, 22)	3	10 (8, 12)	3.9 (49.9)	6 (5, 7)	5.1 (39.3)	1 (1, 2)	-1.2 (2)	3 (2, 3)	-2.5 (8.9)
Michigan	1384 (1246, 1534)	53.9	28 (26, 31)	55.1	21 (19, 23)	12.2	11 (9, 12)	14.8 (60.8)	6 (5, 7)	11.6 (27.6)	1 (1, 2)	8.3 (4.6)	3 (2, 3)	6.1 (7)
Minnesota	668 (595, 751)	64.7	24 (22, 27)	49.3	18 (16, 20)	12.6	9 (8, 11)	15.6 (59.9)	6 (5, 7)	11.3 (28.4)	1 (1, 1)	8.3 (4.3)	2 (2, 3)	6.7 (7.4)
Mississippi	555 (473, 636)	56.3	38 (33, 44)	49.2	32 (27, 36)	16	17 (14, 20)	20.3 (66)	8 (7, 10)	12.2 (20.7)	2 (2, 3)	12 (5.3)	4 (3, 5)	9.2 (8.1)
Missouri	906 (812, 1011)	58.9	30 (27, 34)	47.1	22 (20, 25)	15.4	12 (10, 13)	19.4 (63.3)	6 (5, 8)	12.5 (23.7)	1 (1, 2)	11.2 (4.9)	3 (2, 4)	8.8 (8)

© Bowe B et al. *JAMA Network Open*

Location	Death number		Death per 100,000 persons		Age-standardized Death per 100,000 persons									
					All causes		Due to diabetes		Due to hypertension		Due to glomerulonephritis		Due to other causes	
	2016 (UI)	Percent change from 2002	2016 (UI)	Percent change from 2002	2016 (UI)	Percent change from 2002	2016 (UI)	Percent change from 2002 (PA*)	2016 (UI)	Percent change from 2002 (PA*)	2016 (UI)	Percent change from 2002 (PA*)	2016 (UI)	Percent change from 2002 (PA*)
Montana	104 (88, 120)	56.5	20 (17, 23)	36.3	13 (11, 15)	2.9	7 (5, 8)	7.4 (80.9)	4 (3, 5)	-1.6 (11.2)	1 (1, 1)	0.8 (1.2)	2 (1, 2)	-2.1 (6.7)
Nebraska	235 (210, 262)	50.5	25 (22, 27)	35	18 (17, 21)	13.6	9 (8, 11)	16 (58.5)	6 (4, 7)	13.5 (29.6)	1 (1, 2)	9.3 (4.5)	2 (2, 3)	7.2 (7.4)
Nevada	390 (346, 433)	73.6	27 (24, 29)	30.1	21 (19, 24)	-6.5	11 (10, 13)	-3.4 (26.9)	6 (5, 7)	-8.8 (38.4)	1 (1, 2)	-9.1 (9.5)	3 (2, 4)	-11.6 (25.1)
New Hampshire	149 (133, 167)	87.1	23 (20, 25)	76.6	16 (14, 17)	17.3	8 (7, 9)	19.7 (55.8)	5 (4, 6)	16.3 (28.7)	1 (1, 1)	15.3 (5.8)	2 (2, 3)	12.1 (9.7)
New Jersey	1174 (1048, 1317)	36.1	27 (24, 30)	28.9	20 (18, 23)	1.8	10 (9, 12)	2.7 (42.9)	6 (5, 7)	3.9 (35.2)	1 (1, 2)	-2.5 (5.4)	3 (2, 3)	-3.7 (16.5)
New Mexico	262 (225, 301)	94.4	25 (22, 29)	72.9	19 (16, 21)	26.2	10 (8, 12)	30.1 (58.5)	5 (4, 6)	24.2 (25.8)	1 (1, 2)	20.8 (5.6)	2 (2, 3)	18.6 (10.1)
New York	2016 (1760, 2306)	38.9	21 (18, 24)	33.4	16 (14, 18)	4.7	8 (7, 10)	5.8 (57.8)	4 (4, 5)	7 (37.8)	1 (1, 1)	-0.5 (0.7)	2 (2, 3)	-1.4 (3.8)

© Bowe B et al. *JAMA Network Open*

Location	Death number		Death per 100,000 persons		Age-standardized Death per 100,000 persons									
					All causes		Due to diabetes		Due to hypertension		Due to glomerulonephritis		Due to other causes	
	2016 (UI)	Percent change from 2002	2016 (UI)	Percent change from 2002	2016 (UI)	Percent change from 2002	2016 (UI)	Percent change from 2002 (PA*)	2016 (UI)	Percent change from 2002 (PA*)	2016 (UI)	Percent change from 2002 (PA*)	2016 (UI)	Percent change from 2002 (PA*)
North Carolina	1437 (1298, 1582)	81.7	29 (26, 32)	50.7	23 (21, 26)	9.7	12 (11, 14)	12.7 (67.5)	6 (5, 8)	8.1 (23.4)	2 (1, 2)	5.7 (4)	3 (2, 4)	3.4 (5)
North Dakota	87 (76, 99)	55.1	22 (19, 25)	25.1	17 (15, 19)	22.2	8 (7, 10)	24.1 (53.9)	5 (4, 6)	22.5 (30.2)	1 (1, 1)	19.1 (5.7)	2 (2, 3)	16.1 (10.2)
Ohio	1718 (1545, 1908)	50.1	30 (27, 34)	46.4	22 (20, 25)	11.6	11 (10, 13)	15.2 (65.9)	6 (5, 8)	8.9 (22.2)	1 (1, 2)	8.7 (5)	3 (2, 4)	5.7 (6.9)
Oklahoma	521 (467, 582)	69	27 (24, 30)	50	22 (19, 24)	24.7	11 (10, 13)	29.9 (61.3)	6 (5, 7)	20 (22.9)	1 (1, 2)	20.6 (5.7)	3 (2, 4)	17.4 (10)
Oregon	433 (392, 482)	82.3	22 (20, 24)	58.5	15 (14, 17)	20.8	8 (6, 9)	23.9 (56.9)	4 (4, 5)	20 (28.8)	1 (1, 1)	15.6 (4.9)	2 (2, 2)	14 (9.4)
Pennsylvania	2039 (1842, 2265)	34	33 (29, 36)	27.8	22 (19, 24)	4	11 (10, 13)	6 (65.5)	6 (5, 8)	4.4 (27.5)	1 (1, 2)	-0.6 (0.9)	3 (2, 4)	-2 (6.1)
Rhode Island	120 (105, 138)	36.6	24 (21, 27)	36.6	16 (14, 19)	9.8	8 (7, 10)	11 (56.4)	5 (4, 6)	11.7 (35.2)	1 (1, 1)	4.8 (3.3)	2 (2, 3)	3.5 (5.1)

© Bowe B et al. *JAMA Network Open*

Location	Death number		Death per 100,000 persons		Age-standardized Death per 100,000 persons									
					All causes		Due to diabetes		Due to hypertension		Due to glomerulonephritis		Due to other causes	
	2016 (UI)	Percent change from 2002	2016 (UI)	Percent change from 2002	2016 (UI)	Percent change from 2002	2016 (UI)	Percent change from 2002 (PA*)	2016 (UI)	Percent change from 2002 (PA*)	2016 (UI)	Percent change from 2002 (PA*)	2016 (UI)	Percent change from 2002 (PA*)
South Carolina	765 (666, 879)	77.4	32 (28, 37)	47.6	24 (21, 28)	6.2	13 (11, 15)	8.3 (69.4)	7 (5, 8)	6.6 (28.8)	2 (1, 2)	1.4 (1.6)	3 (2, 4)	-0.1 (0.2)
South Dakota	92 (80, 105)	49.7	21 (18, 24)	30	15 (13, 17)	9.7	7 (6, 9)	13.4 (67.8)	4 (3, 5)	7 (21.4)	1 (1, 1)	6.8 (4.7)	2 (2, 3)	4.2 (6.1)
Tennessee	905 (806, 999)	88.3	28 (25, 31)	64.5	22 (20, 24)	24.6	12 (10, 13)	28.3 (58.6)	6 (5, 7)	23.3 (26.4)	1 (1, 2)	18.8 (5.3)	3 (2, 4)	16.9 (9.7)
Texas	3161 (2815, 3527)	112.3	23 (20, 25)	65.8	22 (20, 25)	26.9	12 (10, 14)	32.1 (60.6)	6 (5, 7)	22.5 (23.9)	1 (1, 2)	22.1 (5.5)	3 (2, 4)	19.3 (10)
Utah	243 (217, 270)	99.3	16 (14, 18)	53.8	17 (15, 19)	20	9 (7, 10)	23.2 (57.1)	5 (4, 6)	19 (28.7)	1 (1, 1)	14.8 (4.9)	2 (2, 3)	13.3 (9.3)
Vermont	63 (56, 71)	70.5	21 (18, 23)	67.2	14 (12, 15)	16.4	7 (6, 8)	17.2 (52)	4 (3, 5)	18.3 (33.6)	1 (1, 1)	12.5 (5.1)	2 (1, 2)	10.9 (9.3)
Virginia	1102 (992, 1223)	66.9	27 (24, 29)	43.8	22 (20, 24)	5.6	11 (10, 13)	7.7 (67.6)	6 (5, 8)	6.1 (30.1)	1 (1, 2)	0.8 (1)	3 (2, 4)	-0.5 (1.3)

© Bowe B et al. *JAMA Network Open*

Location	Death number		Death per 100,000 persons		Age-standardized Death per 100,000 persons									
					All causes		Due to diabetes		Due to hypertension		Due to glomerulonephritis		Due to other causes	
	2016 (UI)	Percent change from 2002	2016 (UI)	Percent change from 2002	2016 (UI)	Percent change from 2002	2016 (UI)	Percent change from 2002 (PA*)	2016 (UI)	Percent change from 2002 (PA*)	2016 (UI)	Percent change from 2002 (PA*)	2016 (UI)	Percent change from 2002 (PA*)
Washington	634 (567, 706)	113.9	18 (16, 20)	78.3	14 (12, 15)	34.8	7 (6, 8)	38.4 (54.4)	4 (3, 5)	33.8 (29.4)	1 (1, 1)	28.3 (5.4)	2 (1, 2)	27.1 (10.8)
West Virginia	374 (335, 418)	66.2	41 (37, 46)	62	28 (25, 31)	29.6	15 (13, 17)	33.1 (56.8)	8 (6, 9)	28.3 (27)	2 (1, 2)	24.4 (5.7)	4 (3, 5)	22 (10.5)
Wisconsin	779 (702, 861)	60.4	27 (24, 30)	50.3	19 (17, 21)	14.4	10 (8, 11)	16.9 (57.4)	6 (5, 7)	14.2 (30.1)	1 (1, 2)	10 (4.6)	3 (2, 3)	8.3 (8)
Wyoming	53 (46, 61)	69	18 (15, 20)	42.6	14 (12, 16)	8.9	7 (6, 9)	13.9 (76)	4 (3, 5)	4.4 (14.9)	1 (1, 1)	5.7 (4.3)	2 (1, 2)	3 (4.8)

UI is the uncertainty interval, it reflects the certainty of an estimate based on data availability, studies size and consistency across data sources
*Percentage attributed to overall rate is the proportion of overall age-standardized rate change attributed by each of the following four causes: diabetes, hypertension, glomerulonephritis and other causes

© Bowe B et al. *JAMA Network Open*

eFigures

eFigure Legends:

eFigure 1: Maps of the Age-standardized disability-adjusted life years (DALYs) due to chronic kidney disease in the United States in females and males. A) 2016 age-standardized DALY rates due to chronic kidney disease in females; B) 2016 age-standardized DALY rates due to chronic kidney disease in males; C) Percentage change in age-standardized DALY rates due to chronic kidney disease from 2002 to 2016 in females; D) Percentage change in age-standardized DALY rates due to chronic kidney disease from 2002 to 2016 in males. Rates are per 100,000 persons. Percentage change maps are colored by deciles of their respective values.

eFigure 2: Maps of the Age-standardized death due to chronic kidney disease in the United States in females and males. A) 2016 death rates due to chronic kidney disease in females; B) 2016 death rates due to chronic kidney disease in males; C) Percentage change in death rates due to chronic kidney disease from 2002 to 2016 in females; D) Percentage change in death rates due to chronic kidney disease from 2002 to 2016 in males. Rates are per 100,000 persons. Percentage change maps are colored by deciles of their respective values.

eFigure 3: Decomposition of the percentage change in chronic kidney disease burden in the United States from 2002 to 2016 due to changes in age structure, size of the population, and risk exposure. A) Disability-adjusted life years (DALYs); B) Death. Geographies are ordered by total percent change.

eFigure 4: Decomposition of the percentage change in age-standardized death rates due to chronic kidney disease in the United States from 2002 to 2016 by risk factor. Geographies are ordered by total percentage change. Risk exposures are colored similarly to others that fall in the same risk exposure category (environmental risk factors, metabolic risk factors, and dietary risk factors). SSB, sugar sweetened beverages.

eFigure 5: Decomposition of the percentage change in age-standardized CKD burden rate in the United States from 2002 to 2016 by cause. A) Disability-adjusted life years (DALYs); B) Death. Geographies are ordered by total percent change.

eFigure 6: Age-standardized disability-adjusted life year (DALY) rates due to chronic kidney disease in 2002 vs. 2016 by socio-demographic index (SDI). SDI is a summary measure of a geographies income, education, and fertility rate. Values range from 0 to 1, where a higher value indicates higher socio-demographic development.

eFigure 7: Observed to expected ratio of age-standardized disability-adjusted life year (DALY) rate due to CKD in 2016 based on a state or district's socio-demographic index (SDI). SDI is a summary measure of a geographies income, education, and fertility rate. Values range from 0 to 1, where a higher value indicates higher socio-demographic development.

© Bowe B et al. *JAMA Network Open*

eFigure 8: Frontier analysis for age-standardized disability-adjusted life year (DALY) rates due to CKD by socio-demographic index (SDI) in 2016. The Y axis is inverted (lower DALY rates are at the top). SDI is a summary measure of a geographies income, education, and fertility rate. Values range from 0 to 1, where a higher value indicates higher socio-demographic development. Distance from the frontier indicates the effective difference; all DALY rates less than the frontier are considered to have a zero effective difference.

© Bowe B et al. *JAMA Network Open*

eFigure 1A: 2016 age-standardized DALY rates due to chronic kidney disease in females

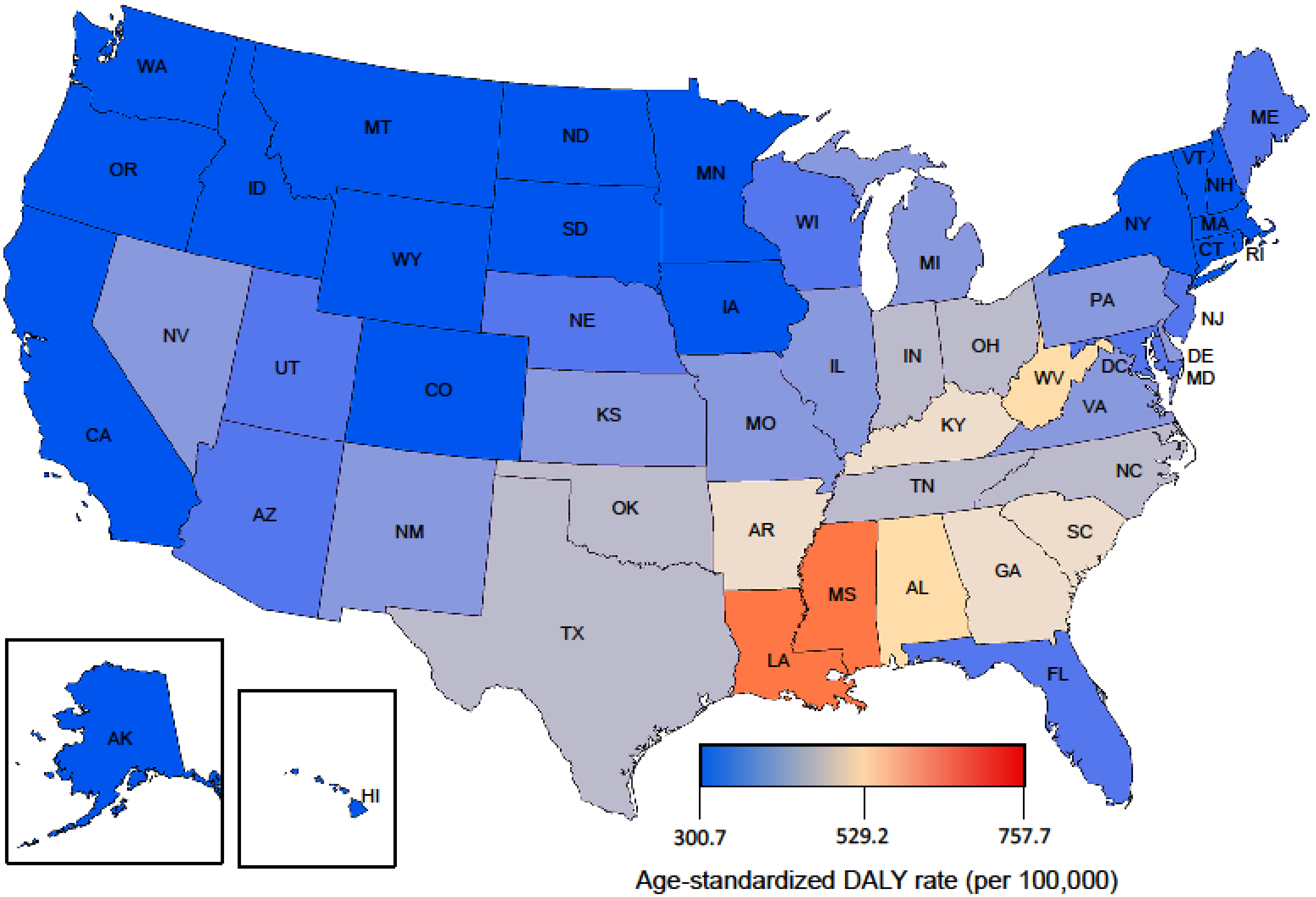

© Bowe B et al. *JAMA Network Open*

eFigure 1B: 2016 age-standardized DALY rates due to chronic kidney disease in males

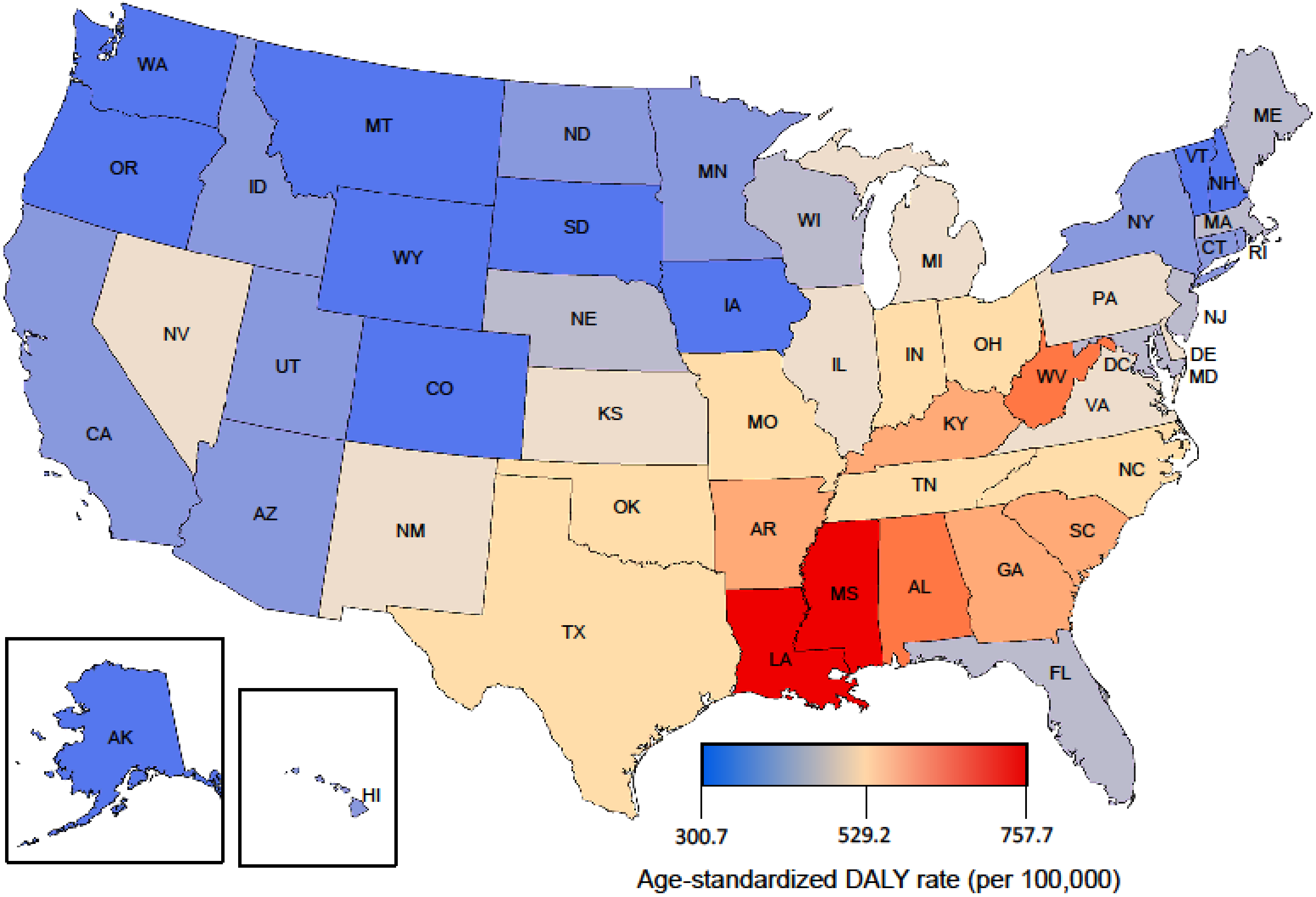

© Bowe B et al. *JAMA Network Open*

eFigure 1C: Percentage change in age-standardized DALY rates due to chronic kidney disease from 2002 to 2016 in females

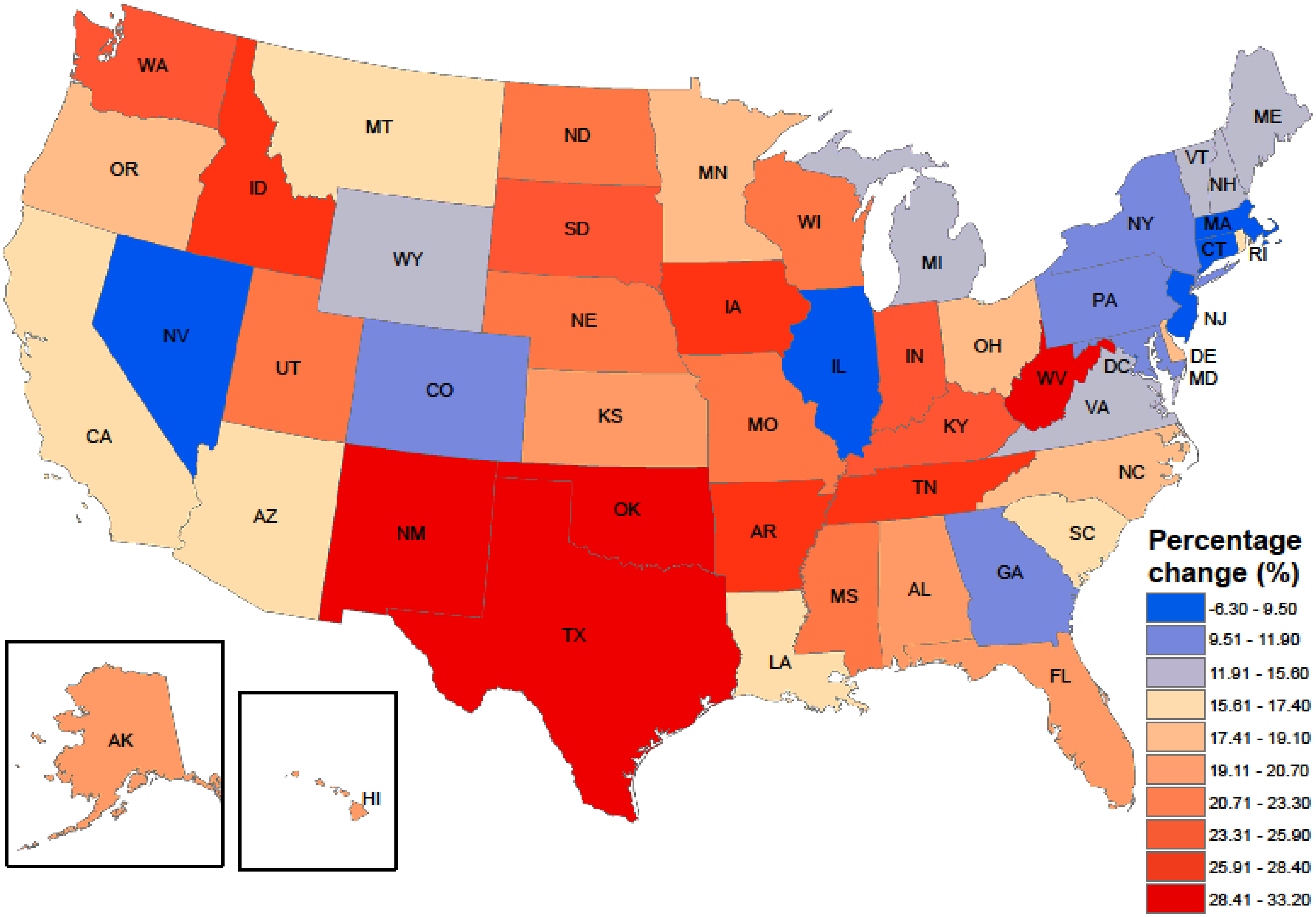

© Bowe B et al. *JAMA Network Open*

eFigure 1D: Percentage change in age-standardized DALY rates due to chronic kidney disease from 2002 to 2016 in males

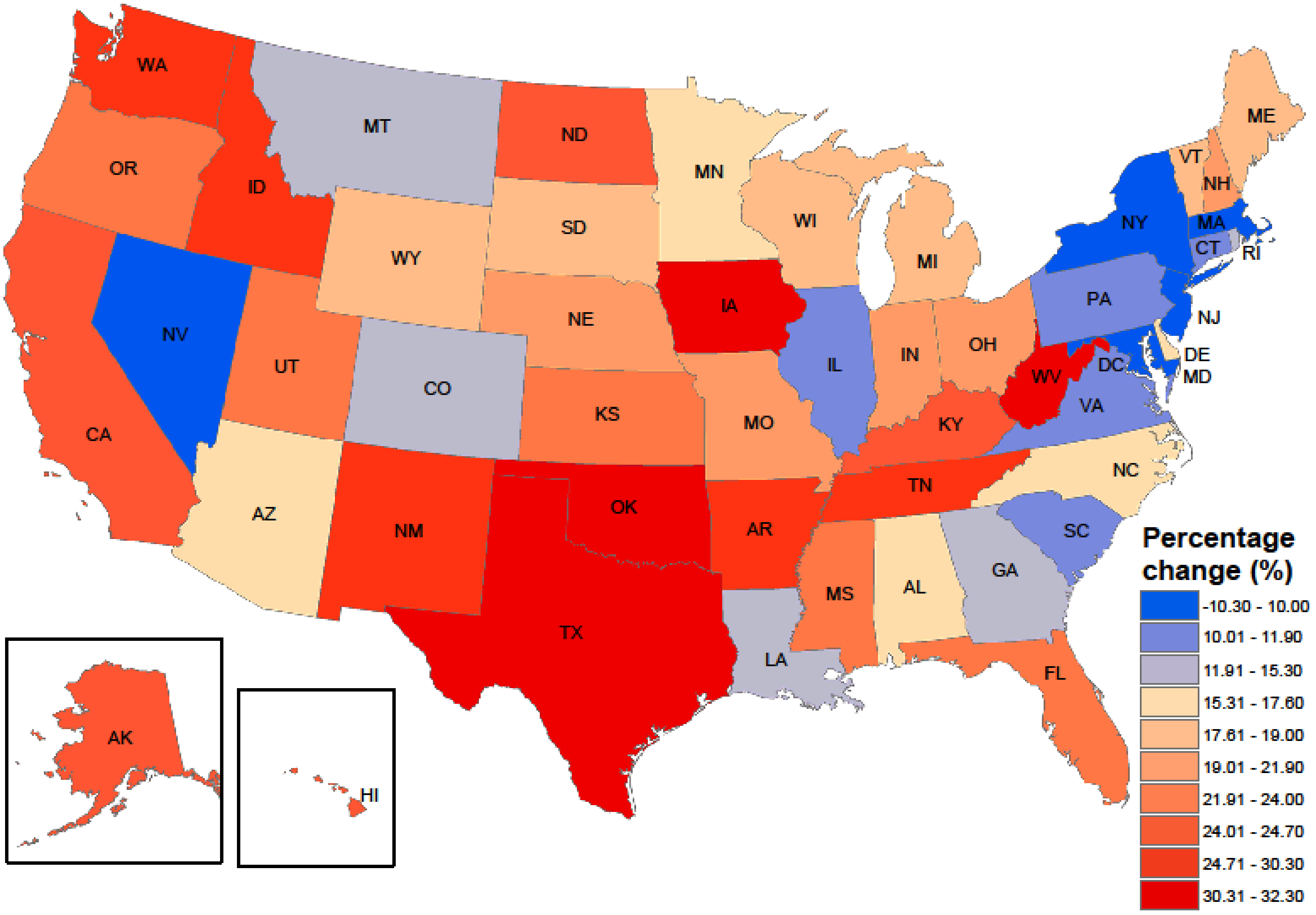

© Bowe B et al. *JAMA Network Open*

eFigure 2A: 2016 age-standardized death rates due to chronic kidney disease in females

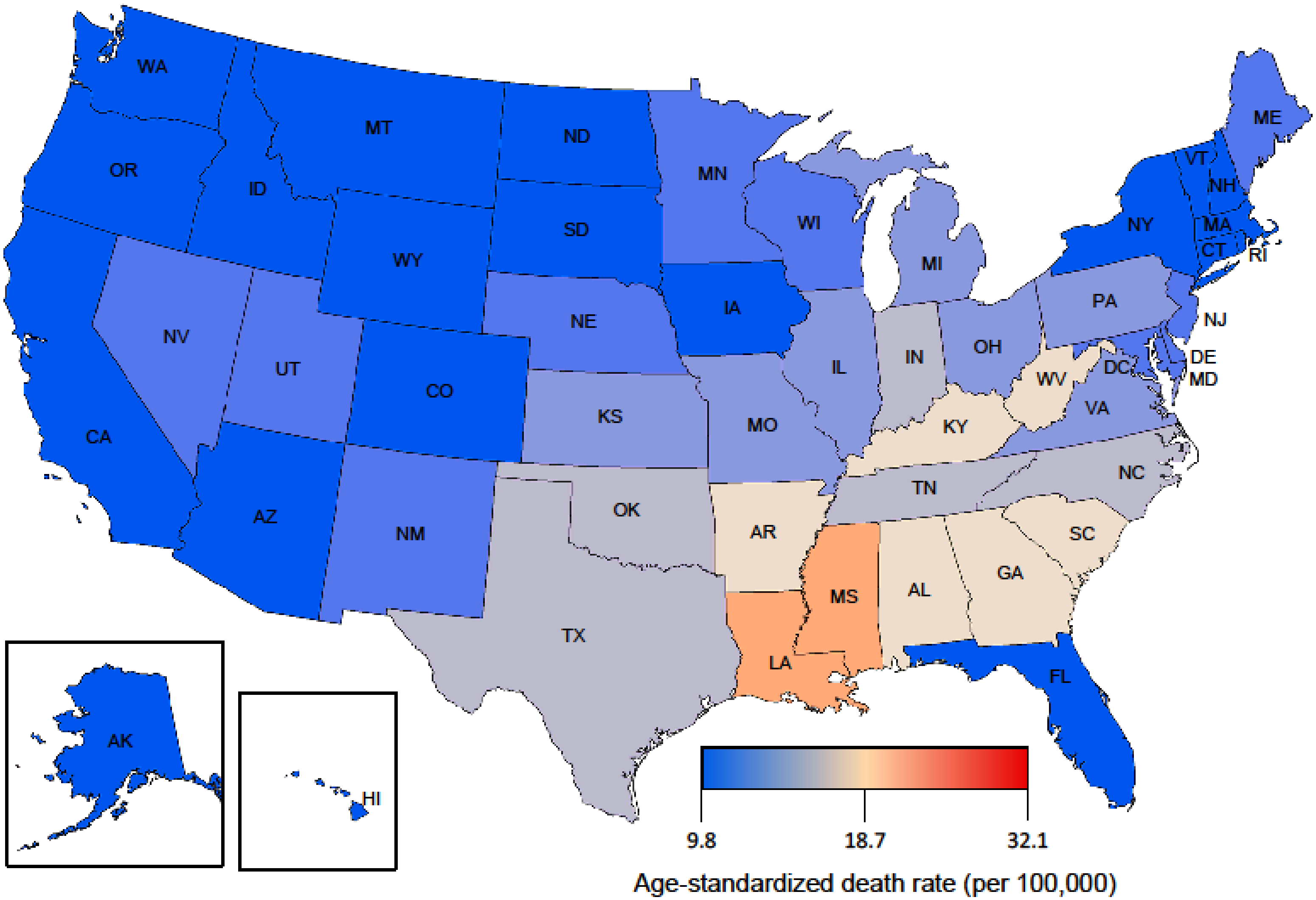

© Bowe B et al. *JAMA Network Open*

eFigure 2B: 2016 age-standardized death rates due to chronic kidney disease in males

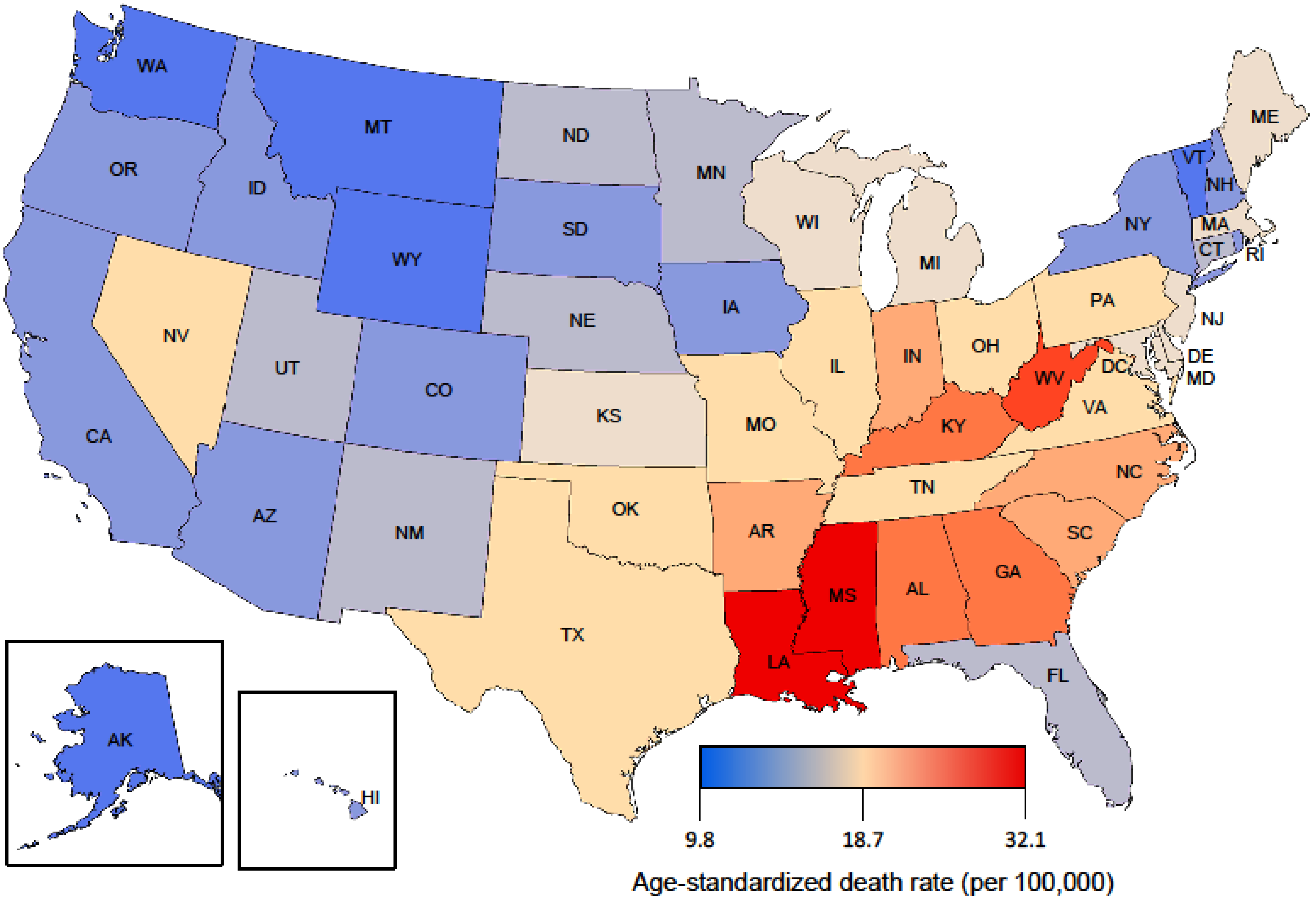

© Bowe B et al. *JAMA Network Open*

eFigure 2C: Percentage change in age-standardized death rates due to chronic kidney disease from 2002 to 2016 in females

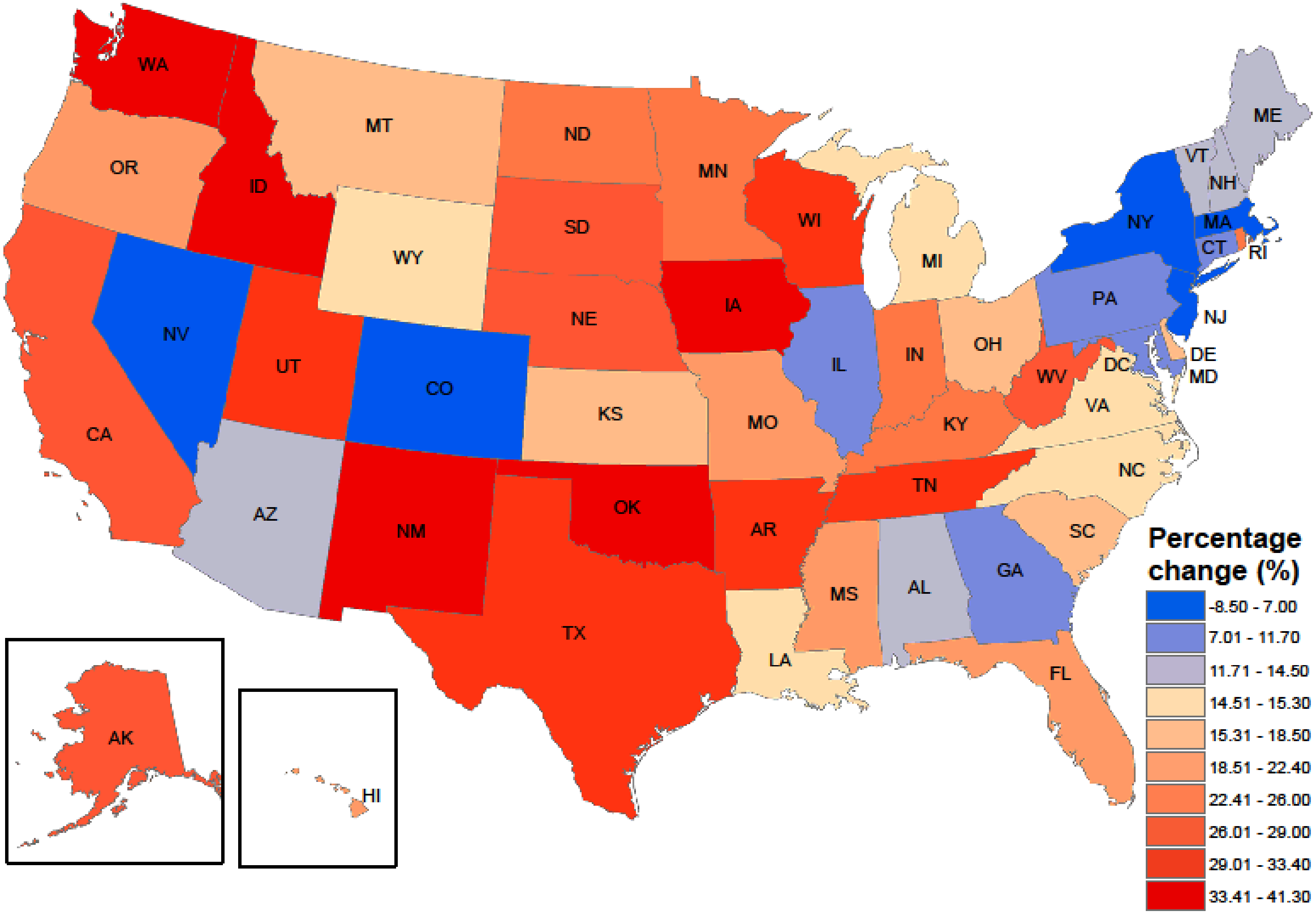

© Bowe B et al. *JAMA Network Open*

eFigure 2D: Percentage change in age-standardized death rates due to chronic kidney disease from 2002 to 2016 in males

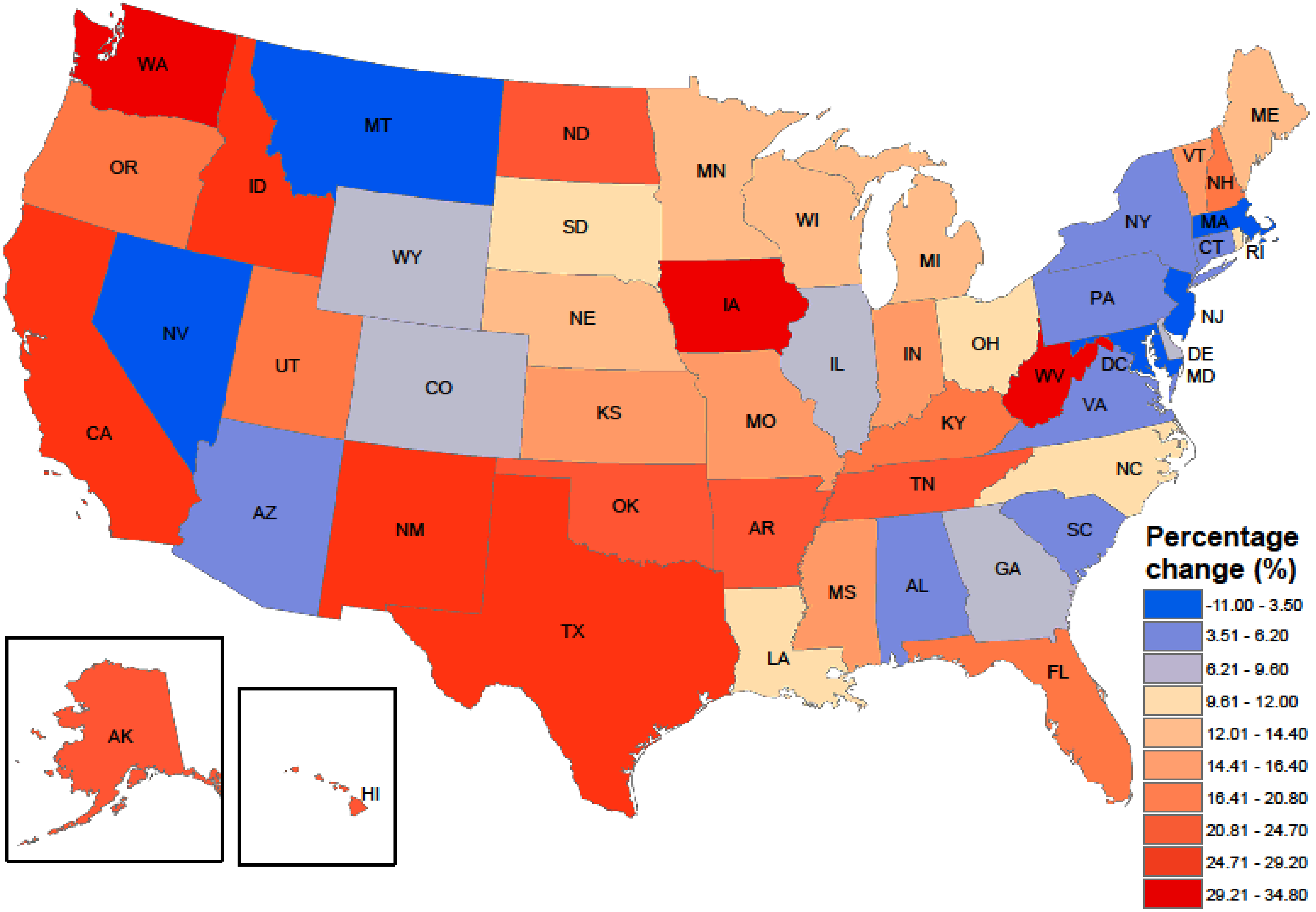

© Bowe B et al. *JAMA Network Open*

eFigure 3A: Decomposistion of change in DALYs from 2002 to 2016 due to chronic kidney disease by age structure, size of the population, and risk exposure.

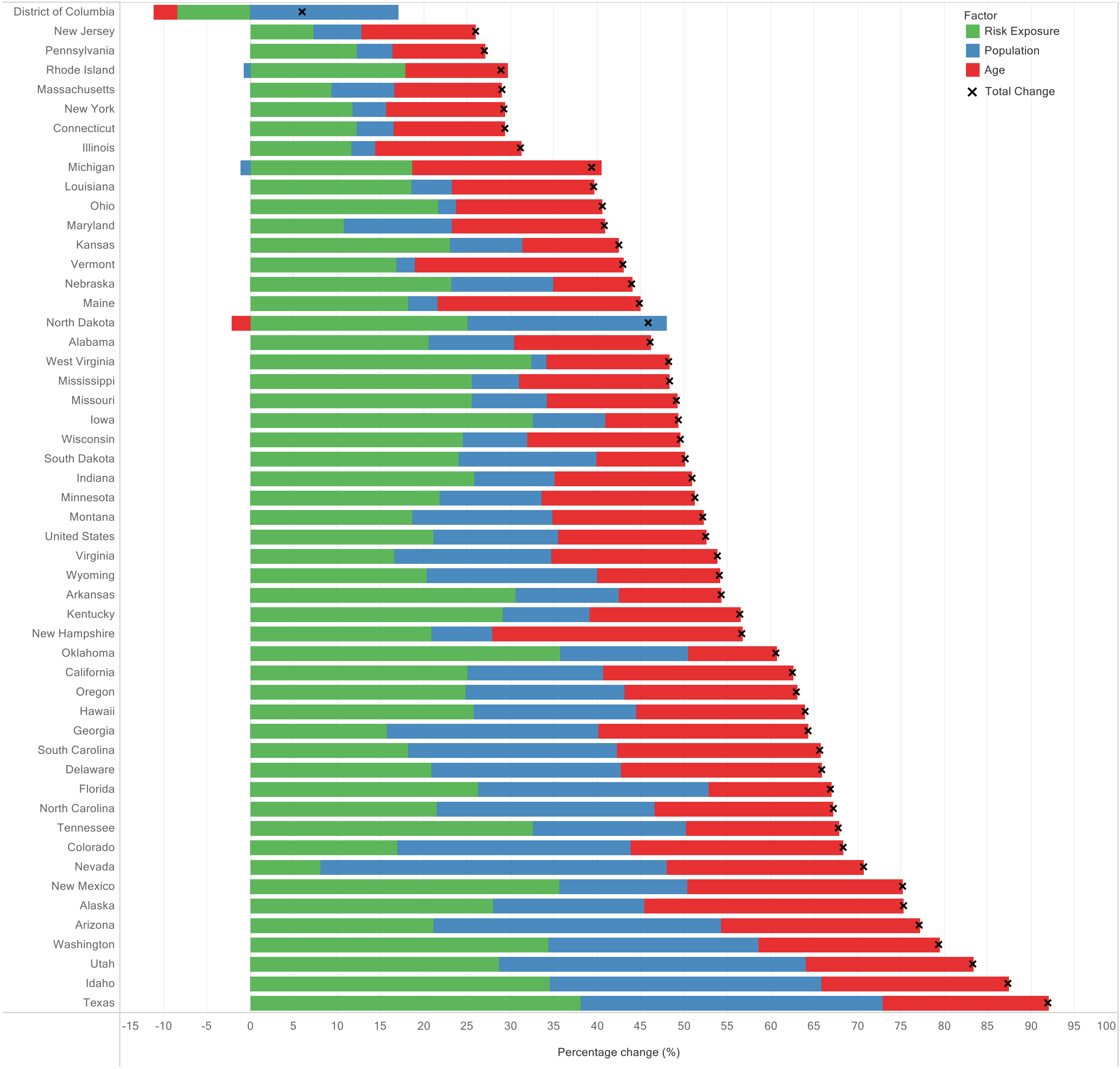

© Bowe B et al. *JAMA Network Open*

eFigure 3B: Decomposistion of the percentage change in deaths from 2002 to 2016 due to chronic kidney disease by age structure, size of the population, and risk exposure.

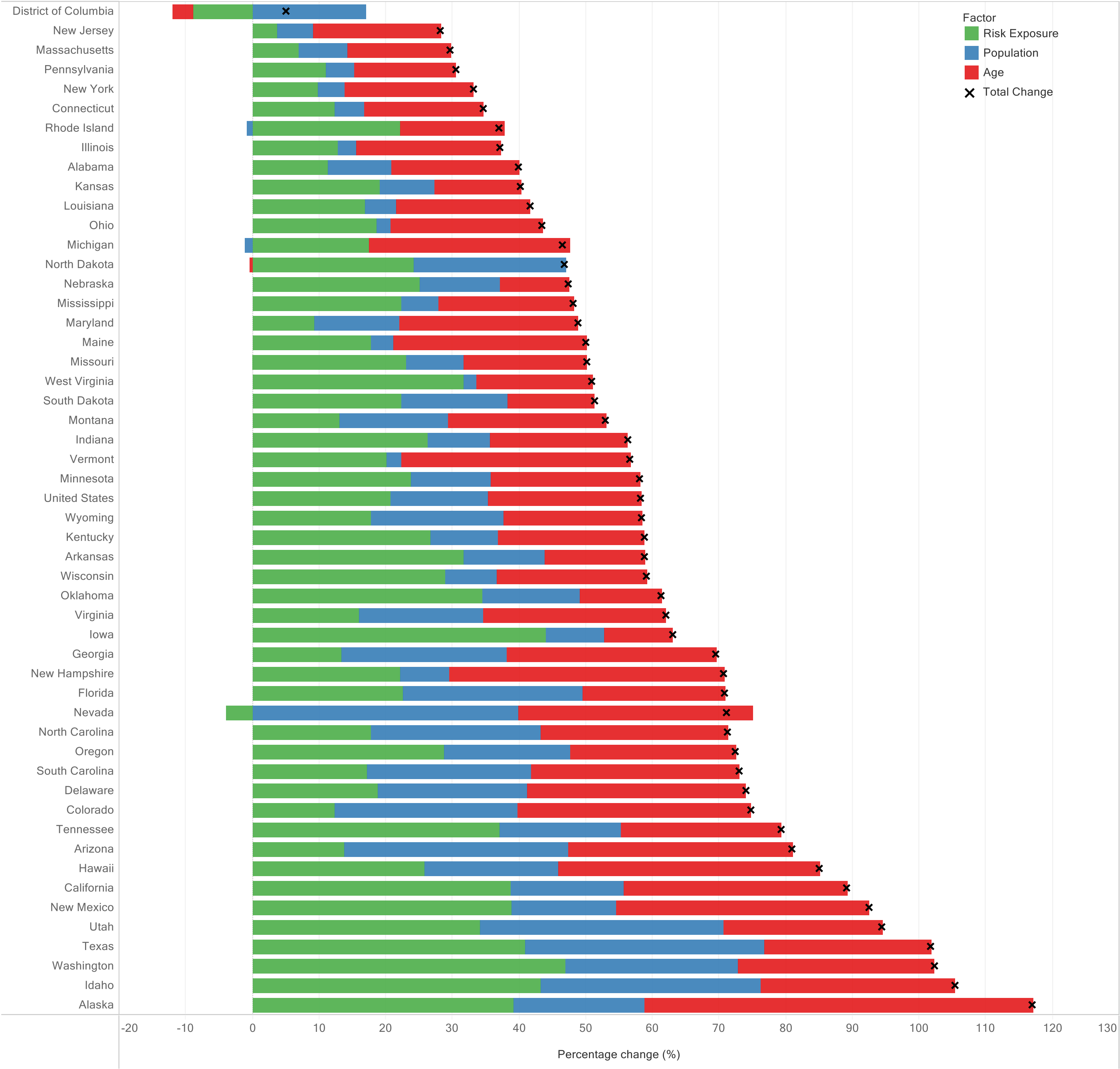

© Bowe B et al. *JAMA Network Open*

eFigure 4: Decomposistion of the percentage change in deaths from 2002 to 2016 due to chronic kidney disease by risk factor.

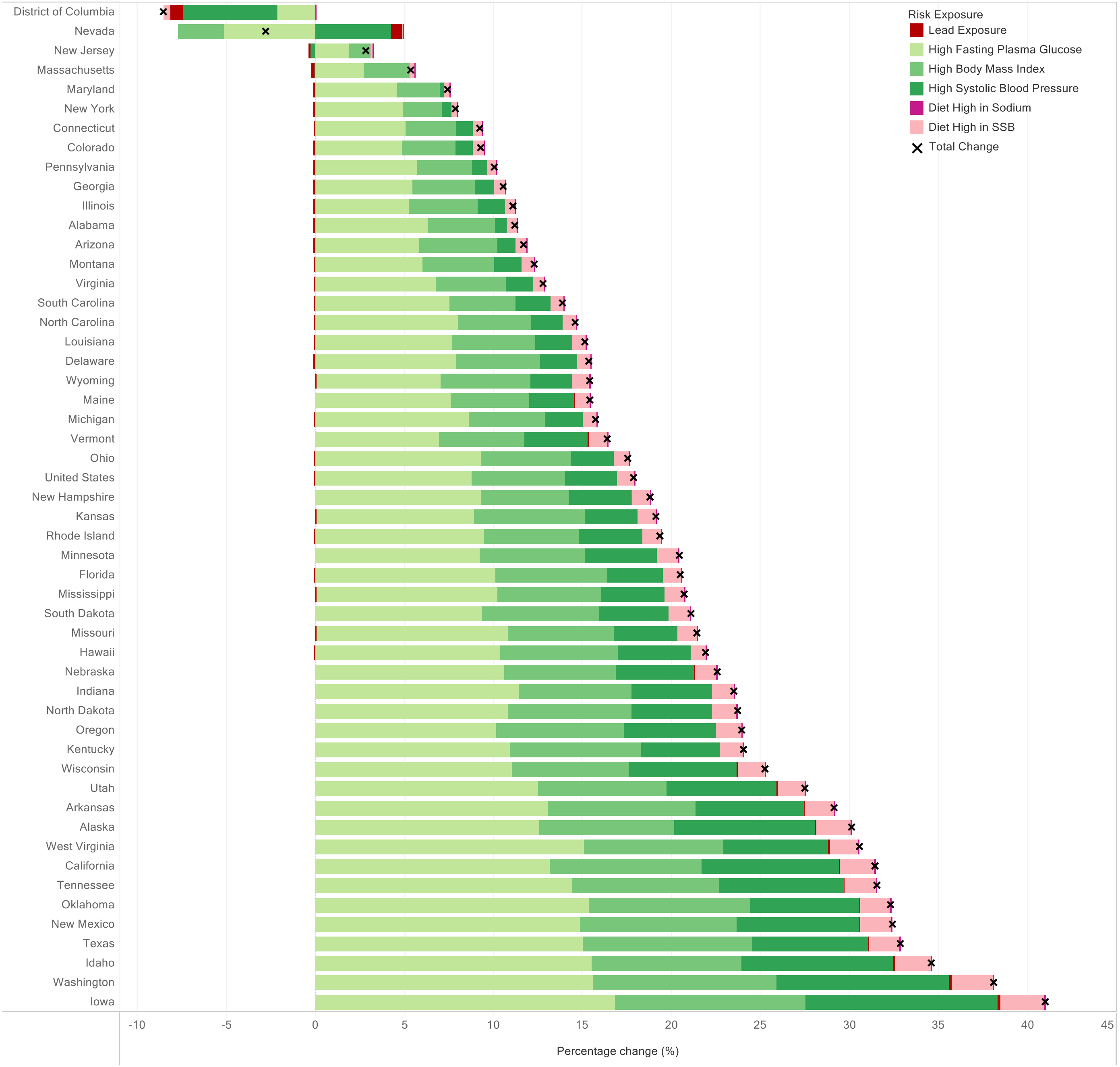

© Bowe B et al. *JAMA Network Open*

eFigure 5A: Decomposition of the percentage change in age-standardized DALY rates in the United States from 2002 to 2016 by cause.

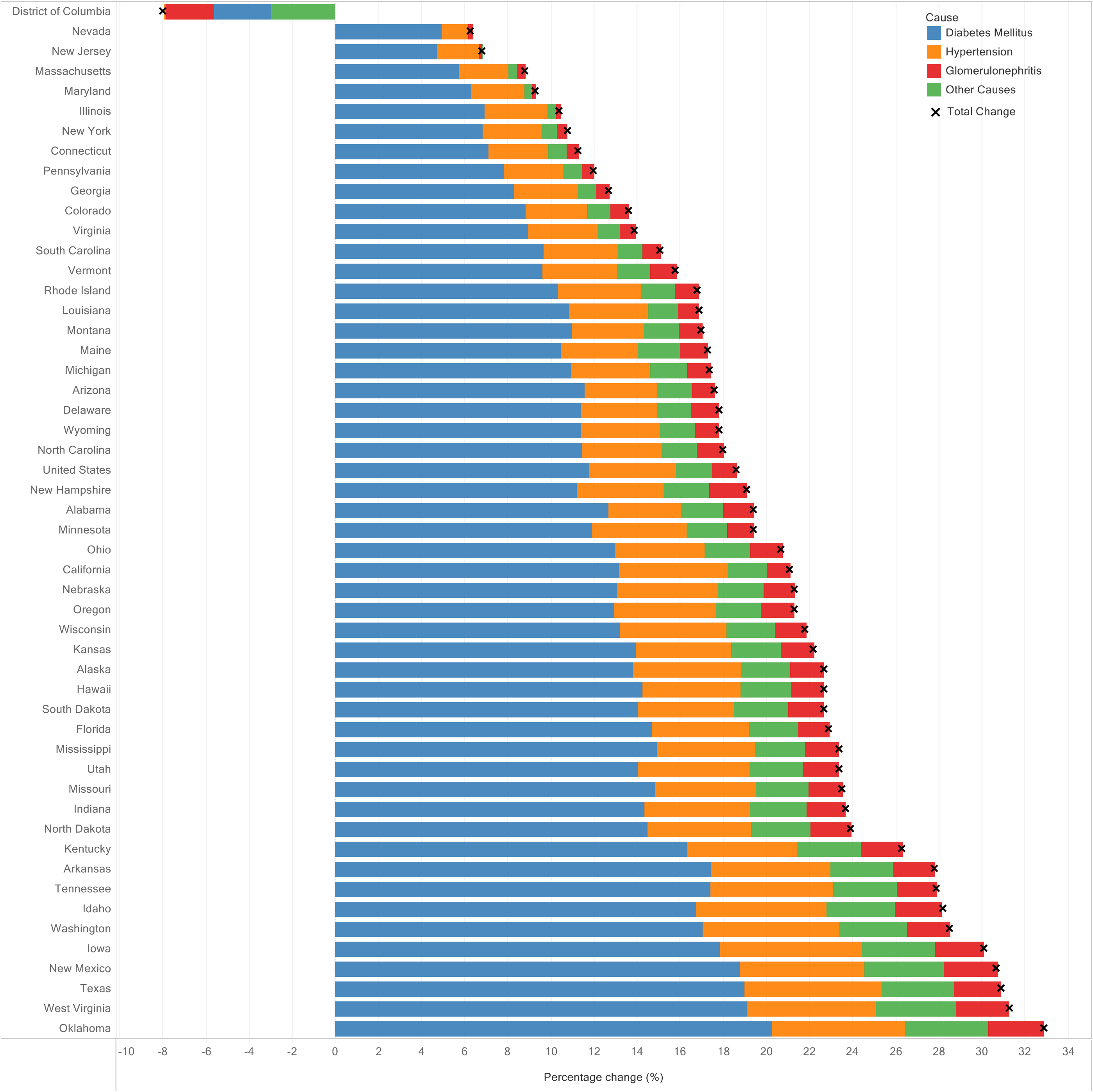

© Bowe B et al. *JAMA Network Open*

eFigure 5B: Decomposition of the percentage change in age-standardized death rates in the United States from 2002 to 2016 by cause.

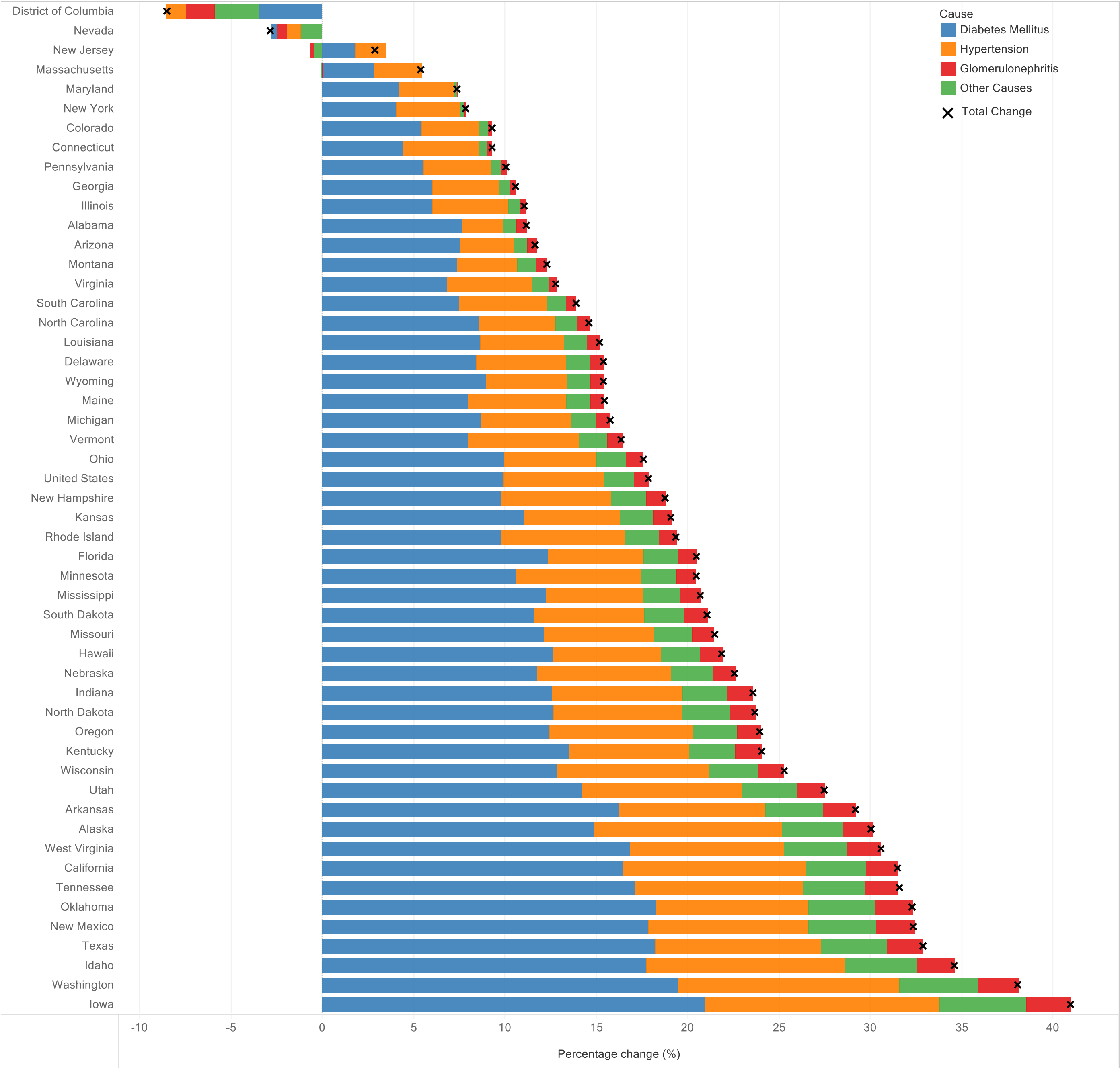

© Bowe B et al. *JAMA Network Open*

eFigure 6: Age-standardized disability-adjusted life year (DALY) rates due to CKD by socio-demographic index (SDI) in 2002 and 2016 for all states and one district.

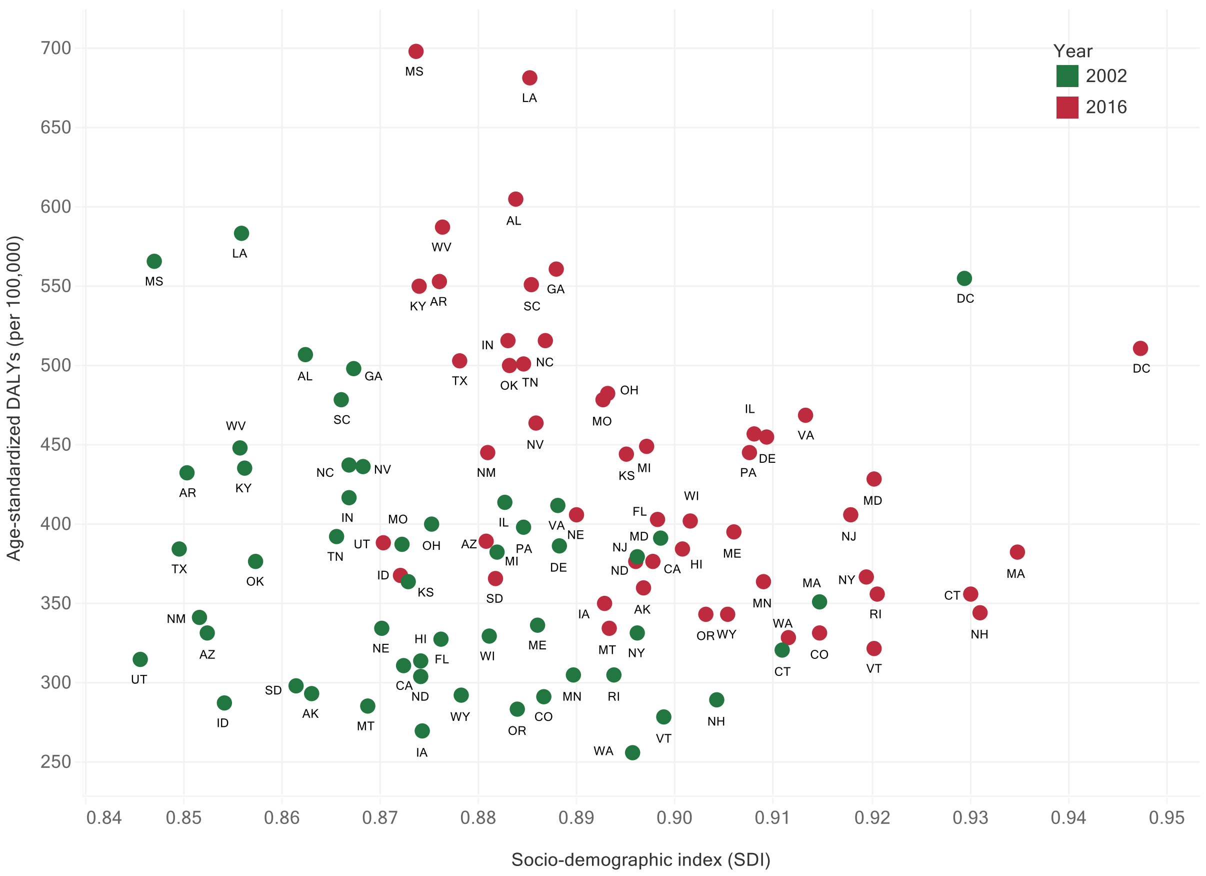

eFigure7: Observed to expected ratio of age-standardized disability-adjusted life year (DALY) rates due to CKD in 2016 based on a state or district's socio-demographic index (SDI).

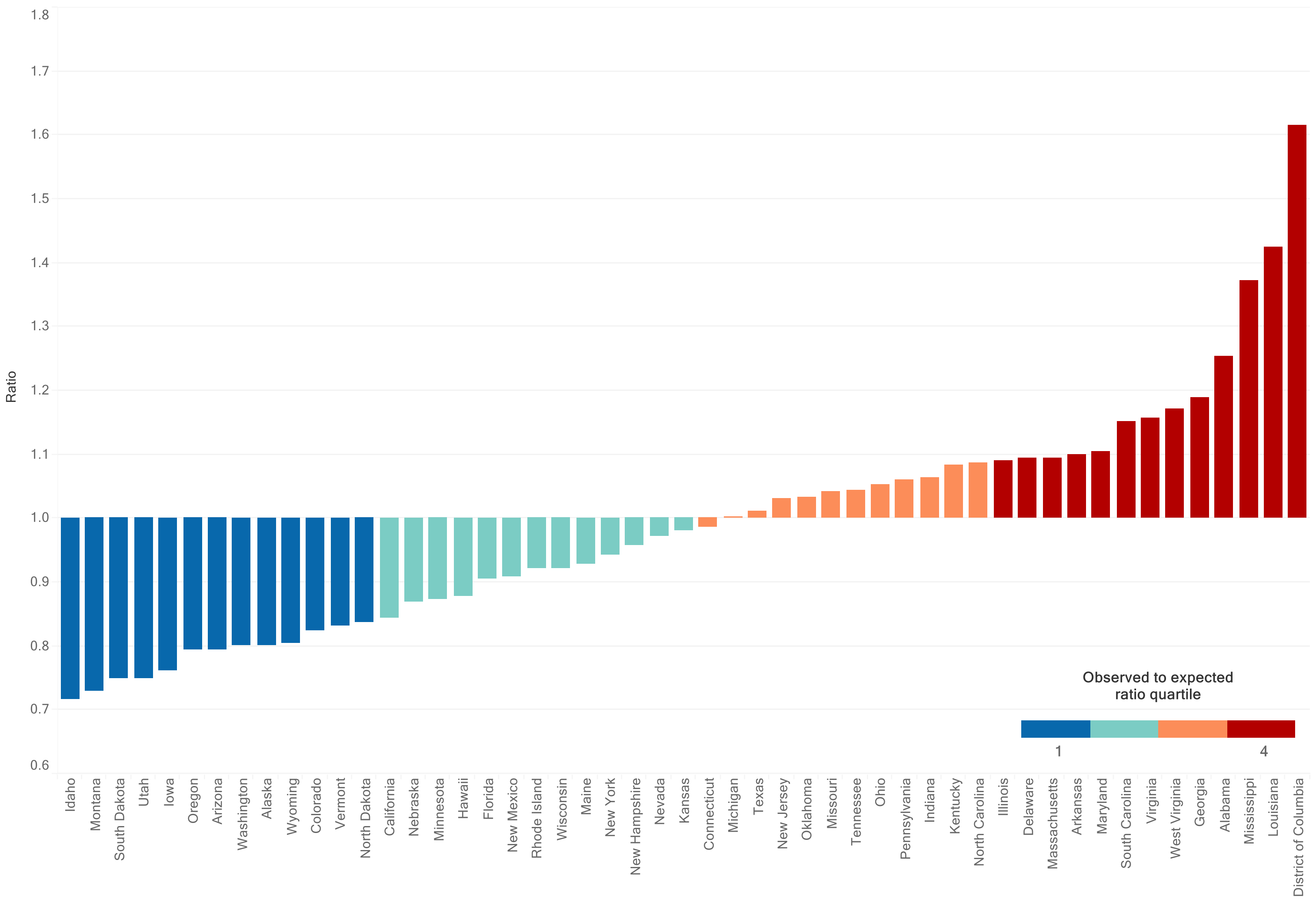

© Bowe B et al. *JAMA Network Open*

eFigure 8: Frontier analysis for age-standardized disability-adjusted life year (DALY) rates due to CKD by socio-demographic index (SDI) in 2016.

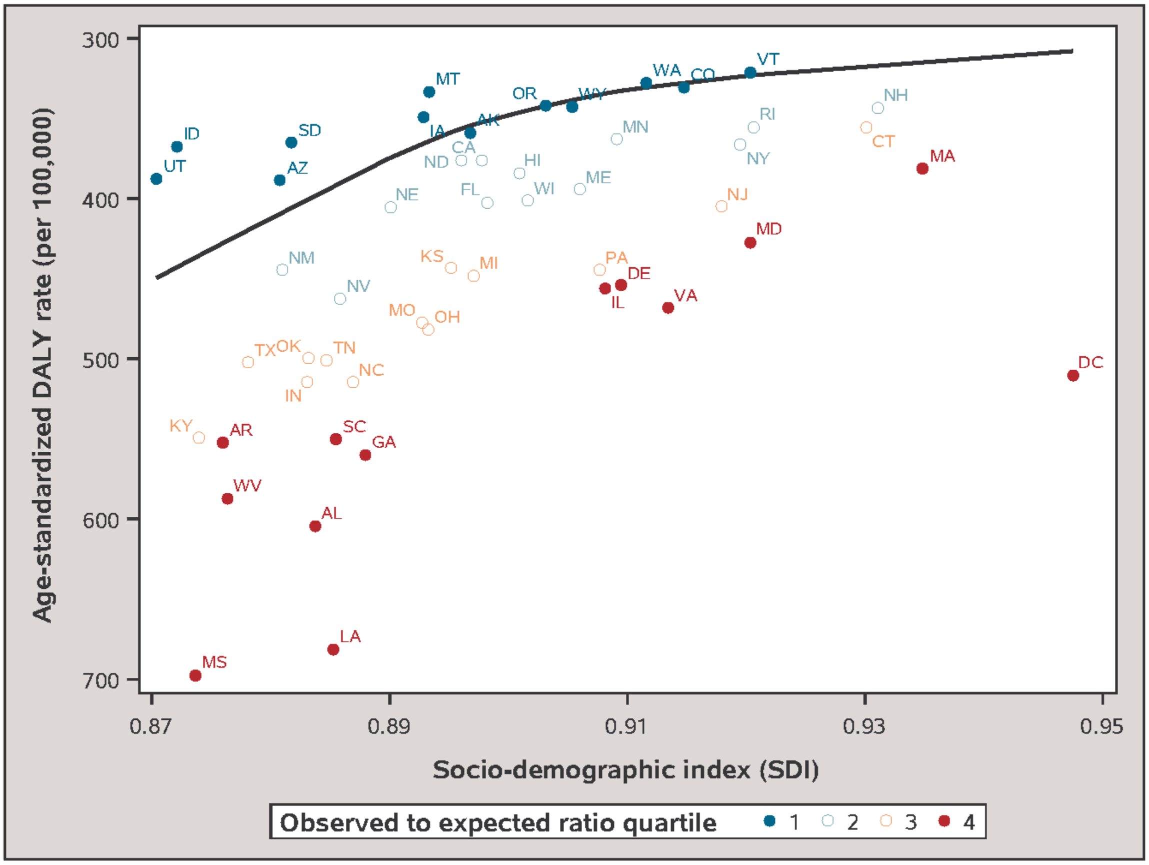

© Bowe B et al. *JAMA Network Open*
